# An expanded subventricular zone supports postnatal cortical interneuron migration in gyrencephalic brains

**DOI:** 10.1038/s41593-025-01987-2

**Published:** 2025-07-14

**Authors:** JaeYeon Kim, Aunoy Poddar, Kadellyn Sandoval, Julia Chu, Emma Horton, Di Cui, Keira Nakamura, I-Ling Lu, Michael Mui, Theresa Bartels, Christian M. Wood, Susana I. Ramos, David H. Rowitch, Nadejda M. Tsankova, Hosung Kim, Chet C. Sherwood, Boris W. Kramer, Angela C. Roberts, Pablo J. Ross, Duan Xu, Nicola J. Robertson, Elizabeth A. Maga, Peng Ji, Mercedes F. Paredes

**Affiliations:** 1 Department of Neurology, University of California, San Francisco, San Francisco, CA USA; 2 Eli and Edythe Broad Center of Regeneration Medicine and Stem Cell Research, University of California, San Francisco, San Francisco, CA USA; 3 Medical Scientist Training Program, University of California, San Francisco, San Francisco, CA USA; 4 Developmental and Stem Cell Graduate Program, University of California, San Francisco, San Francisco, CA USA; 5 Biomedical Sciences Graduate Program, University of California, San Francisco, San Francisco, CA USA; 6 Department of Radiology and Biomedical Imaging, University of California, San Francisco, San Francisco, CA USA; 7 Department of Neonatology, University of California, San Francisco, San Francisco, CA USA; 8 Department of Paediatrics, University of Cambridge, Cambridge, UK; 9 Wellcome–MRC Cambridge Stem Cell Institute, University of Cambridge, Cambridge, UK; 10 Department of Physiology, Development and Neuroscience, University of Cambridge, Cambridge, UK; 11 Behavioural and Clinical Neuroscience Institute, University of Cambridge, Cambridge, UK; 12 Department of Pathology, Molecular, and Cell-Based Medicine, Icahn School of Medicine at Mount Sinai, New York City, NY USA; 13 Department of Neuroscience, Icahn School of Medicine at Mount Sinai, New York City, NY USA; 14 Department of Pediatrics, University of California, San Francisco, San Francisco, CA USA; 15 Department of Neurology, USC Stevens Neuroimaging and Informatics Institute, Keck School of Medicine, University of Southern California, Los Angeles, CA USA; 16 Department of Anthropology Center for the Advanced Study of Human Paleobiology, The George Washington University, Washington, DC USA; 17 Department of Neonatology, Poznan University of Medical Sciences, Poznan, Poland; 18 Department of Animal Science, University of California, Davis, Davis, CA USA; 19 Institute for Women’s Health, University College London, London, UK; 20 Centre for Clinical Brain Sciences, University of Edinburgh, Edinburgh, UK; 21 Department of Nutrition, University of California, Davis, Davis, CA USA

**Keywords:** Development of the nervous system, Neuronal development

## Abstract

Cortical GABAergic interneurons generated in the ventral developing brain travel long distances to their final destinations. While there are examples of interneuron migration in the neonatal human brain, the extent of postnatal migration across species and how it contributes to cortical interneuron composition remains unknown. Here we demonstrate that neonatal gyrencephalic brains, including humans, nonhuman primates and piglets, harbor an elaborate subventricular zone, termed the Arc, due to its curved morphology and expanded neuroblast populations. The Arc is absent in lissencephalic marmoset and mouse brains. Transcriptomic and histological approaches revealed that Arc neurons are diverse interneurons from the medial and caudal ganglionic eminences that migrate into the frontal, cingulate and temporal cortex. Arc–cortical targets exhibit an increase in VIP^+^ neuronal density compared to other regions. Our findings reveal that the Arc is a developmental structure that supports the expansion of postnatal neuronal migration for cortical interneuron patterning in gyrencephalic brains.

## Abstract (web summary)

Neonatal gyrated brains harbor an elaborate subventricular zone, termed the Arc, supporting cortical migratory streams of diverse interneurons.

## Main

Neuronal migration is a critical process through which immature neurons reach their anatomical destinations in developing brains before integration into neuronal circuits^1,2^. This process is most robust in the prenatal period; however, there are restricted areas that maintain neuronal migration into the postnatal period^3^. One example is the subventricular zone (SVZ), a neurogenic region that resides in the cortical ventricular wall of mammalian brains. This area harbors inhibitory neuroblasts expressing doublecortin (DCX), a microtubule-associated protein fundamental to migration^4–6^. These neuroblasts travel via a rostral migratory stream (RMS) to the olfactory bulb (OB)^7–10^. Comparative studies of the postnatal mammalian SVZ suggest that this region has changed across phylogenetic lineages^11,12^. Neuroblasts in the rabbit brain, for example, organize into distinct clusters adjacent to the SVZ and extend into the dorsal parenchyma^13^. The human SVZ has evolved into a complex organization, giving rise to a transient structure called the Arc, whose interneurons target regions of the frontal cortex and the cingulate gyrus^14^. The piglet SVZ also has complex structural features, including a vascular substrate, suggesting more similarity to human SVZ^15,16^. Understanding the evolution of this structure and how it contributes to migratory populations of interneurons in the postnatal cortex is fundamental to defining the mechanisms of protracted cortical development in larger species.

Here we demonstrate that neonatal gyrencephalic brains, including humans, nonhuman primates and piglets, harbor an elaborate SVZ with complex cytoarchitectural features. This developmental structure, which we term the Arc, contributes to postnatal migratory streams of diverse interneuron subtypes. Arc-associated cortical regions, including the frontal and temporal cortices, have increased vasoactive intestinal peptide (VIP)^+^ neuronal density compared to other areas. The maintenance of postnatal migration for regional patterning of cortical interneuron composition is a developmental feature across gyrencephalic mammals. Its presence in phylogenetically divergent species suggests that this could be a universal principle of neurodevelopment across gyrencephalic mammals.

## Results

### A ventricular niche of GABAergic neurons in neonatal brains

**Fig. 1:**
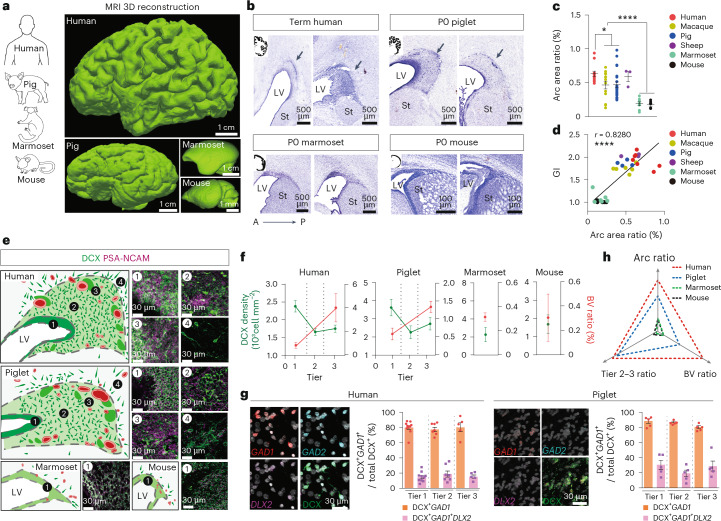
Arc structure is identified in neonatal gyrencephalic brains. **a**, MRI 3D reconstruction of the postnatal human, pig, marmoset and mouse brains. Two gyrencephalic brains (human at birth (term) and P0-aged piglet) and two lissencephalic brains (marmoset at 4.5 years of age and mouse at birth). **b**, Nissl-stained serial sections across species taken at birth. Black arrows indicate a cell-dense region extending dorsally from the lateral ventricle. **c**, Quantification of Arc area relative to total brain area (Arc area ratio, %) by Nissl staining at birth. Two-tailed unpaired *t* test (*P = 0.0268, ****P < 00001). The data are presented as mean ± s.e.m. Sample size is provided as source data. **d**, Correlation (Pearson’s *r*) between GI with the Arc area ratio across species (*r* = 0.8291, ****P < 0.0001). **e**, Arrangement of DCX^+^ cells (green) near the ventricular wall across species. Insets 1–4 (right of schematic) show confocal images of DCX^+^-enriched regions. Blood vessels are shown in red; dark green clusters correspond to DCX^+^ cellular densities. **f**, Quantification of DCX^+^ cells near the ventricular wall (green line). Quantification of BV areas, labeled by alpha smooth muscle actin (α-SMA), relative to total Arc area (BV ratio, %, red line) across species. The data are presented as mean ± s.e.m. Human (*n* = 4), piglet (*n* = 3), marmoset and mice (each *n* = 2). **g**, Left, confocal microscopy of single molecule fluorescence in situ hybridization (smFISH) for RNA expression of GABAergic-neuron markers (*GAD1*, *GAD2* and *DLX2*) in the neonatal human and piglet brain. DCX protein expression was codetected. Right, quantification of DCX^+^ *GAD1*^+^ cells of all DCX^+^ cells across tiers. Data means ± s.e.m., *n* = 2 individuals (each species) in three independent experiments. Sample size is provided as source data. **h**, Triangle plot showing a comparison of core Arc features across species. All values are normalized to humans (raw values are shown as source data), and two-way MANOVA analysis is shown in Supplementary Table 2. Red dotted line (human), blue dotted line (piglet), green dotted line (marmoset) and black dotted line (mouse). LV, lateral ventricle; St: striatum; BV, blood vessel. Source data

**Extended Data Fig. 1:**
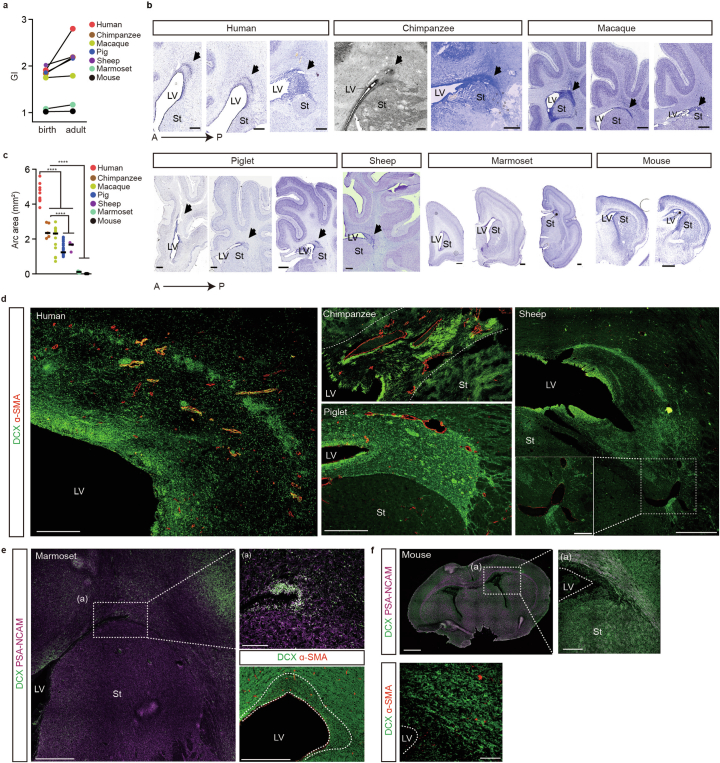
Comparative analysis of ventricular wall structure across species. **a**, Developmental changes in cortical folding as measured by gyrification index (GI) across species. Sample size is provided as source data. **b**, Nissl-stained serial coronal brain sections from different species collected at perinatal ages. The enlarged SVZ, termed the Arc, is present in humans, chimpanzees, macaques, and piglets at birth, embryonic day 135 (E135; gestational period = 150 days), sheep brains, but not in marmoset and mouse brains at birth. Arrows indicate the Arc. The macaque dataset is from the public open source (NIH Blueprint NHP Atlas). Scale bars, 500 µm (human, chimpanzee, macaque, pigletand marmoset); 1 mm (sheep); 100 µm (mouse). Lateral ventricle (LV); striatum (St). **c**, Quantification of Arc area (mm^2^) from Nissl-stained sections of different species. Two-tailed unpaired *t* test, ****p < 0.0001. n = 4 individuals (human); n = 1individuals (macaque and sheep); n = 3 individuals (piglet); n = 2 individuals (chimpanzees, marmoset and mouse). Data are presented as mean ± s.e.m. of counts performed on each individual case in three independent experiments. Sample size is provided as source data. **d**, Confocal images show robust expression of the migratory neuron marker DCX (in green) and an abundance of blood vessels expressing α-SMA (in red) in the perinatal human, chimpanzee, piglet, and sheep Arc. Scale bars, 500 µm; 100 µm (higher magnification images). **e**, Coronalsection of postnatal day 0 (P0) marmoset and mouse brains. Migratory neural populations expressing DCX (in green) and PSA-NCAM (in magenta) do not form a tiered structure, and vascular areas (α-SMA, red) are sparse in the ventricular wall of P0 marmoset and mouse brains. Scale bars, 500 µm; 100 µm (higher magnification images). Lateral ventricle (LV); striatum (St). This experiment has been repeated three times (**b**,**d**,**e**). Source data

We investigated the conservation of Arc structure at the ventricular wall of human, pig, marmoset and mouse brains. Magnetic resonance imaging (MRI) classified the neonatal human and pig (piglet) brains as gyrencephalic, having cortical surface infoldings, whereas marmoset and mouse brains were classified as lissencephalic, with a smooth surface, even at adult ages (Fig. 1a, Extended Data Fig. 1a and Supplementary Table 1)^17,18^. Serial section analysis of the neonatal human and piglet ventricular wall showed a cell-dense region, corresponding to the SVZ, bordering the ventral edge of the developing striatum; this was also observed in early postnatal marmoset and mouse brains. However, the human and piglet SVZ had cellular extensions from the dorsolateral wall into the overlying white matter (WM) region (Fig. 1b,c). The SVZ in other gyrencephalic species, chimpanzees and macaques (order Primates) and sheep (order Artiodactyla) harbored a similar dorsal extension (Extended Data Fig. 1b,c). Correlation analysis comparing the Arc area and gyrification index (GI), a measure of cortical folding, across six species showed that the Arc area was significantly associated with GI (Fig. 1d).

We previously demonstrated that the human Arc contains migratory cells expressing DCX and polysialylated neural cell adhesion molecule (PSA-NCAM), indicative of neuroblast identity. DCX^+^ cells were organized into the following four distinct layers^14^: DCX^+^ cells along the ventricular wall (tier 1), dispersed away (tier 2), around blood vessels (tier 3) and as clusters oriented toward the pia in the developing WM (tier 4). DCX^+^ cells were similarly arranged around the piglet ventricular wall, consistent with the presence of an Arc. This cellular distribution was similarly observed in the chimpanzee and sheep SVZ (Extended Data Fig. 1d and Supplementary Fig. 1). In contrast, DCX^+^ PSA-NCAM^+^ cells in marmoset and mouse brains were solely arranged tangentially along the ventricular wall, indicative of having only a tier 1-like structure (Fig. 1e and Extended Data Fig. 1e,f). Furthermore, the vascular area at the ventricular wall, a feature of Arc tier 3, was substantially reduced in P0 marmoset and mouse brains compared to humans, chimpanzees, piglets and sheep (Fig. 1f and Supplementary Fig. 2). DCX^+^ cells in the human and piglet Arc showed similar inhibitory representation, with 79.3% of DCX^+^ cells expressing *GAD1* in the human and 85.5% in the piglet. About 20% of DCX^+^ cells co-expressed *GAD1* and *DLX2* in both human and piglet Arc. This proportion was preserved across Arc tiers (Fig. 1g and Supplementary Fig. 3). Multivariate analysis of variation (MANOVA) of core Arc features confirmed that the neonatal human Arc was similar to the P0 piglet Arc (*P* = 0.11) and significantly different from P0 marmosets and mice (***P = 6.21 × 10^−7^ and ^***^P = 8.07 × 10^−5^, respectively; Fig. 1h and Supplementary Table 2).

**Extended Data Fig. 2:**
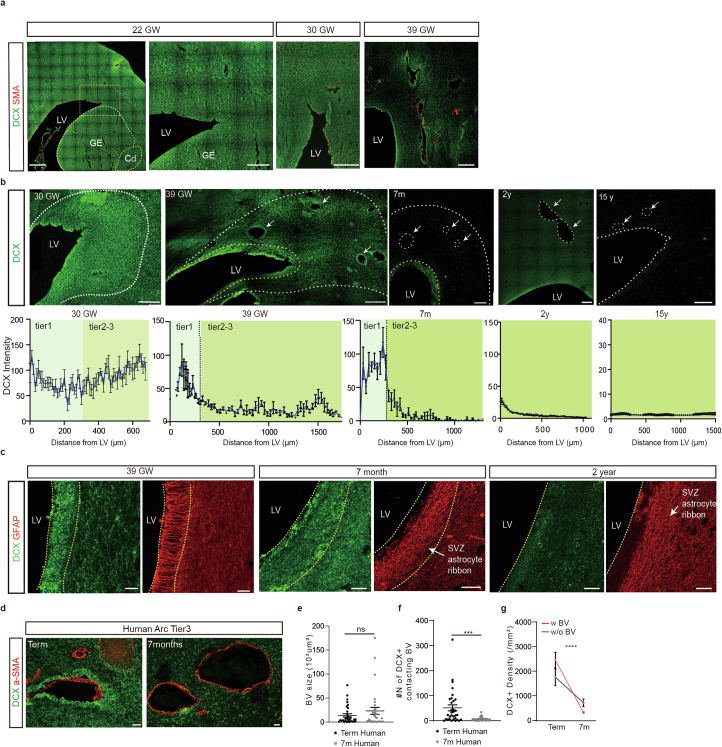
Cytoarchitectural changes in the human Arc during perinatal stages. **a**, Confocal images of Arc structural changes during the perinatal stages of humans. The higher magnification of the yellow boxed area is to the left of 22 GW. Scale bars, 1 mm (22 GW; left), 500 µm (22 GW; right, 30 GW and 39 GW). This experiment has been repeated four times. Lateral ventricle (LV); ganglionic eminence (GE); caudate nucleus (Cd). **b**, Top: confocal image of DCX^+^ expression (green) in a coronal section of human Arc from 30 GW to postnatal stages. The yellow and white dashed lines highlight the boundary between tiers 1–2 and tiers 3–4, respectively; arrows indicate blood vessels. Scale bars, 500 µm. Bottom: quantification of DCX expression pixel intensity across tiers. Data are presented as mean ± s.e.m. The serial slides from three individuals were taken for quantification. Sample size is provided as source data. **c**, Confocal images of tier 1 structural and cellular composition change from 39 GW to 2 years after birth. DCX^+^ neuroblasts (green) are densely populated with GFAP^+^ fibers (red) in tier 1 at 39 GW. Scale bars, 30 µm. This experiment has been repeated three times. **d**, Confocal images of DCX^+^ expression (green) near a-SMA^+^ blood vessels (red) in tier 3 of the human Arc across postnatal stages. Scale bars, 30 µm. **e**–**g**, Quantification of the BV size located in tier 3 (**e**), the total number of DCX^+^ cells in contact with the BV (**f**) and the density of DCX^+^ cells contacting with BV (w BV) and without BV (w/o BV) across ages (**g**). Two-tailed unpaired t-test, ***p = 0.0006 (**f**) and ****p < 0.0001 (**g**). Data are presented as mean ± s.e.m. of counts performed on different individual cases (n = 4, term; n = 1, 7 months) in three independent experiments. Sample size is provided as source data. Source data

**Extended Data Fig. 3:**
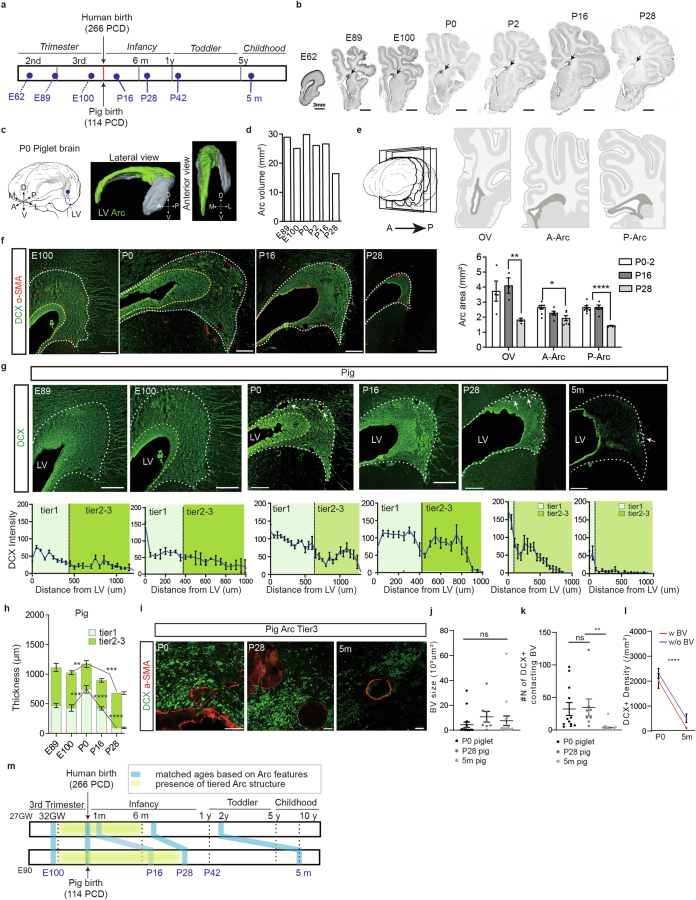
Structural changes in the piglet Arc during perinatal stages. **a**, Schematic comparing the stages of human and pig development^15,16^. PCD, post-conception day. **b**, Nissl stain of coronal section from piglet brains across ages. Scale bars, 3 mm. **c**, Schematic of the P0 piglet brain. The LV of the left hemisphere is highlighted in gray (left) and the Arc in green. Lateral ventricle (LV). **d**, The 3D volume of the Arc was measured from Nissl-stained piglet serial sections. **e**, Top: schematic showing piglet Arc subregions at coronal planes. Down: quantification of Arc area at each plane across ages. Two-tailed unpaired *t* test and data are presented as the mean ± s.e.m. (n = 3 cases). Olfactory ventricle (OV); anterior Arc (A-Arc); posterior Arc (P-Arc). Sample size is provided as source data. **f**, Confocal images of core Arc structural changes across ages. The yellow line highlights the boundary between tiers 1 and 2; the white line between tiers 3 and 4. Scale bars, 500 µm. This experiment has been repeated five times. **g**, Top: confocal image of DCX^+^ expression in developing piglet Arc. Arrows indicate blood vessels. Scale bars, 500 µm. Bottom: quantification of DCX expression pixel intensity across tiers. Data are presented as the mean ± s.e.m. (n = 3 cases). Sample size is provided as source data. **h**, Quantification of tier thickness in developing piglet brains. Scale bar, 500 µm. Two-tailed unpaired t tests and data as the means ± s.e.m. **i**–**l**, Confocal images of DCX^+^ cells near α-SMA^+^ blood vessels (BV) in tier 3 of the piglet Arc across ages. Scale bar, 30 µm. Quantification of the BV size (**j**), the total number of DCX^+^ cells in contact with the BV (**k**) and the density of DCX^+^ cells contacting with (w) and without (w/o) BV across ages (**l**). Two-tailed unpaired t tests and data as mean ± s.e.m. (n = 3 cases). Sample size and *P* values are provided as source data. **m**, Schematic showing Arc features age-matched between humans and piglets (blue lines) and the period when the tiered Arc structure is present in human and piglet brains (yellow lines). Source data

We next performed a spatiotemporal analysis in the human, piglet, marmoset and mouse to understand the developmental dynamics of Arc. At 22 gestational weeks (GW) of humans, when cortical folding emerges^19^, the structure of the Arc was not present (Extended Data Fig. 2a). We first observed the distinct distribution of DCX^+^ cells and vascular structures around the ventricular wall at 30 GW, when major sulci had already developed. The full-tiered arrangement was clearly organized at birth. DCX^+^ cells declined by 7 months, most dramatically within tiers 2–3, and completely disappeared by 2 years old (Extended Data Fig. 2b,c). The association of DCX^+^ cells with large-caliber vasculature in tier 3 also declined between birth and 7 months (Extended Data Fig. 2d–g). The temporal changes in the piglet Arc resembled those in the human SVZ. Tiered structures were first observed at E100 and were most clear at P0 (Extended Data Fig. 3a–f). DCX expression decreased between P28 and 5 months, most notably in tiers 2–3 (Extended Data Fig. 3g,h); this coincided with a decline in DCX^+^ clustering around vasculature (Extended Data Fig. 3i–l). Analysis of the prenatal marmoset and mouse brains did not show an Arc structure even at earlier ages (Supplementary Fig. 4). This analysis demonstrated that the Arc is a transient structure in the human and piglet brains (Extended Data Fig. 3m) and distinctive of gyrencephalic brains.

### Molecular diversity of GABAergic neurons in neonatal brains

**Fig. 2:**
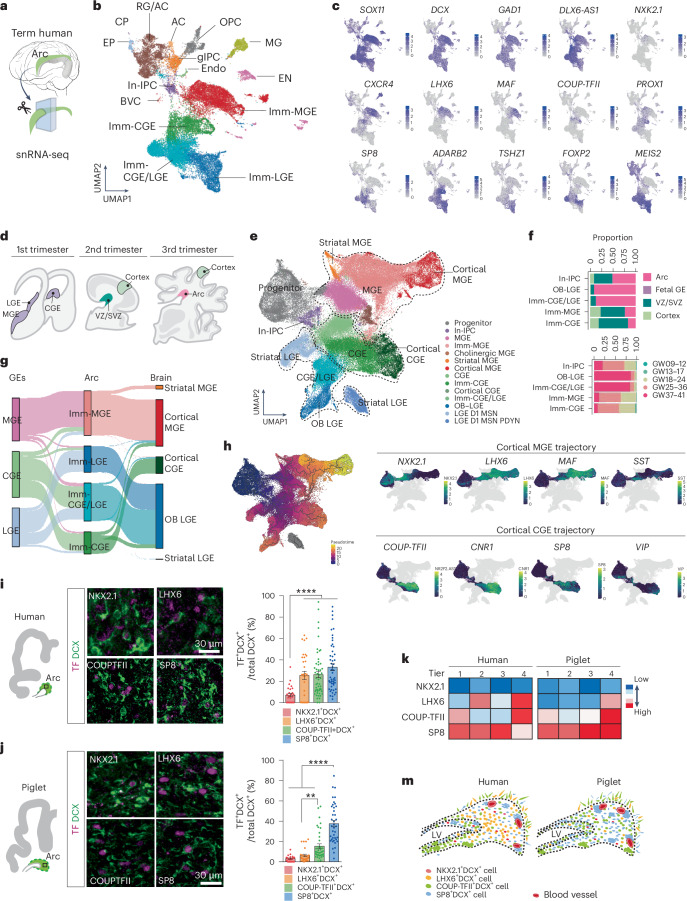
Diverse immature GABAergic neurons within the Arc. **a**, Schematic representation of snRNA-seq analysis from the human Arc at GW 30–39. **b**, Clustering of individual nuclei and visualized by UMAP. Annotation of 15 cell types based on gene expressions (see source data for details). **c**, The gene expression profile of well-known marker genes is visualized via UMAP. Cells are colored according to gene expression levels (purple, high; gray, low). **d**, Schematic representations of the dissected regions of the human brain across the trimesters. The primary sites of interneuron generation, MGE, LGE and CGE in the first trimester^22^. The VZ and neocortical regions in the second trimester^25^. The Arc and neocortical regions in the third trimester^25^. **e**, The cells of human ganglionic eminences (GEs), inhibitory cells of the developing VZ including Arc and developing neocortex are integrated and visualized by UMAP. **f**, The proportion of the nuclei from cell types across regions (top) and ages (down). **g**, Sankey diagram illustrating the origins and fates of the immature interneurons in the Arc. **h**, Left, the pseudotime of interneurons visualized by UMAP. Right, MGE-cortical trajectories (top) and CGE-cortical trajectories (bottom) are inferred from Monocle3. **i**,**j**, Left, subpopulations of DCX^+^ cells in the neonatal human (**i**) and piglet (**j**) Arc express different TF enriched in ventral telencephalic origins, immunostained with NKX2.1, LHX6, COUP-TFII and SP8. Right, quantification of DCX^+^ cells expressing the selected TFs. Two-way unpaired *t* test (**P = 0.0027, ****P < 0.0001). The data mean ± s.e.m. of counts performed on three individual cases (*n* = 3) in five independent experiments. Sample size and *P* values are provided as source data. **k**, Heatmap of distribution of DCX^+^ cells expressing different TFs across tiers. The color gradient represents TF expression levels from high (red) to low (blue), as quantified from the counts performed on each species. **m**, Schematic representation of the spatial distribution of molecularly distinct DCX^+^ cells in the neonatal human and piglet Arc. TF, transcription factors. Source data

**Extended Data Fig. 4:**
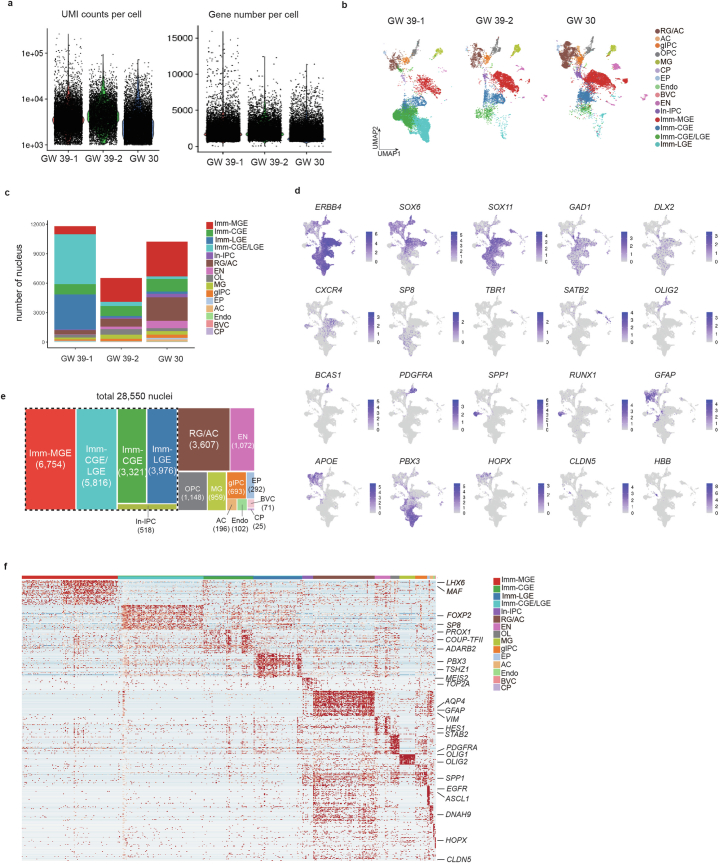
Transcriptomic profiles of neonatal human Arc cells. **a**, Quality control for the snRNA-seq dataset of human Arc samples. Arc samples were collected at GW 30 and GW 39 (GW 39-1 and GW 39-2). Each dot represents a single nucleus. Nuclei with mitochondrial gene fractions above 2% were discarded in the following analysis. The GW 39-1 sample represents the more anterior level dissected at GW 39, and the GW 39-2 sample comes from the more posterior level of Arc, which is equivalent to the dissected region at GW 30. **b**, The distribution of the nuclei across samples visualized in UMAP space. **c**, The proportion of 15 clusters from three samples is shown individually. **d**, The gene expression profile of well-known marker genes visualized via UMAP. *SOX6* and *SOX11*, markers of immature cells; *GAD1* and *DLX2*, markers of GABAergic inhibitory neurons; *TBR1* and *SATB2*, markers of excitatory neurons; *OLIG2*, *BCAS1* and *PDGFRA*, markers of oligodendrocytes; *SPP1* and *RUNX*, markers of microglia*; GFAP* and *APOE*, markers of astrocytes; *PBX3*, a marker of LGE-derived interneurons; *HOPX*, a marker for radial glia cells; *CLDN5*, a marker for endothelial cells; *HBB*, a marker for red blood cells; *ERBB4* and *CXCR4* are guidance receptors for the tangential migration of interneurons from the ganglionic eminence. **e**, Treemap showing the proportion of 15 subclusters based on gene expression. In total, 71% of nuclei express GE-associated TFs, indicating that Arc primarily harbors inhibitory populations. The dotted line includes inhibitory populations. **f**, Heatmap illustrating the differential gene expressions among 15 subclusters from 500 sampled cells. Radial glia cells with astrocyte characteristics (RG/AC); astrocyte (AC); glial intermediate progenitor cells (gIPC); IPC committed to inhibitory neuronal lineage (In-IPC); oligodendrocyte progenitor cells (OPC); microglia (MG); endothelial cells (Endo); immature excitatory neurons (EN); blood vessel cell (BVC); choroid plexus (CP); immature medial ganglionic eminence (Imm-MGE); caudal/lateral ganglionic eminence (CGE/LGE).

To explore the molecular features and cellular composition of the Arc, we performed single-nucleus RNA sequencing (snRNA-seq) on the human Arc using ventricular tissue dissected from three postmortem human brains between GW 30–39 (Fig. 2a). We obtained the transcriptional profiles of 28,550 single nuclei (Extended Data Fig. 4a–c). Using the community detection Leiden Algorithm, we defined 15 cell types in the Arc (Fig. 2b). Genes associated with GABAergic neurons, including *GAD1* and *DLX*2, *DLX5* and *DLX6*, were enriched in cell types that accounted for 71% of total nuclei (Extended Data Fig. 4d,e). These neuronal types were transcriptionally similar to three populations related to the medial, caudal and lateral ganglionic eminences (MGE, CGE and LGE, respectively), the primary sites for GABAergic-neuron production in the ventral embryonic brain^3,20^. We observed that transcriptomic features defined in the human prenatal ganglionic eminences^21,22^ continued to define GABAergic-neuronal types in the Arc. MGE-associated cell types in the Arc were characterized by the expression of *LHX6* and *MAF1*, CGE-associated cell types by the expression of *COUP-TFII*, also known as *NR2F2*, and *PROX1*, and LGE-associated cell types by *FOXP2*, *TSHZ1* and *MEIS2*. We identified another population expressing both CGE- and LGE-associated markers (*SP8*, *ADARB2*, *FOXP2* and *MEIS2)*, which we defined as a CGE/LGE cell type that may represent cells along a continuum between CGE- and LGE-related identities. Immature cell types expressed *SOX11* and *DCX;* therefore, we labeled this population as ‘immature neuron (Imm-)’^23,24^ (Fig. 2c). Interestingly, we identified intermediate progenitor cells committed to inhibitory neuronal lineage (In-IPC; *TOP2A*^+^*MKI67*^+^*DLX2*^+^*DCX*^+^), representing 2% of the total nuclei; these were distinct from the transit-amplifying cells (TACs) observed in the developing SVZ, which have excitatory contributions^25^ (Supplementary Fig. 5). The remaining cell types within the Arc contained radial glia (RG) cells with astrocyte characteristics, glial IPCs, oligodendrocyte progenitor cells, microglia, endothelial cells and immature excitatory neurons (EN; Extended Data Fig. 4f). This cellular composition was similar to what has been reported in the mouse and marmoset SVZ^26–28^.

**Extended Data Fig. 5:**
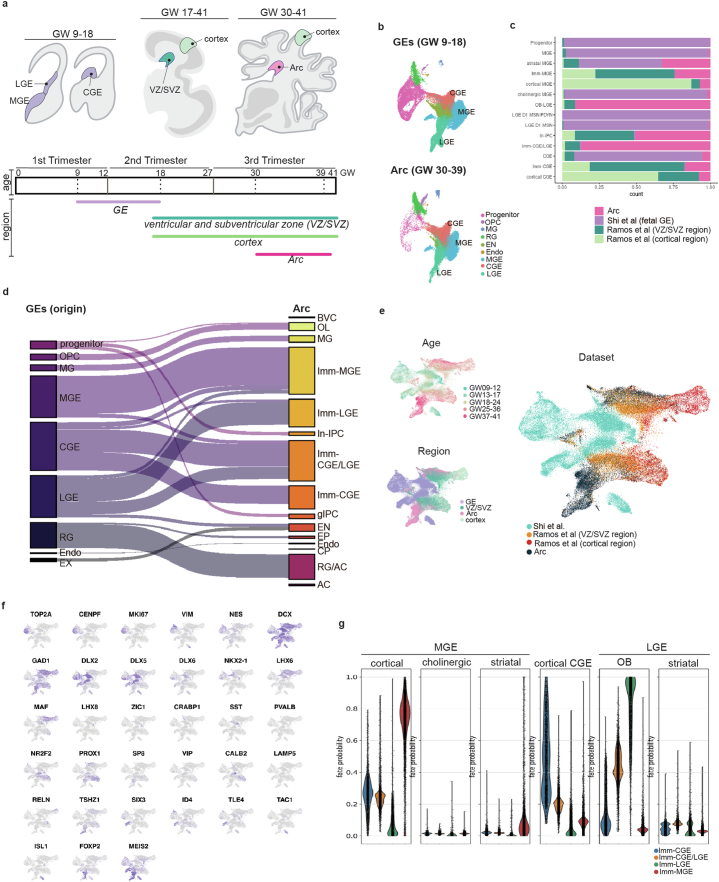
Arc is an intermediate structure in interneuron development, connecting the fetal GEs and cortical regions. **a**, Schematics of the age and dissected region of the human brain used in the dataset integration to study developmental features of Arc. It includes single-cell RNA sequencing datasets of human MGE, CGE and LGE from GW 9–18 (ref. ^21^), snRNA-seq datasets of the human germinal region near the LV and cortical plate from GW 17–41 (ref. ^25^), as well as our snRNA-seq datasets of the human Arc at GW 30–39. **b**, CCA integration of all cell types from human fetal GEs^21^ and term Arc, highlighting their inhibitory interneuron population similarity. **c**, Number of nuclei from cell types in each dataset. **d**, Sankey diagram derived from **b** shows the relationship between cells (nuclei) from the fetal GEs and the Arc. Flow thickness indicates the percentage of co-clustering between fetal GE populations and Arc populations. **e**, Clustering of individual cells (nuclei) from the different ages (left, top), different regions (left, bottom) and different datasets (right)^22,25^ visualized by UMAP. **f**, The gene expression profile of well-known marker genes visualized via UMAP. **g**, Fate probabilities inference of immature (Imm)-interneuron clusters from the Arc, highlighting that immature CGE neurons from the Arc primarily committed to cortical fate, while immature MGE neurons committed to cortical and striatal fates, and immature LGE neurons committed to olfactory bulb (OB) interneuron fate.

We integrated our GABAergic-neuronal dataset with published single-cell RNA-sequencing datasets of human MGE, CGE and LGE from GW 9–18 (ref. ^22^) to investigate the relationship between cells from the human fetal GEs and those from the Arc (Fig. 2d). We observed transcriptomic profiles of immature neurons from the Arc cocluster with those of neurons from human fetal GEs, indicating that immature neuronal types in the Arc share transcriptomic programs with those from interneurons in the human GEs (Extended Data Fig. 5a–d). Next, we sought to determine the developmental status of immature neurons from the Arc in the context of broader interneuron developmental trajectories. We expanded the integration to include snRNA-seq datasets of the human ventricular zone (VZ), including the developing SVZ, and neocortex from GW 17–41 (ref. ^25^; Fig. 2d). We found that immature GABAergic neurons from the Arc cluster primarily with neurons found in the prenatal VZ that are transitioning toward cortical interneuron fates (Fig. 2e,f and Extended Data Fig. 5e,f).

Next, we inferred the fate of human Arc immature interneurons using pseudotime and CellRank’s Markov chain model analyses. We defined cortical-, striatal- and OB-destined interneuron populations based on anatomical source and transcriptomic signature. Then, we computed the most probable fate of each Arc interneuron type, represented as flow weight in Fig. 2g. Immature MGE-associated and CGE-associated neurons from the Arc primarily differentiated into cortical interneurons^29^. We also identified the following three types of noncortical cell fates: MGE-associated striatal neurons, LGE-associated striatal neurons and LGE-associated OB interneurons. A subset of prenatal MGE cells and MGE-associated Arc cells were predicted to transition to MGE striatal cell fate. LGE striatal fate was associated only with prenatal LGE cells and lacked contribution from the Arc, suggesting an embryonic development. LGE-associated Arc cells transitioned to an LGE-derived OB neuronal fate (Extended Data Fig. 5g). Lineage trajectory analysis showed a high proportion of cells in the OB–LGE maturation trajectory originated from the Arc, indicating that Arc preserves the contribution of OB interneurons from SVZ (Supplementary Fig. 6). Additionally, Arc cells were present in cortical trajectories marked by transcription factors associated with MGE- and CGE-derived interneurons such as *LHX6*/*MAF1* and *COUP-TFII/SP8/CNR1* (Fig. 2h). Our subcluster analyses of the MGE- and CGE-associated interneuron subtypes within the Arc revealed transcriptomic diversity for their subclasses with potential cortical fates. Subanalysis of MGE-associated Arc nuclei revealed *CRABP1*^+^*NKX2.1*^*+*^ striatal cells and the following two cortical subclasses: *LHX6*^+^*SST*^+^ neurons and *LHX6*^+^*MEF2C*^+^ neurons. *MEF2C*^+^ cells have been shown to give rise to *PV*^+^ neurons^30^ (Supplementary Fig. 7a–c). CGE-associated nuclei could be divided into several subpopulations characterized by their expression of *COUP-TFII*—*VIP*^+^*CALB2*^*+*^, *CALB2*^+^, *RELN*^+^, *SP8*^+^*CCK*^+^ cell types (Supplementary Fig. 7d–f).

To investigate the genes that are co-expressed during GABAergic-neuronal maturation, we performed a weighted gene co-expression network analysis (hdWGCNA)^31^. We identified 12 modules of co-expressed genes with correlated expressions (Supplementary Fig. 8a–c). Genes in modules 1 and 12 were enriched in progenitors and associated with proliferation. Genes in modules 5 and 8 were strongly associated with immature neurons and related to cellular migration (*ERBB4*, *CXCR4* and *VLDLR*) and cell–cell adhesion (*DSCAM* and *GRID2*), respectively. Genes in module 2 were highly expressed in mature neurons and associated with synaptic transmission. Arc cells highly expressed genes in modules 5 and 8, consistent with their immature, migratory status (Supplementary Fig. 8d–i).

**Extended Data Fig. 6:**
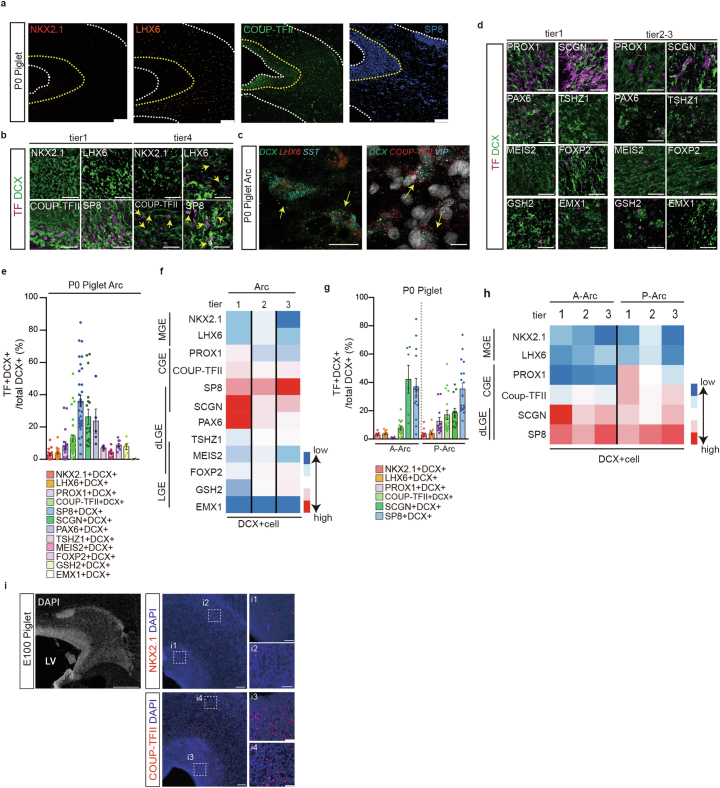
Diverse GABAergic-neuron subtypes within the piglet Arc. **a**, Widefield images of the P0 piglet Arc, immunostained with antibodies against GE-associated transcription factors. The yellow dashed line highlights the boundary between tiers 1–2; the white dashed line between tiers 3–4 (outer) or between tier 1 and the lateral ventricle (inner). Scale bars, 100 µm. **b**, High magnification images of the Arc expressing DCX and transcription factors (TF). Yellow arrows indicate DCX^+^ cells expressing TFs. Scale bars, 20 µm. **c**, RNA-scope images showing a subset of *DC*^+^*LHX*^+^*SST*^+^ and *DC*^+^*COUP-TFI*^+^*VIP*^+^ within the piglet Arc. Yellow arrows indicate *DCX*^+^ cells co-expressing other markers. Scale bars, 30 µm. **d**, High magnification images of the P0 piglet Arc, immunostained with antibodies against additional TFs enriched in the GE. Scale bars, 15 µm. **e**, Quantification of subpopulations of DCX^+^ cells in the P0 piglet Arc expressing different TFs enriched in GE. Data mean ± s.e.m. of counts performed on three cases (n = 3). **f**, Heatmap depicting the spatial distribution of DCX^+^ cells expressing different TFs across the tiers of the P0 piglet Arc. The color gradient represents TF expression levels from low (blue) to high (red). Medial GE (MGE). **g**,**h**, Quantification of the subpopulations of DCX^+^ cells across anterior–posterior divisions of the Arc in the P0 piglet brain (**f**) and corresponding heatmap (**g**). Data mean ± s.e.m. of counts performed on three cases (n = 3). For **e**–**h**, sample size is provided as source data. **i**, Left: widefield image (by DAPI) showing the early Arc at E100. Right: immunostaining with NKX2.1 and COUP-TFII antibodies. The dashed white boxes (**i**1–**i**4) are magnified. The early Arc at E100 is more populated by COUP-TFII^+^ cells than NKX2.1^+^ cells. Scale bars, 500 µm (widefield images), 30 µm (higher magnification images). This experiment has been repeated three times (**a**–**c**,**i**). Lateral ventricle (LV). Source data

Next, we histologically validated the cellular subpopulations and investigated the spatial distribution of interneuron subtypes within the human and piglet Arc (Supplementary Fig. 9). Consistent with our transcriptomic analysis, the human Arc contained DCX^+^ cells expressing LHX6, COUP-TFII and SP8 (refs. ^24,32^). In human Arc, 25.4% of DCX^+^ cells expressed LHX6, notably located in tier 2, and its subpopulation co-expressed SST (Fig. 2i,k and Supplementary Fig. 10). However, only 3.8% of DCX^+^ cells were LHX6^+^ in piglet Arc, even in embryonic ages, suggesting a notable species difference between the human and piglet (Extended Data Fig. 6 and Supplementary Fig. 11). In human Arc, 26.6% of DCX^+^ cells were COUP-TFII^+^, which were populated in tier 1, and a subset of COUP-TFII^+^DCX^+^ cells co-expressed with VIP (Supplementary Fig. 10). Although the COUP-TFII^+^DCX^+^ cell population was smaller at 15.4% in piglet Arc, its regional distribution and co-expression with VIP were preserved (Fig. 2j). SP8^+^DCX^+^ cells were present throughout the four tiers in both humans and piglets and the most abundant subpopulation within the piglet Arc. These markers were observed in the outer Arc, tier 4, of both species (Fig. 2k). Altogether, our analysis revealed the molecular diversity of immature interneurons within the human and piglet Arc, which are distributed across the Arc organization and converge at tier 4 (Fig. 2m).

### Emergence of postnatal migratory streams from the Arc

Given the transcriptomic trajectories of Arc neurons, we hypothesized that the Arc structure gives rise to distinct migratory streams destined for multiple targets. We used the piglet brain to interrogate the full hemisphere for migratory pathways. Sagittal serial sections of the P0 piglet brain revealed DCX^+^PSA-NCAM^+^ neuroblasts from the anterior olfactory ventricle connected to the OB, defining it as the RMS; this was consistent with our transcriptomic data linking Arc cells with OB neurons. There was also a thin medial stream that led to the ventromedial region of the prefrontal cortex (PFC). Lastly, migratory populations of DCX^+^ cells were found at the dorsal edge of the Arc, oriented toward the frontal cortex (Supplementary Fig. 12a,b). These populations were analogous to those observed in neonatal human brains^33,34^.

**Fig. 3:**
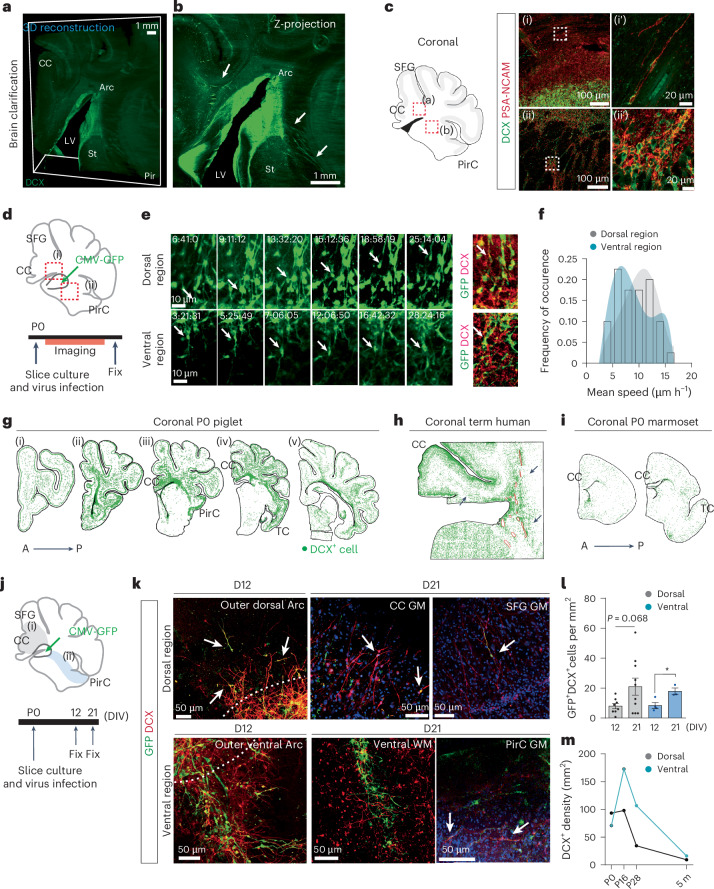
The Arc provides dorsal and ventral cortical streams of migratory neurons. **a**, Three-dimensional reconstruction of light sheeting imaging of a clarified P0 piglet brain with a 2.3 mm thickness (13 × 16 × 2.3 mm). **b**, Two-dimensional image is a z projection of the 3D image from **a**. The green signal is DCX immunolabeling. The arrows indicate multiple streams of DCX^+^ cells from the Arc. **c**, Left, schematic of coronal section of the P0 piglet brain. Right, DCX^+^PSA-NCAM^+^ neurons as individual neurons in the dorsal Arc (i) and as cell clumps in the ventral Arc (ii). **d**, Experimental design for time-lapse imaging of the P0 piglet brain. **e**, Left, sequential images of time-lapse confocal microscopy showing GFP^+^ cells in the dorsal region (top) and the ventral region (bottom) of the piglet Arc. White arrows highlight GFP^+^ cells near the Arc. Right, confocal image of GFP^+^DCX^+^ cells after 72 h of imaging. **f**, Distribution of mean migratory speeds measured for the two populations of GFP^+^ cells from the dorsal region (*n* = 21 cells; gray) and ventral region (*n* = 20 cells, blue). *n* = 3 individuals (**e**,**f**) in three independent experiments. **g**–**i**, Mapping of DCX^+^ cells (green) in the P0 piglet brain (**g**), the term human coronal sections (**h**), at a plane that corresponds to (iv in **g**), and the P0 marmoset brain (**i**). **j**, Experimental design of extended time-lapse imaging performed on P0 pig brain slices. **k**, Confocal images of GFP^+^DCX^+^ cells in the dorsal (top) and ventral (bottom) regions at 12 and 21 DIV. White arrows indicate GFP^+^DCX^+^ cells. (i) and (ii) indicate dorsal and ventral regions, respectively, in **d** and **j**. **l**, Quantification of GFP^+^DCX^+^ cell density in the dorsal and ventral regions at 12 and 21 DIV. Two-way unpaired *t* test (*P = 0.0234). Data means ± s.e.m. of counts performed on *n* = 3 cases in three independent experiments. **m**, Quantification of DCX^+^ cell density in dorsal and ventral regions across different postnatal ages of the pig, equivalent to regions analyzed in **j**. This experiment has been repeated three times (**a**–**c** and **e**).

Light-sheet imaging of the clarified P0 piglet brain permitted the scalable investigation of DCX^+^ neuroblasts along the entire Arc (Fig. 3a). The three-dimensional (3D) reconstruction showed collections of neuroblasts at the outer edge of the Arc (tier 4; Fig. 3b and Supplementary Video 1). Neuroblasts along the dorsal side were individual cells with elongated morphology and radial orientation directed toward the developing cortex. DCX^+^ cells at the ventral edge of the Arc were organized as long clumps that stretched into lateral–ventral regions (Fig. 3c and Supplementary Fig. 12c–g). Ventral DCX^+^ collections were often surrounded by brain lipid-binding protein (BLBP)^+^ or glial fibrillary acidic protein (GFAP)^+^ cells; this relationship was not observed with individually migrating DCX^+^ cells in the dorsal region (Supplementary Fig. 12h,i and Supplementary Videos 2 and 3).

To evaluate whether Arc neuroblasts indeed migrate in the postnatal cortical environment, we performed time-lapse confocal imaging on P0 piglet organotypic slice cultures. CMV-GFP adenovirus was microinjected into the Arc region of coronal sections (Fig. 3d). Adenovirus-derived GFP was expressed at 1 day in vitro (DIV; Supplementary Fig. 13a). At 2 and 3 DIV, we observed GFP^+^ cells in both the dorsal and ventral subregions with a small, elongated nucleus and a leading process directed away from the Arc (Fig. 3e and Supplementary Videos 4 and 5). Post hoc immunostaining of these slices after time-lapse imaging showed that migratory GFP^+^ cells were DCX^+^ (Fig. 3e and Supplementary Fig. 13b). The cells in the dorsal and ventral outer Arc did not differ in speeds, having an average of 8.82 µm h^−1^ and 7.5 µm h^−1^, respectively (Supplementary Fig. 13c). Migratory profiles and frequency distribution of mean speed suggested a trend toward more heterogeneous behaviors in the ventral subpopulation (Fig. 3f and Supplementary Fig. 13d). Similar migratory features were observed when Arc slice cultures were labeled with CAG-GFP adenovirus^35^ (Supplementary Fig. 14).

**Extended Data Fig. 7:**
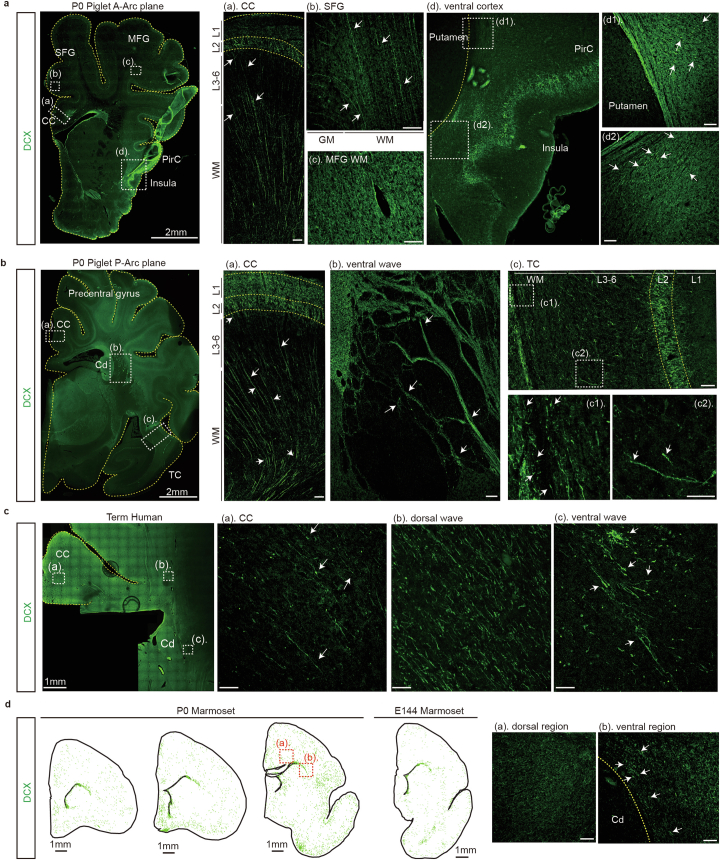
Dorsal and ventral migratory streams from the Arc target distinct cortical regions in neonatal human and piglet brains. **a**, Coronal section of the P0 piglet brain, at the level of the anterior Arc (A-Arc; refer to Extended Data Fig. 3e), immunostained for DCX. (a–d) High magnification images of the boxed areas in **a** (left) show that DCX^+^ cells migrate out from the Arc to the cingulate cortex (CC (a)), superior frontal gyrus (SFG (b)) and ventral cortex (piriform cortex (PirC) and insula (d)) but less so to the middle frontal gyrus (MFG (c)). Arrows indicate DCX^+^ cells with elongated, migratory morphology. Scale bars, 2 mm (**a**, left), 100 µm (a,d) and 50 µm (b,c,d1,d2). Gray matter (GM); layer (L); white matter (WM). **b**, Coronal section of the P0 piglet brain, at the level of the posterior Arc (P-Arc; refer to Extended Data Fig. 6e), immunostained for DCX. (a–c) High magnification images of the boxed areas in **b** (left). DCX^+^ cells migrate into the posterior CC (a) and temporal cortex (TC (c)). Arrows indicate DCX^+^ cells with elongated, migratory morphology. Scale bars, 2 mm (**b**, left), 100 µm (a–c) and 25 µm (c1,c2). Caudate nucleus (Cd). **c**, Coronal section of a term human brain, immunostained for DCX at a plane equivalent to the P-Arc plane of the P0 piglet brain illustrated in **b**. (a,b) DCX^+^ cells appear individually with an elongated morphology (arrows), migrate into the posterior CC (a) and are densely packed in the dorsal white matter (b). (c) DCX^+^ cells near the ventral Cd exist in clumps. Scale bars, 1 mm (**c**, left), 50 µm (a–c). **d**, Spatial mapping of DCX^+^ cells (in green) in serial coronal sections of P0 and a preterm marmoset brain; few DCX^+^ cells (arrows) are present in the dorsal (a) and ventral side (b) of the ventricular wall. Scale bars, 1 mm (map), 30 µm (confocal images). This experiment has been repeated five times (**a**–**d**).

Next, we performed DCX^+^ mapping from the Arc of P0 piglet brain (Fig. 3g). The dorsal Arc gave rise to a DCX^+^ stream that connected with the cingulate cortex (CC) and superior frontal gyrus (SFG; Extended Data Fig. 7a). DCX^+^ cells from the ventral Arc extended as chains of cell clumps that separated into individual cells within the piriform cortex (PirC) and temporal cortex (TC; Extended Data Fig. 7b). DCX^+^ neuroblasts in the neonatal human brain had both dorsal and ventral extensions from the Arc into adjacent cortical regions (Fig. 3h and Extended Data Fig. 7c). These cortical trajectories were also marked by the expression of SCGN, which is consistent with our transcriptomic analysis that *SCGN* is highly expressed in migratory neurons. We found similar cortical migratory streams derived from the Arc in chimpanzees and sheep brains (Supplementary Fig. 15). In contrast, the prenatal and P0 marmoset brain did not have clear migratory streams to the cortex; instead, there were few individual DCX^+^ or SCGN^+^ cells within the periventricular ventral and dorsal SVZ (Fig. 3i and Extended Data Fig. 7d) as previously reported^12^.

We tracked GFP^+^ cells over 21 DIV from the piglet Arc (Fig. 3j). GFP^+^DCX^+^ cells exited the dorsal Arc as individual cells and had migratory morphology with a small, elongated nucleus and a leading process with an average length of 128.5 µm; GFP^+^DCX^+^ cells at the ventral Arc collectively migrated as cell clumps. At 21 DIV, GFP^+^DCX^+^ cells were observed in CC and SFG gray matter (GM), ventral WM and PirC (Fig. 3k). The leading process of GFP^+^DCX^+^ cells within the CC/SFG WM was substantially increased with an average length of 187.9 µm. Interestingly, 80.9% of GFP^+^DCX^+^ cells that integrated into the GM of CC/STG exhibited more complex morphology with ramified apical dendrites (Supplementary Fig. 16). This suggests that the leading process of migratory neurons evolves as they migrate in the postnatal environment, and migratory neurons integrated within the cortical GM are differentiated into morphologically complicated neurons. Quantification of GFP^+^DCX^+^ cells supported their influx into both dorsal and ventral regions between 12 and 21 DIV (Fig. 3l). Coronal section analysis showed that the ventral stream remained detectable at P28, while the dorsal stream was depleted at P28; DCX^+^ cells within streams were absent by 5 months, indicating that populations from the dorsal and ventral Arc were transient (Fig. 3m and Supplementary Fig. 17). These data demonstrate that DCX^+^ migratory streams arise from the Arc target several cortical areas, CC, PirC and TC, in early postnatal gyrencephalic brains.

### Distinct neuronal composition in postnatal migratory streams

We next spatially resolved the molecular profiles of Arc-mediated DCX^+^ migratory streams in the neonatal piglet brain. Immunostaining on sagittal E100 piglet sections showed that SP8^+^DCX^+^ cells that did not express COUP-TFII were abundant in the RMS, as well as in OB, as reported in the mouse and human^36^. COUP-TFII^+^SP8^+^ cells were the most abundant subtype in posterior cortical streams, at 43.5% of the DCX^+^ population (Supplementary Fig. 18). Furthermore, the analysis of the coronal piglet section showed that the dorsal streams into the CC contained a dominant population of LHX6^+^ and COUP-TFII^+^SP8^+^DCX^+^ cells (Supplementary Figs. 19a–d and 20). Consistent with our transcriptomic analysis (Fig. 2), we postulated that Arc supplies LGE-associated neurons into the OB and MGE- and CGE-associated neurons into the cortical regions.

**Fig. 4:**
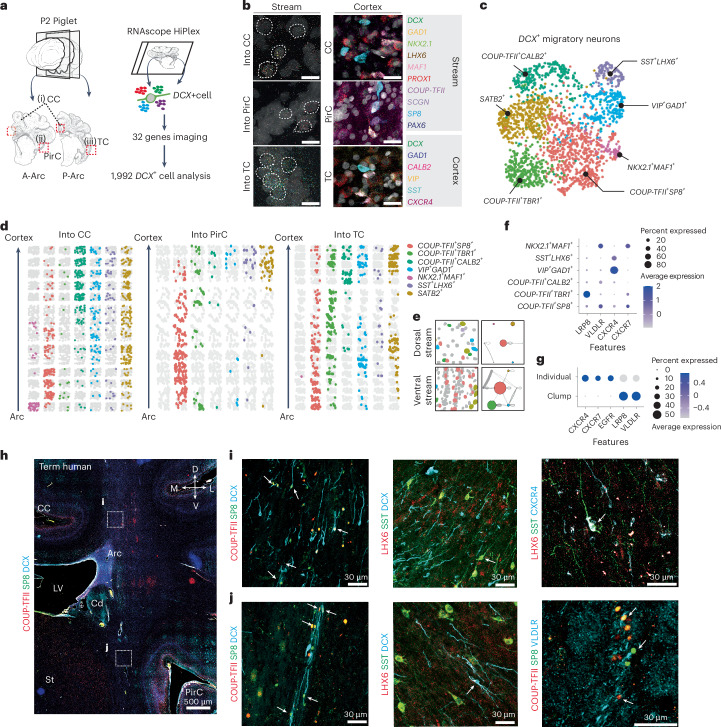
Regionally distinct gene expression patterns for migratory neurons in postnatal cortical streams. **a**, Left, schematic representation indicating the postnatal migratory streams analyzed for profiling cellular subtypes in the P2 piglet brain—dorsal migratory streams into the anterior and posterior CC from the Arc (i), ventral streams into the PirC from the Arc (ii) and ventral streams into the TC from the Arc (iii). Right, schematic representation showing the experimental design for P2 piglet spatial transcriptomics. **b**, Left, smFISH confocal images of each stream showing examples of *DCX*^+^ cells expressing *GAD1*, *NKX2.1*, *LHX6*, *MAF1*, *PROX1*, *COUP-TFII*, *SCGN*, *SP8* and *PAX6*, which are associated with GE. Right, smFISH confocal images of each cortical region showing examples of heterogeneous interneuron types expressing *GAD1*, *CALB2*, *VIP*, *SST* and *CXCR4*. Scale bars, 25 µm. **c**, UMAP projections colored by cell identity. *n* = 1,992 *DCX*^*+*^ cells (see source data for details). **d**, Topographic mapping of migratory neuron subtypes in each stream. From bottom to top shows the migratory stream from the Arc to their final cortical region, that is, CC (left), PirC (middle) and TC (right). **e**, Nearest neighbor analysis in dorsal and ventral migratory streams. The gray line indicates a significant interaction between cell subtypes. The line thickness is correlated with the distance between the cell subtypes. Colored circles represent each cell subtype. A larger circle size indicates a higher number of cells within the cell subtype. The ventral streams show a higher interaction between cell subtypes. **f**, Dot plot illustrating gene expression pattern for receptors for neuronal migration across subtypes. **g**, Dot plot illustrating gene expression pattern for receptors across individual and clump migrating cells. **h**, Coronal images of a human 39 GW showing dorsal and ventral migratory streams of, primarily, COUP-TFII^+^SP8^+^DCX^+^ migratory neurons from the Arc. **i**, White arrows indicate COUP-TFII^+^SP8^+^ clumps in the ventral stream expressing VLDLR. White arrows indicate individually migratory cells expressing LHX6, SST and CXCR4 in the dorsal stream. This experiment has been repeated three times (**h**–**j**). Source data

**Extended Data Fig. 8:**
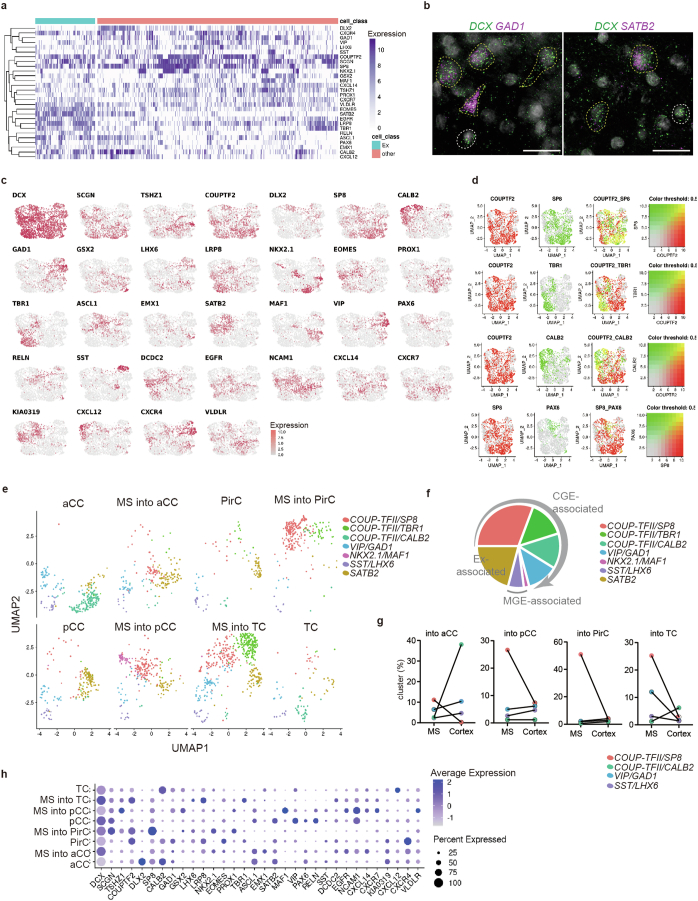
Spatial transcriptomic imagings on P2 piglet brain uncover CGE-associated subpopulations across postnatal cortical streams. **a**, Heatmap depicting expression patterns of major cell-type marker genes across the 1,992 *DCX*^+^ cells analyzed by HiPlex from P2 piglet brain. Migratory neurons cluster into *SATB2*-expressing excitatory (Ex, blue box) and GE marker-expressing inhibitory identities (red box). **b**, Confocal images showing *DCX*^+^*GAD1*^*+*^ migratory neurons (left; yellow dashed line) and *DC*^+^*SATB2*^*+*^ migratory excitatory neurons (right; yellow dashed line). Scale bars, 50 µm. This experiment has been repeated three times. **c**, Feature plots of 32 genes associated with interneuron versus excitatory neuron identity and neuronal migration (Supplementary Table 5). **d**, Feature plots illustrating degrees of gene co-expression. The yellow signal indicates that two genes are co-expressed. *COUP-TFII* is largely co-expressed with *SP8* and only partially with *TBR1* and *CALB2*. The population co-expressing SP8 and PAX6, associated with dorsal lateral ganglionic eminence (dLGE), is small. **e**, UMAP projection by different regions. Each migratory stream (MS) has an abundant *COUP-TFII/SP8* cluster. *COUP-TFII*/*TBR1* cluster is specified in the ventral MS, especially in the temporal cortex (TC). A small population of *NKX2.1/Maf1* is populated in the dorsal stream into the posterior cingulate cortex (pCC). Anterior CC (aCC); piriform cortex (PirC). **f**, Pie chart showing the proportion of clusters grouped by association with ganglionic eminence region. Over 70% of cells express CGE-associated *COUP-TFII*, and 8.2% express MGE-associated *NKX2.1* and *LHX6*. Others express *SATB2*, indicating excitatory neuron identity. **g**, Quantification of cluster proportions in the migratory streams and their targeted cortical regions. The proportion of the *COUP-TFII*/*SP8* cluster decreases along all migratory trajectories. **h**, Dot plot illustrating the expression pattern of selected 32 genes across the different regions.

To further define the molecular profiles of neuroblasts in the postnatal cortex, we performed HiPlex smFISH in P2 piglet brains. We selected *DCX*^+^ cells tracing migratory streams from the Arc into the CC, PirC and TC. We measured gene expression value in 1,992 single *DCX*^+^ cells from the dorsal and ventral streams for 32 genes related to inhibitory identity or migration (Fig. 4a,b). We defined the following six inhibitory subtypes that accounted for 78% of DCX^+^ cells: *NKX2.1*^+^*MAF1*^+^, *SST*^+^*LHX6*^+^, *COUP-TFII*^+^*SP8*^+^, *COUP-TFII*^+^*TBR1*^+^, *COUP-TFII*^+^*CALB2*^+^ and *VIP*^+^*GAD1*^+^. A *SATB2*^+^ (EN) cell type was also identified (Fig. 4c and Extended Data Fig. 8a,b). These subtypes matched those labeled in the transcriptomic dataset of the human Arc (Fig. 2). Over 70% of *DCX*^+^ cells expressed *COUP-TFII*, while 8.2% expressed *NKX2.1* or *LHX6* (Extended Data Fig. 8c,d). Topographic mapping of the *DCX*^+^ profiles across Arc-migratory streams showed that the diverse *COUP-TFII*^*+*^ subtypes populated both migratory streams (Fig. 4d). The *COUP-TFII*^+^*TBR1*^*+*^ cluster was most abundant in the ventral stream into the TC, and the *NKX2.1*^+^*MAF1*^*+*^ cluster was restricted to the dorsal stream into the CC. *COUP-TFII*^+^*CALB2*^+^, *VIP*^+^*GAD1*^+^ and *SST*^+^*LHX6*^*+*^ clusters, expressing markers of mature interneuron subtypes, were more abundant within the cortical regions (Extended Data Fig. 8e–h). Nearest neighbor analysis of cell-subtype interactions revealed that cells in the ventral stream interacted more compared to those in the dorsal stream, consistent with the clump formation among ventral cells. *COUP-TFII*^+^*SP8*^*+*^ and *COUP-TFII*^+^*TBR1*^*+*^ clusters in the ventral stream, which had the highest interaction scores, also had high self-interaction, indicating that they formed homogenous cell clumps (Fig. 4e). Finally, we analyzed the spatial expression of receptors implicated in interneuron migration. *VLDLR* and *LRP8* (also known as *APOER2*) are essential receptors for Reelin, a key extracellular matrix protein for cortical neuronal migration^37^. *VLDLR* was highly expressed in *NKX2.1*^+^/*MAF1*^*+*^ and *COUP-TFII*^+^*SP8*^*+*^ subtypes, whereas *LRP8* was expressed by *COUP-TFII*^+^*TBR1*^*+*^ cells. *CXCR4* and *CXCR7* encode for the CXCL12 receptors and are expressed by MGE- and CGE-derived interneurons^38^. *CXCR4* was expressed by both *SST*^+^*LHX6*^*+*^ and *VIP*^+^*GAD1*^*+*^ clusters, while *CXCR7* was the least expressed, observed in *NKX2.1*^+^*MAF1*^*+*^ and *COUP-TFII*^+^*SP8*^*+*^ clusters (Fig. 4f). Gene expression patterns for these receptors distinguished cells with individual, radial orientation that expressed *CXCR4* or *CXCR7* from those arranged in cell clumps that expressed *LRP8* and *VLDLR* (Fig. 4g and Supplementary Fig. 21).

In the neonatal human brain, COUP-TFII^+^SP8^+^DCX^+^ cells populated both dorsal and ventral streams with individual and collective cellular arrangements, respectively, as observed in the piglet brain (Fig. 4h). LHX6^+^SST^+^DCX^+^ cells were observed in each stream; the representation of the MGE-associated population was over twofold higher in the dorsal migratory streams into the CC than into the ventral cortices; these were further notable in human streams than piglet streams (Supplementary Fig. 19e–g). In both the human and piglet Arc, VLDLR was expressed by COUP-TFII^+^SP8^+^DCX^+^ clumps in the ventral, while individually migrating cells expressed CXCR4 (Fig. 4i,j).

### Regional postnatal change in cortical interneuron composition

**Fig. 5:**
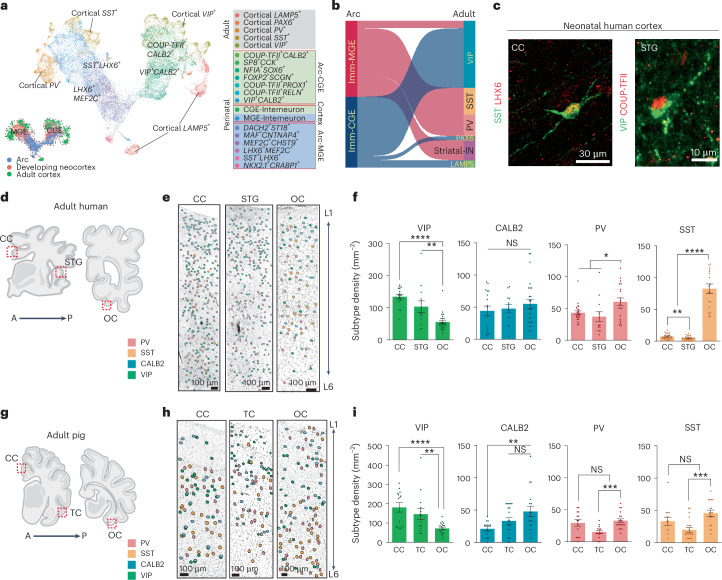
VIP^+^ cortical interneurons are expanded in the adult cingulate and temporal cortex. **a**, The integration of the datasets of human Arc at GW 30–39, human neocortex at GW 17–41 and human adult neocortex and visualization by UMAP. The dotted lines indicate the adult cortical interneuron subtypes. **b**, Sankey diagram illustrating the average fate probabilities of the immature MGE- and CGE-associated interneurons in the Arc. **c**, SST^+^LHX6^+^ neurons in the neonatal human CC WM and VIP^+^COUP-TFII^+^ neurons in the developing human STG. This experiment has been repeated three times. **d**, Schematic representation of the coronal section of the adult human brain, ranging from 15 to 25 years old. Red dot-boxed areas indicate the cortical layers of the CC and STG, a part of the TC. **e**, The distribution of interneuron subtypes within the cortical layers visualized by immunostaining. **f**, The density of each population across the cortex. Two-tailed unpaired *t* test (VIP, **P = 0.0027; ****P < 0.0001. PV, CC vs OC, *P = 0.0128; STG vs OC: *P = 0.0226. SST, **P = 0.0050; ****P < 0.0001). The data are presented as mean ± s.e.m. of counts performed on *n* = 1 cases in five independent experiments. Sample size and *P* values are provided as source data. **g**, Schematic representation of serial coronal sections of the 1-year-old pig brain. Red dot-boxed areas indicate the cortical layers of the CC, TC and OC analyzed in **h** and **i**. **h**, The distribution of the density of each population across cortical layers. **i**, The density of each population across the cortex. Two-tailed unpaired *t* test (VIP, **P = 0.0084; ****P < 0.0001. CALB2, **P = 0.0082. PV and SST, ***P = 0.0001). The data are presented as mean ± s.e.m. of counts performed on *n* = 2 cases in three independent experiments Sample size and *P* values are provided as source data. NS, not significant. Source data

**Extended Data Fig. 9:**
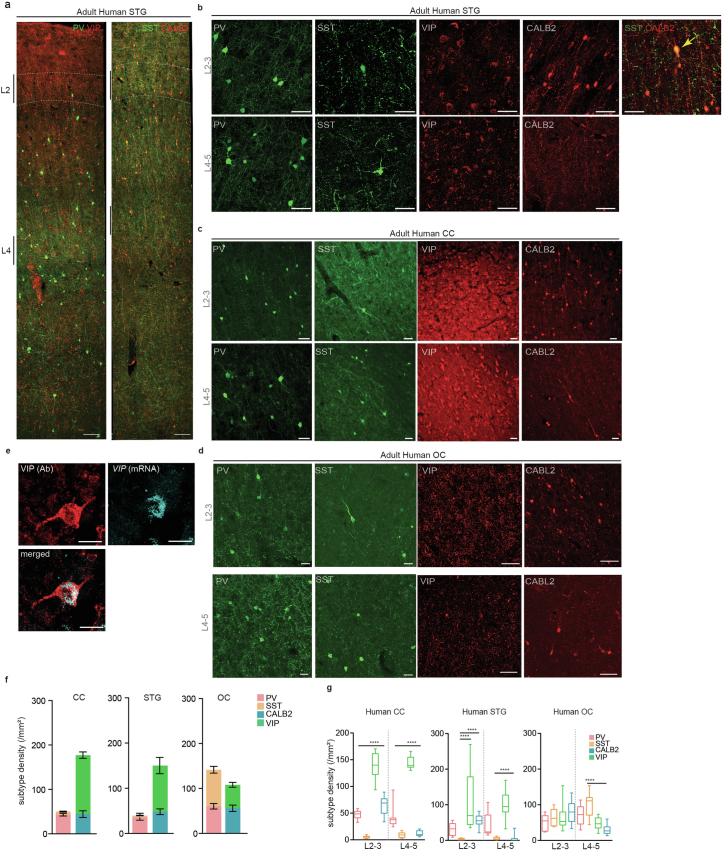
Regional distribution of cortical interneuron subtypes in adult human brain. **a**, The cortical regions of a 25-year-old human temporal cortex (TC). Superior temporal gyrus (STG) is a part of the TC. Left: PV (green)/VIP (red) immunostained neocortical section from the STG. Right: SST (green)/CALB2 (red) immunostained neocortical section from the STG. The white dotted line delineates the border of cell-dense regions of the cortical layer 2. Scale bars, 100 µm. **b**, High magnification images of the 25-year-old human STG immunostained with anti-PV, SST, VIP and CALB2 antibodies. Some interneurons in the adult human STG co-express SST and CALB2 markers. Scale bars, 50 µm. This experiment has been repeated three times. **c**, High magnification images of the 15-year-old human cingulate cortex (CC) labeled with the same antibodies. Scale bars, 50 µm. **d**, High magnification images of the 25-year-old human occipital cortex (OC) labeled with the same antibodies. Scale bars, 50 µm. **e**, Co-detection of protein and mRNA of VIP molecules in the human brain. Scale bars, 25 µm. This experiment has been repeated three times. **f**, The density of each population is measured across cortical layers. Subpopulation expressing VIP is abundant in the Arc-associated cortical regions, including CC and TC, but not OC. The data are presented as mean ± s.e.m. of counts performed on n = 1 case in five independent experiments. **g**, The distribution of the density of each population across cortical layers. Whiskers extend to the minimum and maximum values of the data. Two-tailed unpaired t test (****p < 0.0001). The data are presented as mean ± s.e.m. of counts performed on n = 1 case in five independent experiments. Sample size and *P* values are provided as source data. Source data

**Extended Data Fig. 10:**
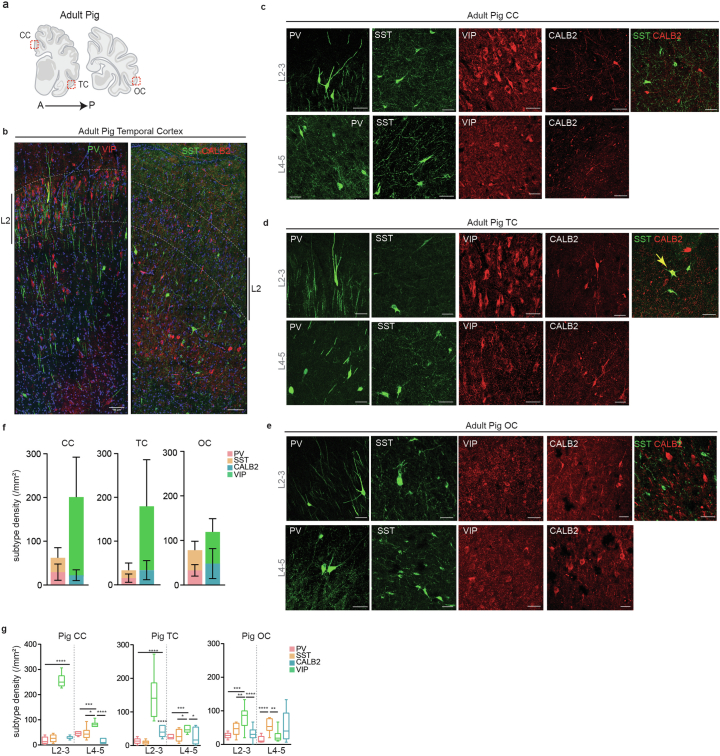
Regional distribution of cortical interneuron subtypes in adult pig brain. **a**, Schematic illustrating anterior and posterior coronal sections of a 1-year-old pig brain. Red boxed areas indicate cingulate cortex (CC), temporal cortex (TC) and occipital cortex (OC). CC and TC are the main destinations of Arc-derived migratory neurons, while OC is less associated with Arc-derived migratory streams. **b**, The cortical regions of the 1-year-old pig brain immunostained for interneuron markers. Left: PV (green)/VIP (red) double-immunostained neocortical section from the temporal cortex (TC). Right: SST (green)/CALB2 (red) double-immunostained neocortical section from the TC. The white dotted line delineates the border of cell-dense regions of the cortical layer 2. Scale bars, 100 µm. This experiment has been repeated three times. **c**–**e**. High magnification images of each cortical area in **a** show that VIP^+^ interneurons are abundant, most notably in cortical layers 2–3 of the cingulate cortex (CC) and temporal cortex (TC). Some interneurons in the adult pig TC co-express SST and CALB2 markers. Scale bars, 50 µm. **f**, The density of each population is measured across cortical layers. Subpopulation expressing VIP is abundant in the Arc-associated cortical regions, including CC and TC, but not OC. The data are presented as mean ± s.e.m. of counts performed on n = 2 cases in three independent experiments. **g**, The distribution of the density of each population across cortical layers. Whiskers extend to the minimum and maximum values of the data. Two-tailed unpaired t test. The data are presented as mean ± s.e.m. of counts performed on n = 2 cases in three independent experiments. Sample size and p values are provided as source data. Source data

To investigate the interneuron subtype composition across Arc–cortical targets, we integrated our snRNA-seq dataset with published snRNA-seq datasets of human developing and adult neocortical interneurons^39^ (Supplementary Fig. 22). Using CellRank to compute cell fate probabilities, MGE-associated cells from the Arc were predicted to differentiate into *PV*^+^ and *SST*^+^ cortical interneurons of the adult cortex and CGE-associated Arc cells into *VIP*^+^ cortical interneurons (Fig. 5a–c). We quantified PV^+^, SST^+^, VIP^+^ and CALB2^+^ subclasses in Arc–cortical targets, CC and superior temporal gyrus (STG), a part of TC, in the adult human brain. We also included the occipital cortex (OC), which has less contribution of Arc cells (Fig. 5d). The density of VIP^+^ neurons in the CC and STG was substantially higher than those in the OC (Fig. 5e,f and Extended Data Fig. 9). A similar pattern was observed in the pig brain at 1 year where VIP^+^ neurons had a higher density in CC and TC than in OC (Fig. 5g–i and Extended Data Fig. 10). Unlike VIP^+^ populations, the densities of PV^+^ and SST^+^ neurons in both human and pig brains were not higher in the Arc–cortical regions. We further examined the dynamics of these subtypes by analyzing the pig cortices at 5 months and 1 year after birth. The density of VIP^+^ neurons increased in both pig CC and TC, which was similarly observed in the human CC (Supplementary Figs. 23 and 24). The density of SST^+^ neurons across all cortical regions decreased, while that of PV^+^ neurons in the pig and human CC slightly increased with age. Postnatal changes in interneuron densities in the pig OC were small, suggesting that the postnatal maturation of each interneuron subtype differs across cortical regions. Analysis of lissencephalic marmoset CC and TC showed a lower density of VIP^+^ neurons at 1 month after birth (Supplementary Fig. 25), as reported in adult marmoset brains^40^. Altogether, Arc-associated cortical regions in human and pig brains have an expansion of the VIP^+^ population.

## Discussion

We demonstrate that the Arc is an elaborate SVZ, containing diverse interneuron subpopulations in neonatal gyrencephalic brains. Dorsal and ventral migratory populations that arise from the Arc are transcriptionally associated with the medial and caudal ganglionic eminences and target areas of the frontal, cingulate and TC. These cortical areas in human and piglet brains show an increase in VIP^+^ neuronal density compared to other regions. Our findings reveal robust and expansive interneuron migration from Arc that could serve as a potential mechanism for regulating function and plasticity across higher cognitive regions in the neonatal gyrencephalic brains.

What influences led to the expanded SVZ in developing gyrencephalic brains? Cortical folding structures begin to develop in the second trimester, and intracortical neuropil outgrowth, one of the driving forces for cortical surface expansion, occurs from the second trimester to the postnatal period in the primate brains^41^. Both these processes precede the emergence of the Arc and could impact its temporal pattern. We also identified a small population of inhibitory IPC cells in the neonatal human Arc that could be another source of Arc neuroblasts; these IPCs were transcriptionally distinct from TACs, a proliferative population in the mammalian SVZ^25^. Whether Arc In-IPC is associated with recently reported pallial RG cells that generate inhibitory neurons^42^ is unknown. Discovering the mechanisms underlying SVZ expansion may provide insight into the evolutionary principles of human brain development.

One notable difference between human and piglet Arc was the proportion of MGE-associated DCX^+^ cells. Our data showed that the human Arc represents approximately 32% of DCX^+^ neuroblasts as MGE, while the piglet Arc had only 9% as MGE cells. This difference was most notable in the dorsal stream connected to the CC. This species distinction could be due to human differences in the maintenance of neurogenesis in the MGE. The human MGE, for example, remains neurogenic until term and could continue to supply late-born cells to cortical or subcortical regions^43^. These findings support the utility of comparative studies to determine the multiple mechanisms that have evolved to expand cortical development.

Our results also provide evidence that the development of non-MGE neuron subtypes scales across species. These subtypes included CGE-associated populations based on higher co-expression of COUP-TFII and SP8 (refs. ^21,22,44^). Experiments on the 5HT3a-GFP mouse showed CGE-derived migratory neurons target several regions, including the dorsal cortex and striatum through P10 (refs. ^45,46^); DCX^+^ neurons in the P20 ferret brain, a species that develops gyri postnatally, express CGE-related markers in small migratory streams to PFC and the occipital lobe^47^. Our data are consistent with previous observations in adult human and macaque brains that COUP-TFII^+^ cortical interneurons have higher proportions than in the mouse cortex^36,48^. The expansion of late migratory COUP-TFII^+^ neurons could contribute to VIP^+^ neurons in the human brain^36,48,49^. Interestingly, recent primate studies showed a higher proportion of VIP^+^ neurons in the PFC^40,50^. VIP^+^ neurons are a key regulator in modulating synchronized neuronal activity and cortical functions such as visual perception^51,52^. Therefore, understanding the expansion of VIP^+^ cortical neuronal populations across species may give insights into the emergence of different higher cognitive abilities.

How do young migratory neurons from the Arc target their final cortical destinations? CXCR4 was expressed in the individually migrating interneurons, predominant in dorsal streams into the CC, and its ligand, *CXCL12*, was highly expressed in the upper layer of the CC; these expressions were also observed in the individually migrating cells near the TC. It suggests that individually migrating interneurons in the early postnatal cortex are regulated by CXCR4-mediated chemokine signaling for their radial migration. In contrast, DCX^+^ cells arranged in clusters, ensheathed by BLBP^+^ or GFAP^+^ fibers, expressed *VLDLR* and/or *LRP8*, which were predominant in ventral streams of the Arc. However, we observed that TC, a main target region for the ventral stream, did not highly express its ligand, Reelin. Considering a mouse study that VLDLR and LRP8 are involved in chain formation of neuroblasts in the RMS independently of Reelin signaling^53^, expression of VLDLR and LRP8 in the ventral stream may support cluster formation of DCX^+^ cells. Thus, Arc neurons use regionally distinct migratory mechanisms.

Disrupted development of GABAergic interneurons has been extensively linked to neurodevelopmental conditions, such as autism spectrum disorder (ASD) and epilepsy^54,55^. Change of interneuron subtype density and abnormal γ oscillation, for example, have been reported in the PFC, anterior CC and TC in patients with ASD. These are areas that receive the influx of Arc neurons. In addition, the perinatal period is a time of increased risk for injuries such as hypoxia–ischemia and brain trauma^56–58^. Understanding neonatal interneuron development across different mammal brains will give insights not only into how the human brain has evolved but also identify the different neurodevelopmental processes that directly contribute to neurodevelopmental conditions. Currently, we are limited by a lack of specific molecular markers for interneurons, especially for the non-MGE subtypes, such as those used in our study. Furthermore, there is a need for more in vivo modeling of larger, gyrencephalic brains to lineage-trace and map postnatal migration. Advances in these areas will be critical for defining the recruitment of Arc-derived neurons to identify postnatal influences on plasticity and vulnerabilities to neuropsychiatric disorders associated with interneuron dysfunction.

## Methods

### Ethics statement

Pediatric tissues obtained from the University of California, San Francisco (UCSF) were collected from autopsy sources through the UCSF Pediatric Neuropathology Research Laboratory (PNRL). Informed consent was obtained from the next of kin for all pediatric samples obtained from the PNRL. Pediatric samples collected through autopsy were de-identified before acquisition and thus exempt from Institutional Review Board (IRB) review. Patients who agreed signed a written consent after receiving information, both written and oral, given by a physician or midwife. They were informed that agreeing to donate would not affect their medical care and that neither the donor nor the clinical team would benefit from the donation. The use of abortion material was reviewed and approved by the UCSF Committee on Human Research. Protocols were approved by the Human Gamete, Embryo and Stem Cell Research Committee (IRB GESCR 10-02693; IRB 20-31968) at UCSF.

### Tissue collection

Thirteen de-identified human specimens had a postmortem interval of fewer than 48 h. Supplementary Table 3 shows the age, gender, number and clinical history of patients in every experiment. Chimpanzee (*Pan troglodytes*) brains at birth were provided by the National Chimpanzee Brain Resource. Sheep (*Ovis aries*) brains at embryonic day (E) 135 were collected in the certified sheep facility at Maastricht University Medical Center according to European and national standards. Postmortem E144, P0 and P30 marmoset (*Callithrix jacchus*) brains were collected immediately after killing for welfare purposes at the University of Cambridge Marmoset Breeding Colony in accordance with the local Animal Welfare and Ethical Review Board. Pig brains (*Sus Scrofa*) from E62, E89, E100, postnatal day (P) 0–2, P16, P28, 5 months and 1 year of age were collected at the Swine Teaching and Research Center at the University of California, Davis. All animal procedures conformed to the requirements of the Animal Welfare Act and were carried out under the Association of Assessment and Accreditation of Laboratory Animal Care International approved conditions with protocols approved before implementation by the Institutional Animal Care and Use Committee (IACUC) at the University of California, Davis (UC Davis IACUC protocol 23682). The P0-aged mice (*Mus Musculus*; C57/BL6) were collected at the UCSF (UCSF IACUC protocols AN192603-01) and were anesthetized on ice for 1–2 min. Supplementary Table 4 shows the age, gender and number of each animal in every experiment. All brains were fixed in 4% paraformaldehyde (PFA) in PBS for 2 days (mice for 1 day) at 4 °C. Cryopreservation was in 30% sucrose in PBS at 4 °C until tissue specimens had sunk to the bottom of the vial. Tissue specimens were cut into coronal or sagittal blocks and frozen in an optimal cutting temperature compound. Blocks were cut at ~30 µm on a cryostat and mounted on glass slides for experimental analysis.

### MRI and imaging processing

The images were acquired on a full-body GE MR950 7T scanner using a 32-ch Nova Medical head coil with a 3D fast spin echo sequence, isotropic 600-micron resolution, time to echo of ~120 ms and repetition time (TR) of 2.5 s and 8 averages. The scan time was approximately 30 min. The distribution of migratory cells was manually labeled using MNI-Display software^59^. We reconstructed the brains from the MRI images as 3D using ITK-SNAP software^60,61^ and NEOCIVET V2.0 pipeline^62^. We used the public open source for a 4.5-year-old Marmoset MRI^63^ and a P0-aged Mouse MRI (USC Laboratory of Neuroimaging). The cortical surfaces were manually segmented, and segmented stacks were reconstructed for 3D visualization.

### snRNA-seq

#### Sampling

Frozen human samples were sectioned (90 µm) in a cryostat. Before nuclei isolation from samples, RNA integrity was measured on the Agilent 2100 Bioanalyser using the RNA Pico Chip Assay. Samples with RNA integrity number (RIN) > 6.6 were used for nuclei extractions. Frozen sections were transferred from tubes in dry ice to ice-cold lysis buffer (0.32 M RNAse-free sucores, 5 mM CaCl_2_, 3 mM Mg(acetate)_2_, 0,1 mM EDTA, 10 mM Tris–HCl (pH 8.0), 1 mM DTT and 0.15 Triton X-100, 0.2 U µl^−1^ RNAse inhibitor in diethyl pyrocarbonate (DEPC)-treated water). The samples were dissociated using a glass Dounce homogenizer and transferred to a separate 30 ml thick-walled polycarbonate ultracentrifuge tube (Beckman Coulter, 355631). Sucrose solution (1.8 M RNAse-free sucrose, 3 mM Mg(acetate)_2_, 1 mM DTT and 10 mM Tris–HCl (pH 8.0) in DEPC-treated water) was added at the bottom of the tube, and homogenates were centrifuged at 107,000*g* for 2.5 h at 4 °C. The supernatant was carefully removed, and the nuclei pellet was incubated in 200–250 µl of DEPC-treated PBS for 20 min on ice. Resuspended pellets were filtered through a 30 µm strainer. Gene expression and barcode libraries were prepared using the Chromium Next GEM Single Cell 3′ Kit v3.1 (10x Genomics) and sequenced in a NovaSeq 6000 system (Illumina) from the Gladstone Institute.

### Preprocessing snRNA-seq data

Raw sequencing reads were generated from Illumina BCL files using bcl2fastq. Reads were aligned to the human genome assembly GRCh38 using CellRanger 7.1.0. The resulting count matrices were then corrected for ambient RNA contamination using the deep learning package CellBender 0.2.2. Correct count matrices were then filtered to remove cells expressing high percentages of mitochondrial and ribosomal RNA and were subsequently run through the DoubletFinder 2.0.3 R package to remove doublets. The filtered data were then normalized and scaled using Seurat 4.9.9.9058 preprocessing functions.

### Integration of human datasets

The dataset of ref. ^25^ and Adult Human Cortical SMART-seq data were previously annotated, and count matrices were subsetted for cells labeled as interneurons. For the dataset of ref. ^22^, cells were preprocessed as described above. The nearest neighbor graph based on the top 30 principal components (PCs) computed from the PC analysis was then used to cluster cells using the Leiden clustering algorithm. Clusters co-expressing *DCX* and *GAD1* were identified and extracted. The extracted cells from all the datasets were then integrated across samples using scVI (Seurat V5) for batch correction with our dataset. The resulting integrated PCs were used to generate Uniform Manifold Approximation and Projection (UMAP) embeddings, and the data were annotated by a combination of existing labels and expression of marker genes.

### Cross-dataset comparison and cell fate inference

The dataset of ref. ^22^ and Arc datasets were preprocessed for high-quality cells and nuclei and batch integrated using canonical correlation analysis. The top 30 dimensions of the resulting low-dimensional embedding were used for constructing a 20-nearest-neighbor graph and identifying clusters using Leiden clustering analysis. The CellRank (2.02) package was used to compute the absorption probabilities of Arc cells into cortical and striatal interneurons^64^. To estimate developmental time, pseudotime was computed using the Monocle3 (1.3.4) package, designating the cycling progenitor cells as the starting root cells. Seurat objects were converted to scanpy (1.9.6) andata (0.10.3) objects to use CellRank. Monocle3 computed pseudotime values were then used in conjunction with transcriptional similarity to model state transitions using a Markov chain^65^.

### Lineage trajectory and gene co-expression network analysis

For the investigation of inferred lineage relationships of interneurons across development, we used the R package monocle3 (1.3.7) to construct a developmental trajectory (nn.k = 40; minimum_branch_len = 35; geodesic_distance_ratio = 0.4). In constructing the individual trajectories, we set the starting point as the progenitor cells taken from the dataset of ref. ^22^. For cortical trajectories, the cells sampled from the cortex in the dataset of ref. ^25^ were set as the endpoint nodes. To compute the pseudotime values for the trajectories, we set the progenitor cells as the root nodes. To reveal gene network level expression patterns, the hdWGCNA package (0.3.01) was implemented. To account for variation within datasets, a consensus approach was used to separately investigate changes across datasets and then identify shared gene expression patterns. Metacells were constructed using *k* = 25 nearest neighbors with a maximum of ten cells shared between two metacells.

### Cresyl staining (Nissl)

Frozen slides were allowed to thaw and equilibrate at room temperature overnight. Slides were baked for 20 min at 60 °C and incubated in cresyl violet solution (Sigma-Aldrich, V5265) for 30 min. Slides were washed with distilled water twice and sequentially incubated in 50%, 70%, 95% and 100% ethanol in distilled water for 1 min each, followed by incubation in xylene solution for 3 min. Slides were mounted and cured overnight and imaged using a Leica Aperio Versa 200 slide scanner microscope (Octopus) or Leica Widefield microscope.

### Immunohistochemistry

Frozen slides were thawed overnight at 4 °C and allowed to equilibrate at room temperature for 3 h. For antigen retrieval, slides were boiled at 95–100 °C in 10 mM sodium citrate buffer (pH 6.0) for 5–10 min and then cooled at room temperature. Samples were permeabilized with 0.05 % Triton X-100 in PBS for 10 min, then incubated in 1% H_2_O_2_ in PBS for 1 h and blocked with Tris-NaCl-blocking buffer (TNB) (0.1 M Tris–HCl (pH 7.5), 0.15 M NaCl and 0.5% blocking reagent from PerkinElmer, FP1012) for 1 h. Slides were incubated with primary antibodies in the TNB overnight at 4 °C, followed by incubation with biotinylated secondary antibodies, diluted 1:250 in TNB solution for 2.5 h at room temperature. Next, slides were incubated with horseradish peroxidase-conjugated streptavidin, diluted 1:200 in TNB for 30 min, before incubation with tyramide-conjugated fluorophores (Akoya), diluted 1:100, in amplification buffer (PerkinElmer) for 5 min. Each dilution is the following: Cy3, Cy5 and FITC are 1:100. Tiled images of entire slides were acquired on a Zeiss Widefield at objective ×10 (numerical aperture; NA 0.45). Additional images were acquired on a Stellaris confocal microscope using ×10 (0.4 NA) and ×40 (1.30 oil) objectives.

### HiPlex FISH

The HiPlex assay (v2., ACD) allows multiplexed detection of up to 32 targets on the same tissue sample. Briefly, sections were baked at 60 °C, fixed in 4% PFA, dehydrated with 50%, 70% and 100% ethanol, exposed to antigen retrieval and incubated with protease III for 20 min at 40 °C. For the first hybridization cycle, 12 target probes were hybridized and amplified together. Because the fluorophores are three (T1–T3—488, 555 and 647), which can be detected together, one cycle consists of four rounds of fluorescence detection. Slides were imaged as specified below. After imaging, coverslips were gently removed after soaking slides in 4× saline sodium citrate (SSC) buffer (diluted in distilled water from 20× SSC stock; Invitrogen, 15557044) for at least 1 h at room temperature. Slides were treated with 10% cleaving solution v2 (ACD) for 15 min at room temperature to cleave off the conjugated fluorophores from the previous round. The second round of conjugation of fluorophores was repeated in the same way. Before the second hybridization of the cycle, slides were incubated with HiPlex Up Reagent (ACD) for 5 min at room temperature and washed with PBS-T. This step was repeated three times. The hybridization of probes, amplification and detection were the same as those of the first cycle. A third hybridization cycle was completed as before. Images were acquired using a Leica Stellaris 8 Tau STED confocal Microscope with a 40× objective (1.30 oil). To obtain the exact locations in each round, several landmarks were labeled on each slide. Images from all rounds were registered using HiPlex image registration software v2 (ACD).

### Organotypic slice cultures and live imaging

The fresh piglet brains at P0-aged were cut in a coronal block in cold artificial cerebrospinal fluid (ACSF), which had been oxygenated for at least 1 h. ACSF contained 125 mM NaCl, 2.5 mM KCl, 1 mM MgCl_2_, 1 mM CaCl_2_ and 1.25 mM NaH_2_PO_4_. Brains were embedded in 3.5% low-melting-point agarose (Thermo Fisher Scientific, BP165-25) and sectioned using a Leica VT1200S vibrating blade microtome as 300 µm slices (speed, 2 mm s^−1^; amplification 0.8 mm). Slices were transferred into Millicell-CM slice culture inserts (Millipore, PICM03050) that were immersed in the modified DMEM/F12 + GlutaMAX-I culture medium (Gibco, 0565018) with 1× N_2_ supplement (Gibco, 17502048), 0.05× B27 supplement (Thermo Fisher Scientific, 17504044), 20 ng ml^−1^ hFGF-2 (Gibco, 13256029), 20 ng ml^−1^ hEGF (Gibco, PHG0311), 20 µg ml^−1^ human insulin (Sigma-Aldrich, I9278), 5 ng ml^−1^ BDNF (Sigma-Aldrich, SRP3014), 10 µM Rock inhibitor Y-27632 (Stemgent, 04-0012) and 100 U ml^−1^ penicillin–streptomycin (Gibco, 15140-122). Tissue slices were microinjected with the virus (Ad-CMV-GFP, 1 × 10^10^ PFU ml^−1^, 1 µl; AAV2-CAG-GFP, 1 × 10^13^ GC ml^−1^, 1 µl; Vector Biolabs) into Arc and were kept at 37 °C with 5 % CO_2_ and 8% O_2_. For time-lapse imaging, the media was changed into the modified Basal Medium Eagle (Gibco, 21010046) with 25% Hanks’ Balanced Salt Solution (Gibco, 14025092), 5% FBS (Gibco, 10437028), 1% N_2_ supplement, 0.66% d-(+)-glucose (Sigma-Aldrich, G7528) and 1% penicillin–streptomycin. Samples were imaged on Leica Stellaris 8 confocal microscope with a ×10 objective (0.4 NA) for 72 h at 25 min intervals under temperature and gas control (37 °C, 5% CO_2_) in an imaging chamber (Okolab). Half of the cultural media was replaced with fresh media every day. For post hoc immunostaining, samples were fixed in 4% PFA, and immunohistochemistry was performed as described above.

### Tissue clarification

Fixed brains were sequentially incubated with tissue clearing solutions A and B (Binaree) in a rotator for 3 days each at 37 °C. Brains were washed with distilled water with constant agitation at 4 °C for 2 h and permeabilized with a permeabilization buffer (0.3% Triton X-100, 10% DMSO and 5% BSA in PBS) for 3 days at 37 °C. The primary antibody (DCX, 1:500) was diluted in a blocking buffer (0.5% Tween 20, 5% DMSO and 5% BSA in PBS) and incubated for 3 days at 37 °C. Brains were washed with PBS-T (0.1% Tween 20) under constant agitation at 4 °C for 3 h, followed by incubation with the secondary antibody (Alexa 488, 1:500) for 3 days at 37 °C. Brains were washed with PBS-T and incubated in Mounting & Storage Solution (refractive index, 1.46) in a shaking incubator at 37 °C for 1 day. Images were acquired using a Light-sheet Microscope (Nikon, AZ-100) and UltraMicroscope Blaze (Miltenyi Biotec).

### Imaging analysis

#### Arc area measurement

From the serial coronal Nissl-stained images of the brains, we measured the Arc area and total brain area. To obtain the Arc area ratio (%), the Arc area was divided by the total brain area on the same slide. Tier 1 was measured as a cell-dense layer near the ventricular wall, and tiers 2–3 were the total Arc area minus the tier 1 area.

### GI

The GI from the animals was calculated using a series of coronal Nissl-stained images. Briefly, a contour connecting all cortical surfaces was drawn, and the length of the line was measured. The length of the entire contour connecting each sulcus and gyri was then divided by the length of the line connecting the cortical surface.

### Quantification of α-SMA area

α-SMA, as a marker for vascular smooth muscle cells, was used to label the vascular regions. Fluorescence images of α-SMA were processed to get the real signals by applying a threshold in Fiji software. The apparent false-positive signals were manually filtered out by eye.

### Arc 3D volumetric measurement

To estimate the volume of the piglet Arc across ages, we used the serial coronal sections of Nissl staining with 750 µm intervals. Arc areas were measured in each section and followed equation (1). The total number of serial sections is *n*, *A* is the Arc area and *h* is the distance between the slides.

1 $${\rm{Estimated}}\,{\rm{volume}}=\mathop{\sum }\limits_{n=2}^{n}\left\{({{{A}}}_{n-1}+{A}_{n}+\sqrt{{A}_{n-1}\times {A}_{n}})\times \frac{h}{3}\right\}.$$

### Quantification of DCX expression intensity within the Arc

Fluorescent images of DCX^+^ were processed to obtain real signals by applying a threshold in Fiji software. The apparent false-positive signals were manually filtered out by eye. The signal intensity of DCX-positive pixels was calculated in a rectangular box spanning from the ventricular wall to tier 3 regions. The boundary between tiers was determined based on the DAPI signals.

### Mapping of DCX^+^ cells across species

Brains were sectioned at regular intervals of 750 µm along the anterior and posterior axes. Slides were stained with anti-DCX primary antibody to visualize DCX^+^ streams across brains and imaged at ×10 on a Zeiss Axiovert 200M Microscope. Neurolucida software (MBF Bioscience) was used for analysis. For each DCX^+^ cell, the soma with the leading neuronal process was marked.

### HiPlex FISH image processing

Regions of interest (ROIs) were carefully chosen within the migratory streams anatomically connected from the Arc. The remaining ROIs were systematically followed away from this ‘site of origin’ to the cortical regions. To quantify transcript abundance from HiPlex FISH images, ROIs were drawn around *DCX*^*+*^ nuclei. Next, images were processed with the Reyni Entropy filter, resulting in a binary mask. The expression value of the transcript was then computed as the pixel percentage area of the probe signal in the mask in reference to the ROI. This process was repeated across all genes in the probe set, and a cell-by-gene expression matrix was generated. After visual analysis, we determined that only cells with greater than the median expression level for a particular gene demonstrated true expression of that gene. These gene expression values below the median were treated as background and set to zero for that cell. The resulting expression matrix was loaded as a Seurat R object. Expression values were log-normalized and scaled before conducting PC analysis. All PCs were used to conduct Leiden neighborhood clustering and produce UMAP embedding.

### Nearest neighbor analysis

The interaction score from the histoCAT neighborhood analysis^66^ was adapted to quantify statistically significant interactions that occurred between cell types. Using the cell-type classifications from the spatial transcriptomic analysis, interactions were quantified by comparing the distances between all cells in each image. Cells with distances less than four pixels were defined as neighbors. The number of pairwise interactions between cells of the same type and different types was quantified and then compared to a distribution generated from the same image with randomized assignments of labels. We then conducted a one-tailed permutation test to produce a *P* value, which represents the likelihood of neighborhood interaction compared to the randomized distribution. To visualize the interactions between cell types, we use the present cell interaction graph. We only visualize interactions if their interaction is significant (*P* < 0.05) in at least 30% of the images and simultaneously present in at least 90% of the images.

### Statistics and reproducibility

Data are analyzed with GraphPad Prism (v.6.0) unless otherwise indicated. The data are presented as mean ± s.e.m., unless otherwise indicated. Distribution of the raw data was tested for normality of distribution; statistical analyses were performed using the Student’s *t* test, two-way analysis of variance with multiple comparison tests as indicated. Images used for quantification were acquired with a total of three replicates (slides) for each animal. Quantifications were performed and repeated by three authors.

### Reporting summary

Further information on research design is available in the Nature Portfolio Reporting Summary linked to this article.

## Online content

Any methods, additional references, Nature Portfolio reporting summaries, source data, extended data, supplementary information, acknowledgements, peer review information; details of author contributions and competing interests; and statements of data and code availability are available at 10.1038/s41593-025-01987-2.

## Supplementary information

Supplementary Information Supplementary Figs. 1–25 and Tables 1–5.

Reporting Summary

Supplementary Data 1 Statistical supporting data for Supplementary Fig. 3.

Supplementary Data 2 Statistical supporting data for Supplementary Fig. 9.

Supplementary Data 3 Statistical supporting data for Supplementary Fig. 12.

Supplementary Data 4 Statistical supporting data for Supplementary Fig. 13.

Supplementary Data 5 Statistical supporting data for Supplementary Fig. 16.

Supplementary Data 6 Statistical supporting data for Supplementary Fig. 19.

Supplementary Data 7 Statistical supporting data for Supplementary Fig. 23.

Supplementary Data 8 Statistical supporting data for Supplementary Fig. 25.

Supplementary Table 1 Comparative structural features across species.

Supplementary Table 2 Comparative Arc features across species.

Supplementary Table 3 Clinical and experimental demographics of collected human specimens.

Supplementary Table 4 Experimental demographics of collected animal specimens.

Supplementary Video 1 Light-sheet imaging of a clarified P0 piglet brain from anterior to posterior. The black signal is DCX immunoreactivity. The stars (*) indicate dorsal streams of DCX^+^ cells from the Arc to the CC and ventral streams of DCX^+^ cells from the Arc to the PirC.

Supplementary Video 2 Three-dimensional rendering of DCX^+^ cells (green) and BLBP^+^ cells (magenta) in dorsal streams from P0 piglet Arc.

Supplementary Video 3 Three-dimensional rendering of DCX^+^ cells (green) and BLBP^+^ cells (magenta) in ventral streams from P0 piglet Arc.

Supplementary Video 4 Time-lapse imaging showing migrating neurons in P0 piglet organotypic slice culture. The area imaged is the dorsal side of the Arc. The cell-dense region is the Arc, and the white line delineates the boundary of the Arc. Note that labeled cells (•) are traveling in a dorsal direction, away from the Arc. The video spans 34 h.

Supplementary Video 5 Time-lapse imaging showing migrating neurons in P0 piglet organotypic slice culture. The area imaged is the dorsal side of the Arc. The cell-dense region is the Arc, and the white line delineates the boundary of the Arc. Note that labeled cells (•) are traveling in a dorsal direction, away from the Arc. The video spans 34 h.

## Source data

Source Data Fig. 1 Statistical source data.

Source Data Fig. 2 snRNA-seq and statistical source data.

Source Data Fig. 4 HiPlex raw data.

Source Data Fig. 5 Statistical source data.

Source Data Extended Data Fig. 1 Statistical source data.

Source Data Extended Data Fig. 2 Statistical source data.

Source Data Extended Data Fig. 3 Statistical source data.

Source Data Extended Data Fig. 6 Statistical source data.

Source Data Extended Data Fig. 9 Statistical source data.

Source Data Extended Data Fig. 10 Statistical source data.

## Data Availability

The data of this study are available on request from the corresponding author. The data described in this study are available via figshare at 10.6084/m9.figshare.25055588 (ref. ^67^). The newly generated sequencing data, alongside count matrices and metadata in this study, have been deposited in the Gene Expression Omnibus under accession GSE255968. The following public transcriptomic datasets were used to support this study: ref. ^25^ data were downloaded from the Gene Expression Omnibus with the accession GSE217511. Ref. ^22^ data were downloaded with the accession GSE135827. Transcriptomic datasets of adult human cortical regions were downloaded from the Adult Human Cortical SMART-seq data at https://portal.brain-map.org/atlases-and-data/rnaseq/human-multiple-cortical-areas-smart-seq. All genomic analyses were performed using the GRCh38 human genome assembly. The following public neuroimaging datasets were used to support this study: the Developing Human Brain Project at https://www.developingconnectome.org/project/, the Marmoset Brain Mapping v3 at https://marmosetbrainmapping.org/, the Neonatal Mouse Brain Atlas at https://www.loni.usc.edu/research/atlases and the NIH Blueprint NHP Atlas at https://www.blueprintnhpatlas.org/. Source data are provided with this paper.

## Code availability

R and Python code for transcriptomic analysis are available at https://github.com/aunoyp/arc.

## References

[1] Ayala, R., Shu, T. & Tsai, L. H. Trekking across the brain: the journey of neuronal migration. *Cell* **128**, 29–43 (2007). 17218253 10.1016/j.cell.2006.12.021

[2] Buchsbaum, I. Y. & Cappello, S. Neuronal migration in the CNS during development and disease: insights from in vivo and in vitro models. *Development* **146**, dev163766 (2019). 30626593 10.1242/dev.163766

[3] Lim, L., Mi, D., Llorca, A. & Marin, O. Development and functional diversification of cortical interneurons. *Neuron* **100**, 294–313 (2018). 30359598 10.1016/j.neuron.2018.10.009 PMC6290988

[4] Morshead, C. M. et al. Neural stem cells in the adult mammalian forebrain: a relatively quiescent subpopulation of subependymal cells. *Neuron* **13**, 1071–1082 (1994). 7946346 10.1016/0896-6273(94)90046-9

[5] Gage, F. H. Mammalian neural stem cells. *Science* **287**, 1433–1438 (2000). 10688783 10.1126/science.287.5457.1433

[6] Alvarez-Buylla, A. & Garcia-Verdugo, J. M. Neurogenesis in adult subventricular zone. *J. Neurosci.* **22**, 629–634 (2002). 11826091 10.1523/JNEUROSCI.22-03-00629.2002 PMC6758521

[7] Altman, J. Autoradiographic and histological studies of postnatal neurogenesis. 3. Dating the time of production and onset of differentiation of cerebellar microneurons in rats. *J. Comp. Neurol.* **136**, 269–293 (1969). 5788129 10.1002/cne.901360303

[8] Doetsch, F. & Alvarez-Buylla, A. Network of tangential pathways for neuronal migration in adult mammalian brain. *Proc. Natl Acad. Sci. USA* **93**, 14895–14900 (1996). 8962152 10.1073/pnas.93.25.14895 PMC26233

[9] Curtis, M. A. et al. Human neuroblasts migrate to the olfactory bulb via a lateral ventricular extension. *Science* **315**, 1243–1249 (2007). 17303719 10.1126/science.1136281

[10] Wang, C. et al. Identification and characterization of neuroblasts in the subventricular zone and rostral migratory stream of the adult human brain. *Cell Res.* **21**, 1534–1550 (2011). 21577236 10.1038/cr.2011.83 PMC3365638

[11] Ponti, G., Aimar, P. & Bonfanti, L. Cellular composition and cytoarchitecture of the rabbit subventricular zone and its extensions in the forebrain. *J. Comp. Neurol.* **498**, 491–507 (2006). 16874818 10.1002/cne.21043

[12] Akter, M. et al. Dynamic changes in the neurogenic potential in the ventricular-subventricular zone of common marmoset during postnatal brain development. *Cereb. Cortex* **30**, 4092–4109 (2020). 32108222 10.1093/cercor/bhaa031

[13] Luzzati, F. et al. Glia-independent chains of neuroblasts through the subcortical parenchyma of the adult rabbit brain. *Proc. Natl Acad. Sci. USA* **100**, 13036–13041 (2003). 14559968 10.1073/pnas.1735482100 PMC240740

[14] Paredes, M. F. et al. Extensive migration of young neurons into the infant human frontal lobe. *Science* **354**, aaf7073 (2016). 27846470 10.1126/science.aaf7073 PMC5436574

[15] Porter, D. D. L. et al. Neuroblast migration along cellular substrates in the developing porcine brain. *Stem Cell Rep.* **17**, 2097–2110 (2022). 10.1016/j.stemcr.2022.07.015 PMC9481921 35985331

[16] Morton, P. D. et al. Abnormal neurogenesis and cortical growth in congenital heart disease. *Sci. Transl. Med.* **9**, eaah7029 (2017). 28123074 10.1126/scitranslmed.aah7029 PMC5467873

[17] Conrad, M. S., Sutton, B. P., Dilger, R. N. & Johnson, R. W. An in vivo three-dimensional magnetic resonance imaging-based averaged brain collection of the neonatal piglet (*Sus scrofa*). *PLoS ONE* **9**, e107650 (2014). 25254955 10.1371/journal.pone.0107650 PMC4177841

[18] Conrad, M. S., Dilger, R. N. & Johnson, R. W. Brain growth of the domestic pig (*Sus scrofa*) from 2 to 24 weeks of age: a longitudinal MRI study. *Dev. Neurosci.* **34**, 291–298 (2012). 22777003 10.1159/000339311 PMC3646377

[19] Habas, P. A. et al. Early folding patterns and asymmetries of the normal human brain detected from in utero MRI. *Cereb. Cortex* **22**, 13–25 (2012). 21571694 10.1093/cercor/bhr053 PMC3236791

[20] Silberberg, S. N. et al. Subpallial enhancer transgenic lines: a data and tool resource to study transcriptional regulation of GABAergic cell fate. *Neuron* **92**, 59–74 (2016). 27710791 10.1016/j.neuron.2016.09.027 PMC5063253

[21] Yu, Y. et al. Interneuron origin and molecular diversity in the human fetal brain. *Nat. Neurosci.* **24**, 1745–1756 (2021). 34737447 10.1038/s41593-021-00940-3

[22] Shi, Y. et al. Mouse and human share conserved transcriptional programs for interneuron development. *Science* **374**, eabj6641 (2021). 34882453 10.1126/science.abj6641 PMC7618238

[23] Sorrells, S. F. et al. Immature excitatory neurons develop during adolescence in the human amygdala. *Nat. Commun.* **10**, 2748 (2019). 31227709 10.1038/s41467-019-10765-1 PMC6588589

[24] Nascimento, M. A. et al. Protracted neuronal recruitment in the temporal lobes of young children. *Nature* **626**, 1056–1065 (2024). 38122823 10.1038/s41586-023-06981-x PMC10901738

[25] Ramos, S. I. et al. An atlas of late prenatal human neurodevelopment resolved by single-nucleus transcriptomics. *Nat. Commun.* **13**, 7671 (2022). 36509746 10.1038/s41467-022-34975-2 PMC9744747

[26] Marcy, G. et al. Single-cell analysis of the postnatal dorsal V-SVZ reveals a role for Bmpr1a signaling in silencing pallial germinal activity. *Sci. Adv.* **9**, eabq7553 (2023). 37146152 10.1126/sciadv.abq7553 PMC10162676

[27] Sawamoto, K. et al. Cellular composition and organization of the subventricular zone and rostral migratory stream in the adult and neonatal common marmoset brain. *J. Comp. Neurol.* **519**, 690–713 (2011). 21246550 10.1002/cne.22543 PMC4096931

[28] Doetsch, F., Garcia-Verdugo, J. M. & Alvarez-Buylla, A. Cellular composition and three-dimensional organization of the subventricular germinal zone in the adult mammalian brain. *J. Neurosci.* **17**, 5046–5061 (1997). 9185542 10.1523/JNEUROSCI.17-13-05046.1997 PMC6573289

[29] Zhao, Z. et al. Evolutionarily conservative and non-conservative regulatory networks during primate interneuron development revealed by single-cell RNA and ATAC sequencing. *Cell Res.* **32**, 425–436 (2022). 35273378 10.1038/s41422-022-00635-9 PMC9061815

[30] Pai, E. L. et al. Maf and Mafb control mouse pallial interneuron fate and maturation through neuropsychiatric disease gene regulation. *eLife* **9**, e54903 (2020). 32452758 10.7554/eLife.54903 PMC7282818

[31] Morabito, S., Reese, F., Rahimzadeh, N., Miyoshi, E. & Swarup, V. hdWGCNA identifies co-expression networks in high-dimensional transcriptomics data. *Cell Rep. Methods* **3**, 100498 (2023). 37426759 10.1016/j.crmeth.2023.100498 PMC10326379

[32] Schmitz, M. T. et al. The development and evolution of inhibitory neurons in primate cerebrum. *Nature* **603**, 871–877 (2022). 35322231 10.1038/s41586-022-04510-w PMC8967711

[33] Sanai, N. et al. Unique astrocyte ribbon in adult human brain contains neural stem cells but lacks chain migration. *Nature* **427**, 740–744 (2004). 14973487 10.1038/nature02301

[34] Sanai, N. et al. Corridors of migrating neurons in the human brain and their decline during infancy. *Nature* **478**, 382–386 (2011). 21964341 10.1038/nature10487 PMC3197903

[35] Gertz, C. C. & Kriegstein, A. R. Neuronal migration dynamics in the developing ferret cortex. *J. Neurosci.* **35**, 14307–14315 (2015). 26490868 10.1523/JNEUROSCI.2198-15.2015 PMC4683689

[36] Ma, T. et al. Subcortical origins of human and monkey neocortical interneurons. *Nat. Neurosci.* **16**, 1588–1597 (2013). 24097041 10.1038/nn.3536

[37] Hack, I. et al. Divergent roles of ApoER2 and Vldlr in the migration of cortical neurons. *Development* **134**, 3883–3891 (2007). 17913789 10.1242/dev.005447

[38] Wang, Y. et al. CXCR4 and CXCR7 have distinct functions in regulating interneuron migration. *Neuron* **69**, 61–76 (2011). 21220099 10.1016/j.neuron.2010.12.005 PMC3025760

[39] Hodge, R. D. et al. Conserved cell types with divergent features in human versus mouse cortex. *Nature* **573**, 61–68 (2019). 31435019 10.1038/s41586-019-1506-7 PMC6919571

[40] Krienen, F. M. et al. A marmoset brain cell census reveals regional specialization of cellular identities. *Sci. Adv.* **9**, eadk3986 (2023). 37824615 10.1126/sciadv.adk3986 PMC10569717

[41] Rash, B. G., Arellano, J. I., Duque, A. & Rakic, P. Role of intracortical neuropil growth in the gyrification of the primate cerebral cortex. *Proc. Natl Acad. Sci. USA* **120**, e2210967120 (2023). 36574666 10.1073/pnas.2210967120 PMC9910595

[42] Delgado, R. N. et al. Individual human cortical progenitors can produce excitatory and inhibitory neurons. *Nature* **601**, 397–403 (2022). 34912114 10.1038/s41586-021-04230-7 PMC8994470

[43] Paredes, M. F. et al. Nests of dividing neuroblasts sustain interneuron production for the developing human brain. *Science* **375**, eabk2346 (2022). 35084970 10.1126/science.abk2346 PMC8887556

[44] Mayer, C. et al. Developmental diversification of cortical inhibitory interneurons. *Nature* **555**, 457–462 (2018). 29513653 10.1038/nature25999 PMC6052457

[45] Inta, D. et al. Neurogenesis and widespread forebrain migration of distinct GABAergic neurons from the postnatal subventricular zone. *Proc. Natl Acad. Sci. USA* **105**, 20994–20999 (2008). 19095802 10.1073/pnas.0807059105 PMC2605417

[46] Touzot, A., Ruiz-Reig, N., Vitalis, T. & Studer, M. Molecular control of two novel migratory paths for CGE-derived interneurons in the developing mouse brain. *Development* **143**, 1753–1765 (2016). 27034423 10.1242/dev.131102

[47] Ellis, J. K. et al. Ferret brain possesses young interneuron collections equivalent to human postnatal migratory streams. *J. Comp. Neurol.* **527**, 2843–2859 (2019). 31050805 10.1002/cne.24711 PMC6773523

[48] Hansen, D. V. et al. Non-epithelial stem cells and cortical interneuron production in the human ganglionic eminences. *Nat. Neurosci.* **16**, 1576–1587 (2013). 24097039 10.1038/nn.3541 PMC4191718

[49] Alzu’bi, A. et al. The transcription factors COUP-TFI and COUP-TFII have distinct roles in arealisation and GABAergic interneuron specification in the early human fetal telencephalon. *Cereb. Cortex* **27**, 4971–4987 (2017). 28922831 10.1093/cercor/bhx185 PMC5903418

[50] Chen, A. et al. Single-cell spatial transcriptome reveals cell-type organization in the macaque cortex. *Cell* **186**, 3726–3743 (2023). 37442136 10.1016/j.cell.2023.06.009

[51] Ferguson, K. A. et al. VIP interneurons regulate cortical size tuning and visual perception. *Cell Rep.* **42**, 113088 (2023). 37682710 10.1016/j.celrep.2023.113088 PMC10618959

[52] Cunha-Reis, D. & Caulino-Rocha, A. VIP modulation of hippocampal synaptic plasticity: a role for VIP receptors as therapeutic targets in cognitive decline and mesial temporal lobe epilepsy. *Front. Cell. Neurosci.* **14**, 153 (2020). 32595454 10.3389/fncel.2020.00153 PMC7303298

[53] Andrade, N. et al. ApoER2/VLDL receptor and Dab1 in the rostral migratory stream function in postnatal neuronal migration independently of Reelin. *Proc. Natl Acad. Sci. USA* **104**, 8508–8513 (2007). 17494763 10.1073/pnas.0611391104 PMC1895980

[54] Sgado, P., Dunleavy, M., Genovesi, S., Provenzano, G. & Bozzi, Y. The role of GABAergic system in neurodevelopmental disorders: a focus on autism and epilepsy. *Int. J. Physiol. Pathophysiol. Pharm.* **3**, 223–235 (2011). PMC3175748 21941613

[55] Contractor, A., Ethell, I. M. & Portera-Cailliau, C. Cortical interneurons in autism. *Nat. Neurosci.* **24**, 1648–1659 (2021). 34848882 10.1038/s41593-021-00967-6 PMC9798607

[56] Alonso-Alconada, D., Gressens, P., Golay, X. & Robertson, N. J. Neurogenesis is reduced at 48 h in the subventricular zone independent of cell death in a piglet model of perinatal hypoxia-ischemia. *Front. Pediatr.* **10**, 793189 (2022). 35573964 10.3389/fped.2022.793189 PMC9106110

[57] Costine, B. A. et al. The subventricular zone in the immature piglet brain: anatomy and exodus of neuroblasts into white matter after traumatic brain injury. *Dev. Neurosci.* **37**, 115–130 (2015). 25678047 10.1159/000369091 PMC4406780

[58] Taylor, S. R. et al. Neuroblast distribution after cortical impact is influenced by white matter injury in the immature gyrencephalic brain. *Front. Neurosci.* **10**, 387 (2016). 27601978 10.3389/fnins.2016.00387 PMC4994423

[59] Wright, R. et al. Automatic quantification of normal cortical folding patterns from fetal brain MRI. *Neuroimage* **91**, 21–32 (2014). 24473102 10.1016/j.neuroimage.2014.01.034

[60] Hughes, E. J. et al. A dedicated neonatal brain imaging system. *Magn. Reson. Med.* **78**, 794–804 (2017). 27643791 10.1002/mrm.26462 PMC5516134

[61] Yushkevich, P. A. et al. User-guided 3D active contour segmentation of anatomical structures: significantly improved efficiency and reliability. *Neuroimage* **31**, 1116–1128 (2006). 16545965 10.1016/j.neuroimage.2006.01.015

[62] Liu, M. et al. Robust cortical thickness morphometry of neonatal brain and systematic evaluation using multi-site MRI datasets. *Front. Neurosci.* **15**, 650082 (2021). 33815050 10.3389/fnins.2021.650082 PMC8010150

[63] Liu, C. et al. Marmoset Brain Mapping V3: population multi-modal standard volumetric and surface-based templates. *Neuroimage* **226**, 117620 (2021). 33307224 10.1016/j.neuroimage.2020.117620 PMC7908070

[64] Weiler, P. et al. CellRank 2: unified fate mapping in multiview single-cell data. *Nat. Methods* **21**, 1196–1205 (2024). 38871986 10.1038/s41592-024-02303-9 PMC11239496

[65] Reuter, B. et al. PyGPCCA—python GPCCA: generalized Perron cluster cluster analysis package to coarse-grain reversible and non-reversible Markov state models. *Zenodo*10.5281/zenodo.6914001 (2022).

[66] Schapiro, D. et al. histoCAT: analysis of cell phenotypes and interactions in multiplex image cytometry data. *Nat. Methods* **14**, 873–876 (2017). 28783155 10.1038/nmeth.4391 PMC5617107

[67] Kim, J. et al. An expanded subventricular zone supports postnatal cortical interneuron migration in gyrencephalic brains. *figshare*10.6084/m9.figshare.25055588 (2025). 10.1038/s41593-025-01987-2 PMC12321571 40659844

