# Supplementary material for: An expanded subventricular zone supports postnatal cortical interneuron migration in gyrencephalic brains
Source: Nat Neurosci. 2025 Jul 14;28(8):1598–609. doi: 10.1038/s41593-025-01987-2 (PMC12321571; doi:10.1038/s41593-025-01987-2)
Supplement: Supplementary file 1 — Supplementary Figs. 1–25 and Tables 1–5. [file 41593_2025_1987_MOESM1_ESM.pdf]

nature neuroscience

Supplementary information https://doi.org/10.1038/s41593-025-01987-2

An expanded subventricular zone supports postnatal cortical interneuron migration in gyrencephalic brains

In the format provided by the authors and unedited

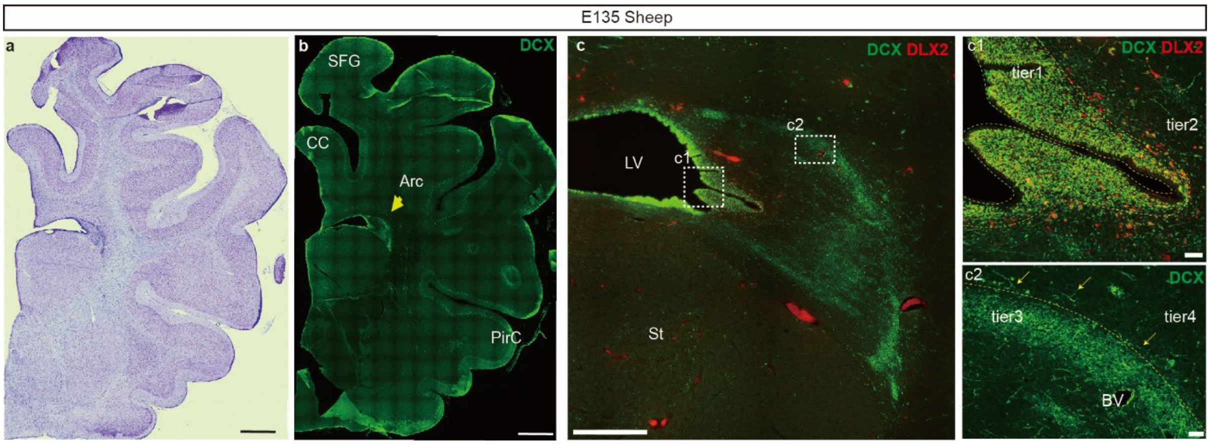

Supplementary Figure 1. Expanded SVZ structure in perinatal sheep brains.

a. Nissl-stained serial coronal brain sections from sheep (*Ovies aries*) brains collected at embryonic day 135 (E135). Sheep are perinatally gyrencephalic and exhibit the cell-dense region near the ventricular wall. The gestational period for sheep is 142 to 152 days, with an average of 147 days. Scale bar, 2mm.

b. Coronal section of the E135 sheep brain, immunostained for DCX. The abundance of DCX+ populations in the ventricular wall (yellow arrow). Scale bar, 2mm. Cingulate cortex (CC); superior frontal gyrus (SFG); and piriform cortex (PirC).

c. High magnifications of the ventricular region show the regionally distinct arrangement of DCX+ cells (in green); dense DCX+ cells along the ventricular wall (tier1), dispersed away (tier2), around blood vessels (tier3), and as clusters oriented towards the pia in the developing white matter (tier4). Higher magnification of the boxed area (c1) shows the dense population of DCX+DLX2+, indicating immature GABAergic inhibitory neurons. The c2 region shows tier2, tier3, and tie4 by distinct distribution of DCX+ cells. Arrows indicate DCX+ cells with elongated, migratory morphology. Scale bar, 500 µm (c) and 30µm (c1 and c2). Lateral ventricle (LV); blood vessel (BV). This experiment has been repeated three times (a-c).

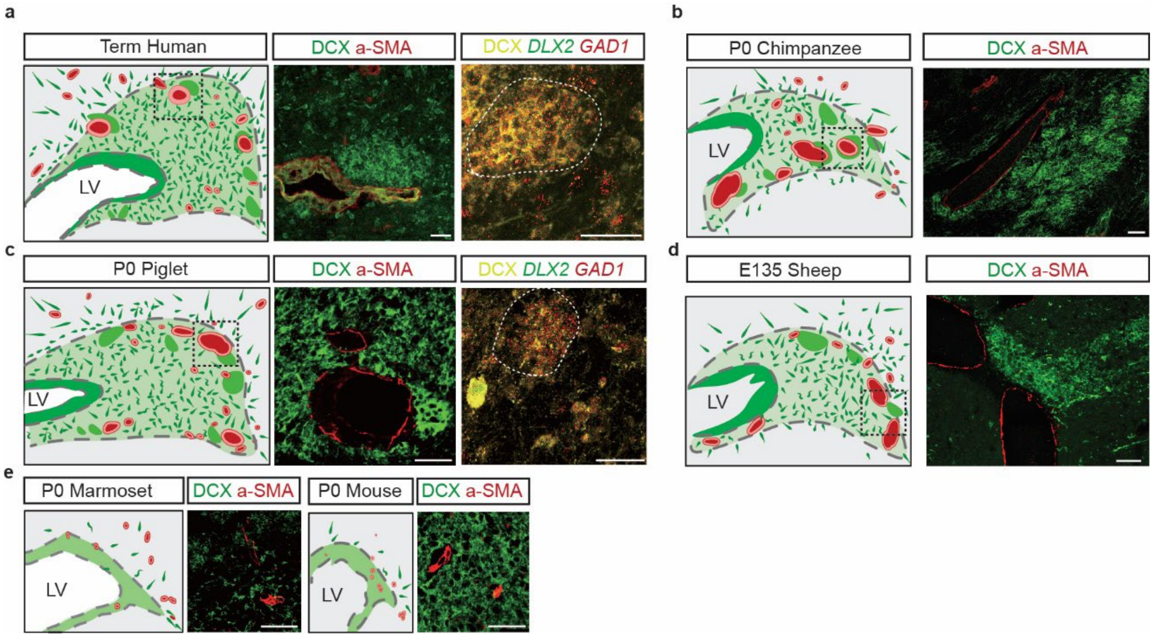

Supplementary Figure 2. Vascular interactions of DCX+ neuroblasts in Arc tier 3.

a-d. Schematics showing the arrangement of DCX+ cells (in green) and distribution of a-SMA+ blood vessels (in red) in the term human Arc (a), P0 Chimpanzee Arc (b), P0 Piglet Arc (c), and E135 sheep Arc (d). High magnification images of the boxed area (shown to the right of the schematics) show interactions of DCX+ clusters with the a-SMA+ blood vessel in tier 3. The cluster of DCX+ cells in contact with the BVs in human and piglet Arc expresses *GAD1* and *DLX2* mRNA. Scale bar, 30µm.

e. Schematics showing DCX+ cells (in green) and a-SMA+ blood vessels (in red) in P0 marmoset and mouse ventricular walls. They do not display dense clusters of DCX+ cells near the BVs. Scale bar, 30µm. Lateral ventricle (LV). This experiment has been repeated three times (a-e).

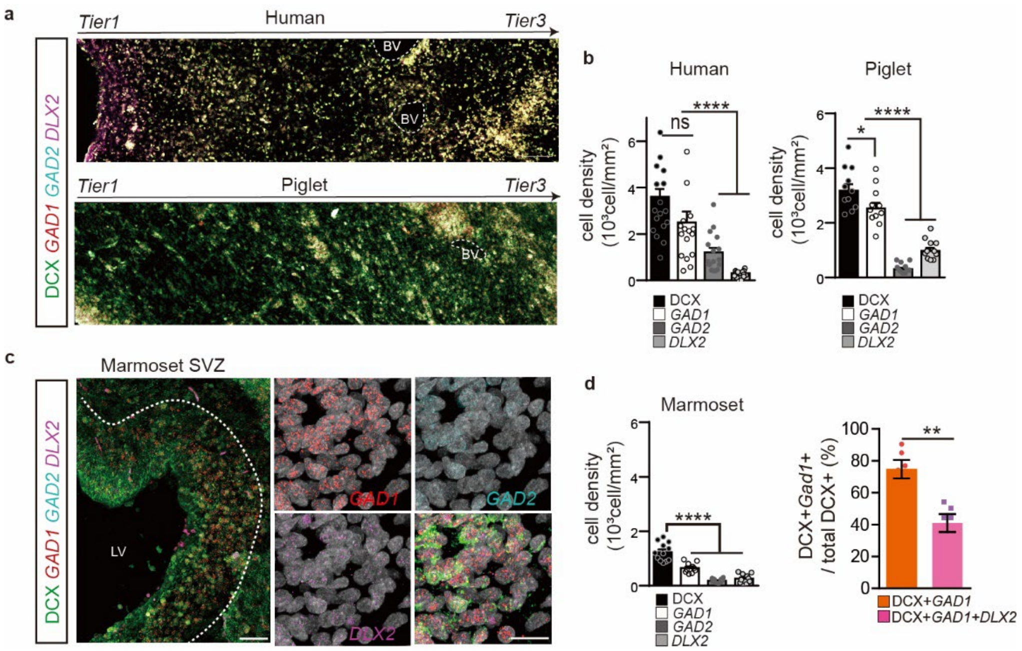

Supplementary Figure 3. Cellular composition at the ventricular wall in the human, piglet, and marmoset brains.

a. Co-detection of mRNAs for GABAergic interneuron markers and DCX protein across the tiered Arc on the neonatal human and P0 piglet brains. Dashed lines indicate blood vessels (BV) in tier 3. Scale bar, 30µm.

b. Quantification of cell densities within the human Arc (left) and piglet Arc (right). Two-tailed unpaired t-test (human, ****, $p < 0.001$; piglet, *$p = 0.0495$, ****, $p < 0.001$). n=2 individuals in three independent experiments (human and piglet). Data are presented as mean ± SEM. Source Data Extended Data Fig.4 shows the sample size.

c. Co-detection of mRNAs for GABAergic interneuron markers and DCX protein in the P0 marmoset ventricular wall. The white dotted line delineates the border of cell-dense regions of the SVZ. Scale bar, 100µm, and 15µm (higher magnification images).

d. Left: Quantification of cell densities and proportion of DCX+*GAD1*+ cells of all DCX+ cells within the ventricular wall. Two-tailed unpaired t-test (****, $p < 0.001$). Right: Quantification of DCX+*GAD1*+ cells and DCX+*GAD1*+*DLX2*+ cells of all DCX+ cells in the marmoset ventricular wall. Two-tailed unpaired t-test (** $p = 0.0019$). Data are presented as mean ± SEM of counts performed on n=2 individual cases in three independent experiments. Source Data Supplementary Fig.3 shows the sample size.

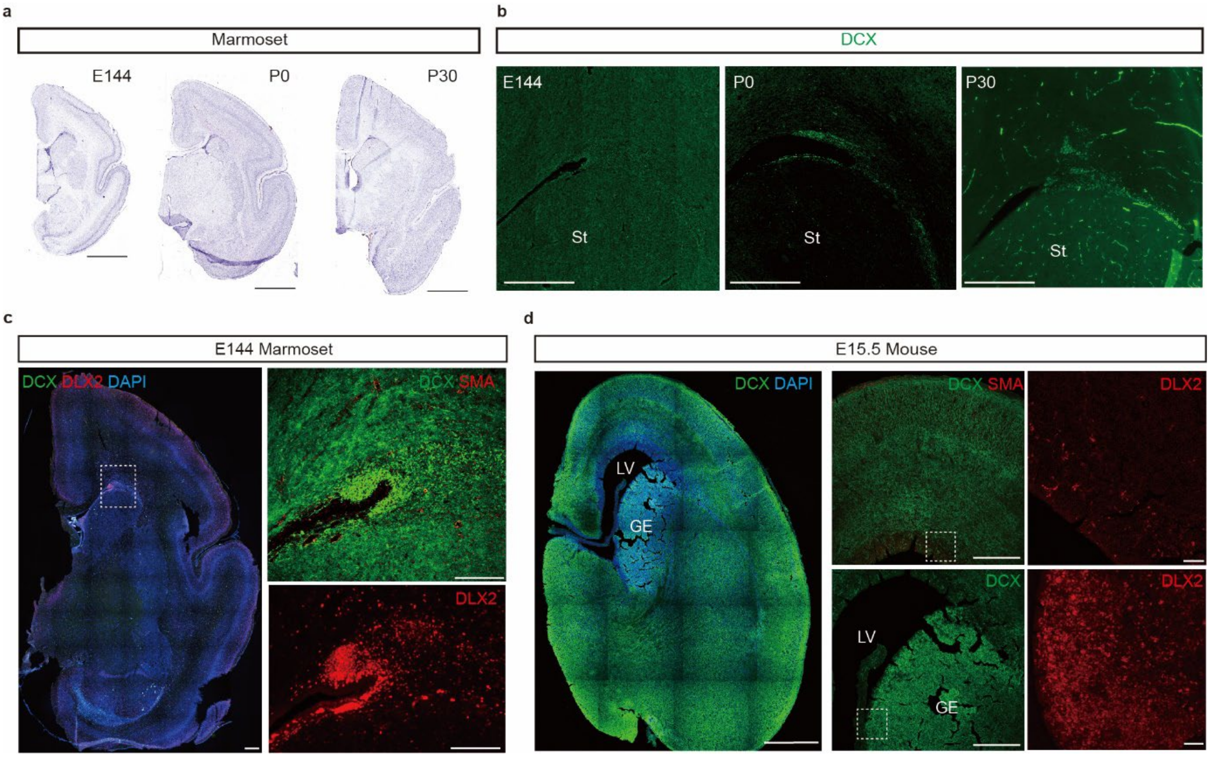

Supplementary Figure 4. Structural changes in the marmoset and mouse SVZ during perinatal stages.

a. Nissl-stain of the coronal section from marmoset brains from embryonic day 144 (E144), postnatal day 0 (P0), to P30. The gestational period of marmoset is 152 days. The cell-dense region remains at the lateral ventricle. Scale bar, 3mm.

b. Confocal images of SVZ structural changes during perinatal stages of the marmosets. The DCX+ neurons (green) populate along the ventricular wall at E144 and P0 but completely disappear at P30. Scale bars, 500 μm. Striatum (St).

c. Wide-field images of an E144 marmoset brain immunostained with DCX (green) and DLX2 (red). Right, top: the higher magnification of the dashed white box shows that the E144 marmoset SVZ does not have a tiered collection of DCX+ cells and a-SMA+ vasculature. Right, down: the higher magnification of the dashed white box shows that the E144 marmoset SVZ contained DLX2+ immature interneurons. Scale bar, 1mm (left), 50 μm (right).

d. Wide-field images of an E15.5 mouse brain immunostained with DCX (green). The cortical ventricular zone at E15.5 does not have the tiered collection by DCX+ cell populations and a-SMA+ vasculature. DLX2+cell is highly populated in the ganglionic eminence (GE). Scale bar, 500 μm. 100 μm (middle), 30 μm (right). This experiment has been repeated three times (a-d). Lateral Ventricle (LV); ganglionic eminence (GE).

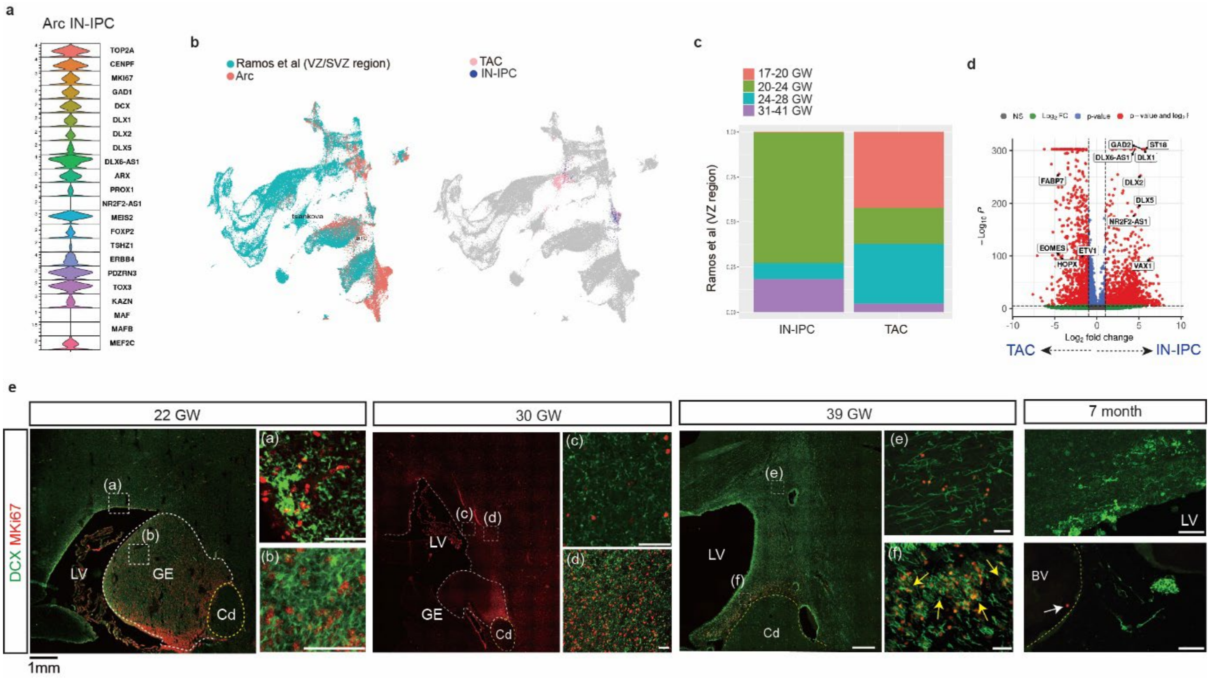

Supplementary Figure 5. Inhibitory-intermediate progenitor cells (In-IPC) in the human Arc.

a. Violin plot of In-IPC marker expression. They express *TOP2A* and *MKI67*, markers of proliferative cells, *PROX1, MEIS2*, and *FOXP2*, markers of CGE/LGE-derived interneurons.
b. Clustering of individual cells (nuclei) from the different datasets[25] visualized by UMAP (left). Transient Amplifying Cell (TAC) and IN-IPC clusters are highlighted.
c. Bar graph showing the contribution of In-IPC and TAC clusters at different stages of development.
d. Volcano plot of differentially expressed genes for TAC and In-IPC. Excitatory-related genes, like *EOMES*, are enriched in TAC clusters, while inhibitory-related genes, like *DLX2*, *DLX5*, and *GAD2*, are enriched in In-IPC clusters. Significance determined by two-sided Wilcoxon rank-sum test Bonferroni adjusted (p-adj. < 0.00001).
e. Confocal images showing regional distribution of Mki67+DCX+ populations in the ventricular regions of humans at 22 GW, 30 GW, 39 GW, and 7 months after birth. Mki67+DCX+ populations are populated in the outer regions within the ganglionic eminence (GE) at 22 and 30 GW. Within the 39 GW Arc, the localization of Mki67+DCX+ cells to ventral regions adjacent to the dorsal (head) of the caudate nucleus (Cd). This proliferative population was depleted within the Arc at 7 months. Scale bars, 1mm (widefield images), 30 µm (high magnified images). This experiment has been repeated three times.

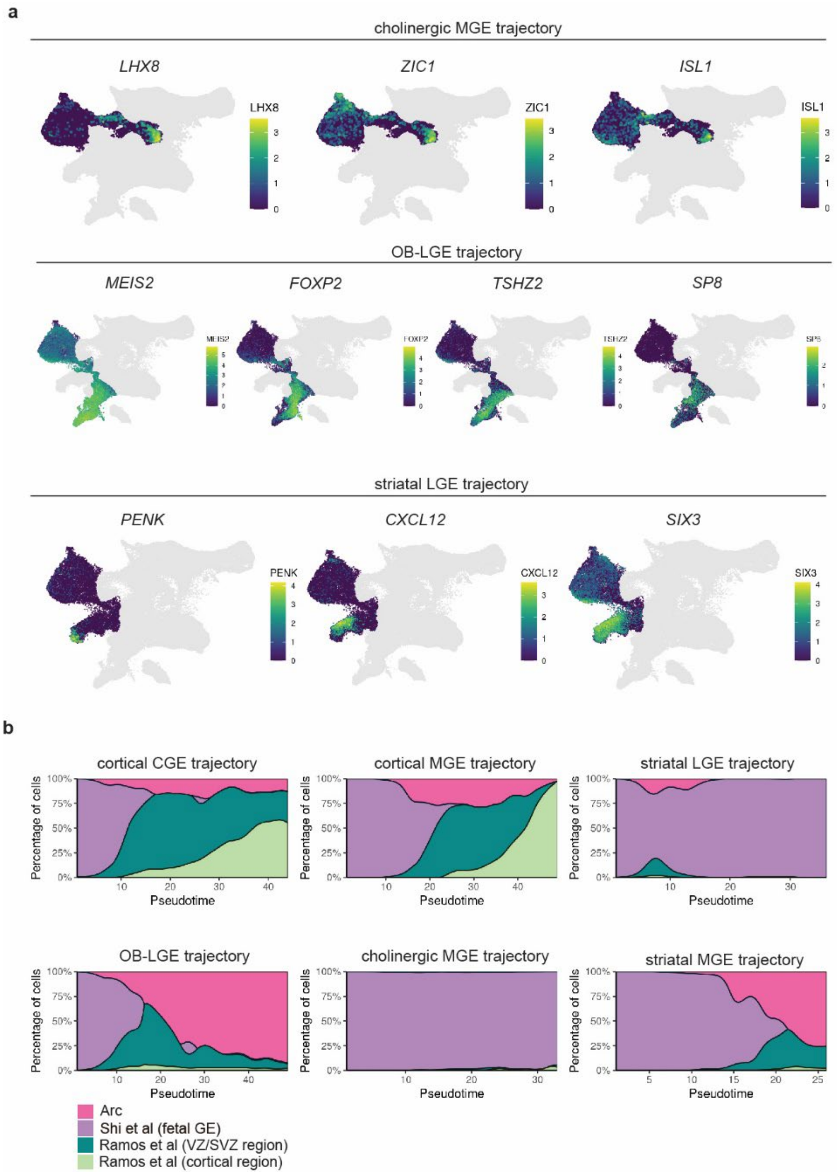

Supplementary Figure 6. The maturation process of human Arc neurons.

a. Main maturation trajectories and their representative gene expressions inferred from Monocle3.

b. Histograms showing the contribution of each dataset to each trajectory along pseudotime. The mature CGE-associated interneurons taken from the developing cortex align in pseudotime with immature CGE-associated interneurons from the Arc and VZ/SVZ, whereas mature MGE-associated interneurons from the cortex are further along in pseudotime compared to Arc MGE-associated interneurons. It suggests that MGE and CGE-related transcriptional programs act differentially with age.

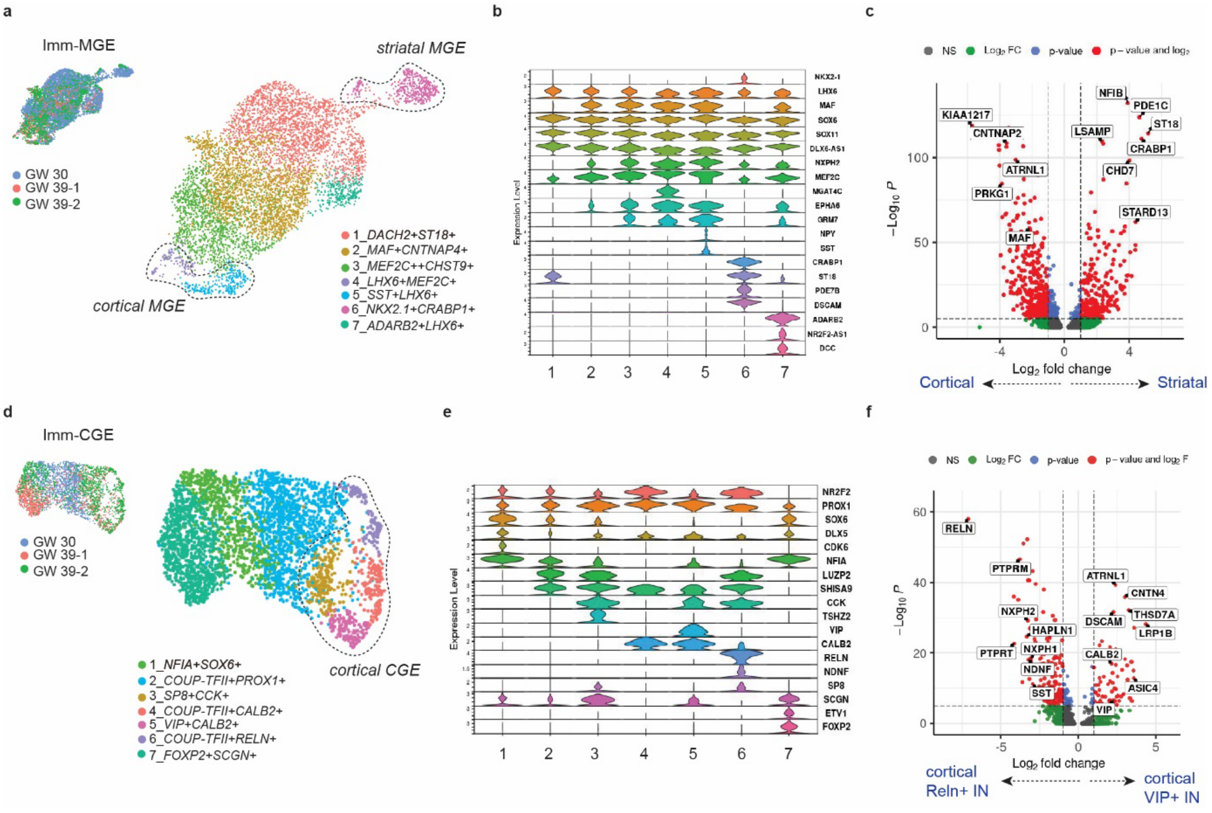

Supplementary Figure 7. Transcriptomic diversity of immature interneurons within the Arc.

a. Cell diversity among MGE-associated interneurons within the human Arc visualized by UMAP. Left: The distribution of immature (Imm) MGE-associated nuclei from three samples. Right: Annotation of 7 subclusters on the basis of gene expression. Cortical MGE subclusters contain nuclei expressing *LHX6* and *MEF2C*, which gives rise to cortical parvalbumin interneurons, and *SST+LHX6+* nuclei, which are committed to cortical somatostatin interneurons. The striatal MGE subcluster contains nuclei expressing *NKX2.1, CRABP1,* and *ST18*, which are characteristic of striatal interneurons.

b. Violin plots of differentially expressed genes among immature MGE clusters.

c. Volcano plot of differentially expressed genes for immature MGE clusters with cortical and striatal potential fates. Cell migration-related genes, like *KIAA1217* and *ATRNL1*, are enriched in nuclei with cortical potential. Wilcoxon rank-sum test Bonferroni-adjusted (p-adj. < 0.05).

d. Cell diversity among CGE-associated interneurons within the human Arc visualized by UMAP. Left: The distribution of immature CGE-associated nuclei from three samples. Right: Annotation of 7 subclusters based on gene expression. Three clusters are committed to cortical CGE-interneurons: *COUP-TFII+CALB2+, VIP*+*CALB2*+, *COUP-TFII+RELN+,* and *SP8+CCK+* cell types. And two immature populations are NFIA+SOX6+ and *COUP-TFII+PROX1+*cell types. Lastly, there is an immature PROX1+ cell state that is also FOXP2+SCGN+, defining a CGE/LGE-like state.

e. Violin plot of differentially expressed genes among immature CGE-related clusters.

f. Volcano plot of differentially expressed genes for immature CGE-associated clusters with cortical *RELN* interneuron and cortical *VIP* interneuron potential fates. Intracellular signaling

pathway-related genes, *PTPRT, NXPH1*, and *NXPH2*, are expressed in nuclei with cortical Reelin interneuron potential, while migration and cytoskeleton-related genes, like *ATRNL1, DSCAM*, and *CNTN4*, are enriched in nuclei with cortical *VIP* interneuron potential. Wilcoxon rank-sum test Bonferroni-adjusted (p-adj. < 0.05).

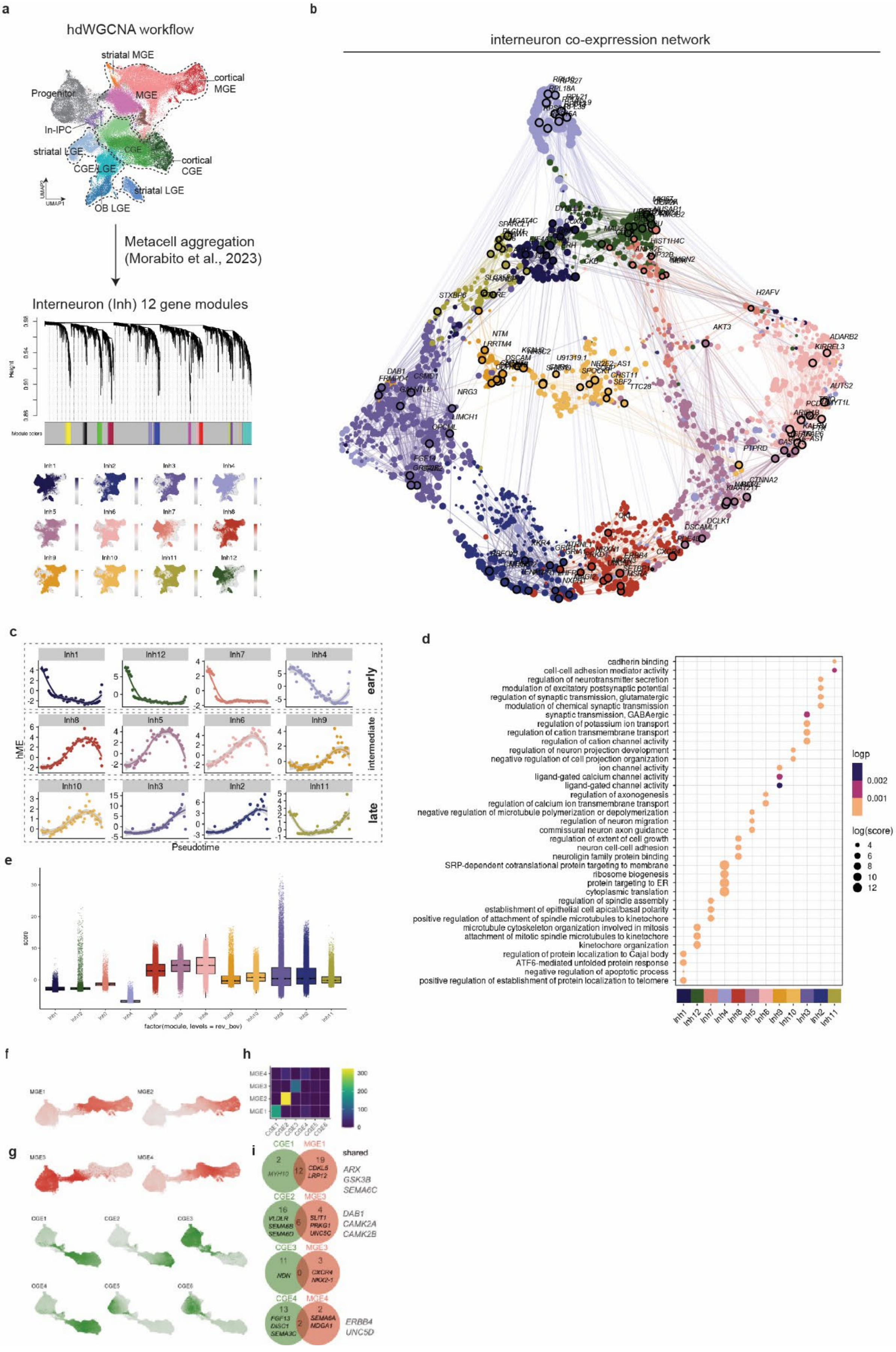
a
hdWGCNA workflow
striatal MGE
cortical MGE
Progenitor
MGE
In-IPC
striatal LGE
CGE
cortical CGE
CGE/LGE
striatal LGE
OB LGE
Metacell aggregation (Morabito et al., 2023)
Interneuron (Inh) 12 gene modules
Height
Module colors
Inh1
Inh2
Inh3
Inh4
Inh5
Inh6
Inh7
Inh8
Inh9
Inh10
Inh11
Inh12
b
interneuron co-exprression network
c
hME
early
intermediate
late
Pseudotime
d
cadherin binding
cell-cell adhesion mediator activity
regulation of neurotransmitter secretion
modulation of excitatory postsynaptic potential
regulation of synaptic transmission, glutamatergic
modulation of chemical synaptic transmission
synaptic transmission, GABAergic
regulation of potassium ion transport
regulation of cation transmembrane transport
regulation of cation channel activity
regulation of neuron projection development
negative regulation of cell projection organization
ion channel activity
ligand-gated calcium channel activity
ligand-gated channel activity
regulation of axonogenesis
regulation of calcium ion transmembrane transport
negative regulation of microtubule polymerization or depolymerization
regulation of neuron migration
commissural neuron axon guidance
regulation of extent of cell growth
neuron cell-cell adhesion
neuroligin family protein binding
SRP-dependent cotranslational protein targeting to membrane
ribosome biogenesis
protein targeting to ER
cytoplasmic translation
regulation of spindle assembly
establishment of epithelial cell apical/basal polarity
positive regulation of attachment of spindle microtubules to kinetochore
microtubule cytoskeleton organization involved in mitosis
attachment of mitotic spindle microtubules to kinetochore
kinetochore organization
regulation of protein localization to Cajal body
ATF6-mediated unfolded protein response
negative regulation of apoptotic process
positive regulation of establishment of protein localization to telomere
logp
0.002
0.001
log(score)
4
6
8
10
12
e
score
factor(module, levels = rev_bov)
f
g
h
i
CGE1 MGE1 shared
MYH10 CDKL5 LRP12
ARX GSK3B SEMA6C
CGE2 MGE3
VLDLR SEMA6B SEMA6D SLIT1 PRKG1 UNC5C
DAB1 CAMK2A CAMK2B
CGE3 MGE3
NDN CXCR4 NKX2-1
CGE4 MGE4
FGF13 DISC1 SEMA3C SEMA6A MDGA1
ERBB4 UNC5D

Supplementary Figure 8. hdWGCNA consensus analysis of interneuron maturation.

a. Schematic overview of the hdWGCNA consensus workflow on the transcriptomic dataset with module dendrogram and feature plots of gene modules [31].

b. UMAP plot of the interneuron co-expression network. Each node indicates a single gene, and edges represent co-expression links between genes and module hub genes. Point size is scaled by eigen-based connectivity (kME). Nodes are colored by co-expression module assignments.

c. Module eigengene values as a function of pseudotime. Eigengene values were averaged over 50 pseudotime bins with a 95% confidence interval LOESS regression line.

d. GO enrichment results for each co-expression module (GO Term enrichment. Fisher's exact test Benjamini-Hochberg adjusted p-value). In particular, module 9 was enriched for GO terms related to calcium ion channel activity (*SCGN*), which are included in the intermediate.

e. Module kME distributions for Arc interneurons. Each point represents the correlation between the gene expression of one of 20,364 Arc interneurons and the first principal component of the gene module. Median, 25th and 75th percentile, and individual kME values are represented by center bar, bottom and top of box, and points respectively.

f-g. Feature plots of gene modules computed for MGE trajectory individually (f) and for CGE trajectory (g).

h. Heatmap representing the number.

i. Venn diagrams for neuronal migration-related genes across modules in CGE and MGE trajectories. *ERBB4* is expressed in both trajectories. *VLDLR* is expressed in the CGE-cortical trajectory, whereas *CXCR4* is in the MGE-cortical trajectory.

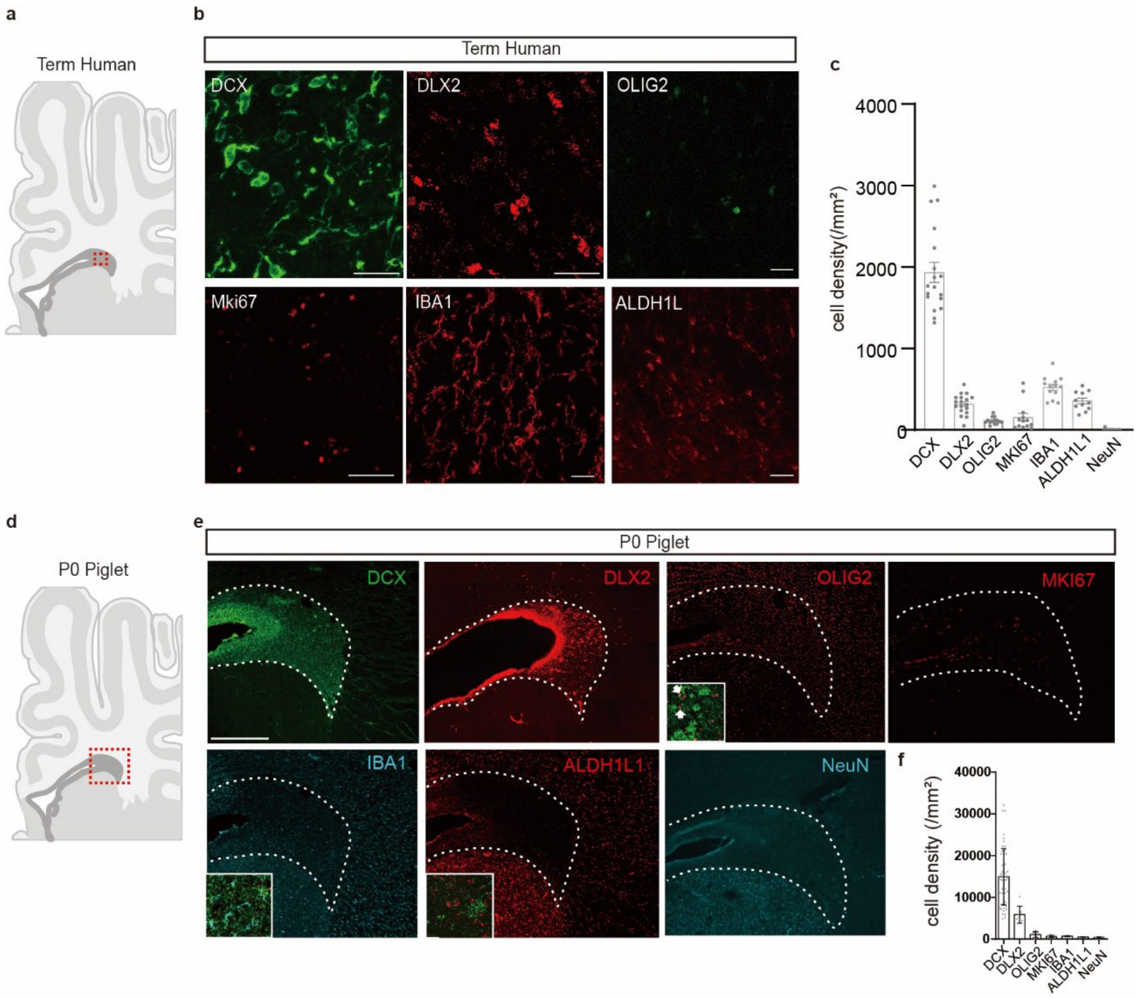

Supplementary Figure 9. Diverse cellular populations in the Term human and P0 piglet Arc.

a. Schematic showing term human Arc region (dashed red box).

b. Confocal imaging showing the diverse cellular types. Immature migratory neurons (marked by DCX; immature GABAergic interneurons (DLX2); oligodendrocytes (OLIG2); proliferative cells (MKI67); microglia (IBA1), as well as astrocytes (ALDH1L1). Scale bar, 30µm.

c. The density of each population is measured within the total Arc area. Cellular composition expressing DCX is abundant in the term human Arc. Data are presented as mean ± SEM counts performed on n=2 cases in three independent experiments. Source Data Supplementary Fig.9 shows the sample size.

d. Schematic showing P0 piglet in Arc region (dashed red box).

e. DCX+ and DLX2+ cells are abundant in the P0 piglet Arc (dashed white line), compared to other cell types, such as oligodendrocytes (marked by OLIG2), microglia (IBA1), astrocytes (ALDH1L1), and mature neuron (NeuN), as well as proliferative cells (MKI67). Scale bar, 500µm.

f. The density of each population is measured within the total Arc area. Cellular population expressing DCX and/or DLX2 is abundant in the P0 piglet Arc. The data are presented as mean

± SEM counts performed on n=2 cases in three independent experiments. Source Data Source Data Supplementary Fig.9 shows the sample size.

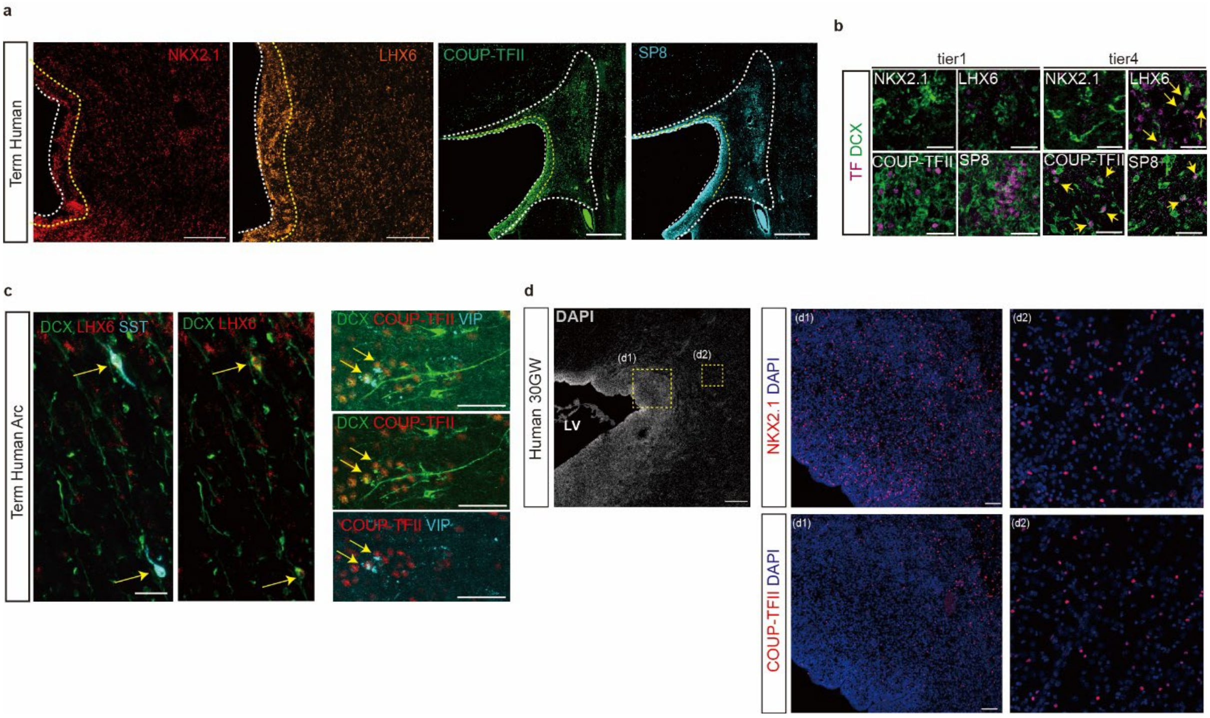

Supplementary Figure 10. Diverse GABAergic neurons within the human Arc.

a. Wide-field images of the neonatal human Arc, immunostained with antibodies against GE-associated transcription factors. The yellow dashed line highlights the boundary between tiers 1 and 2; the white dashed line between tiers 3 and 4 (outer) or between tier 1 and the lateral ventricle (inner). Scale bar, 100µm (images for NKX2.1 and LHX6), 500 µm (images for COUP-TFII and SP8).

b. High magnification images of the human Arc show that DCX+ cells, which express the transcription factors (TF) SP8 and COUP-TFII, are abundant within the Arc, especially in tier 1 and 4. LHX6+DCX+ cells are more abundant in the human tier 4. Yellow arrows indicate DCX+ cells expressing TFs. Scale bar, 20µm.

c. Confocal images showing a subset of DCX+LHX6+SST+ and of DCX+ COUP-TFII+VIP+ within the human Arc. Yellow arrows indicate DCX+ cells co-expressing other markers. Scale bar, 30µm.

d. Left; wide-field image (by DAPI) showing the early Arc at 30 GW. The dashed white boxes (d1 and d2) are immunostained with TFs (NXK2.1 and COUP-TFII) (right). The early Arc at 30 GW was more populated by NKX2.1+ cells than COUP-TFII + cells. Scale bars, 30µm. This experiment has been repeated three times (a-d). Lateral ventricle (LV).

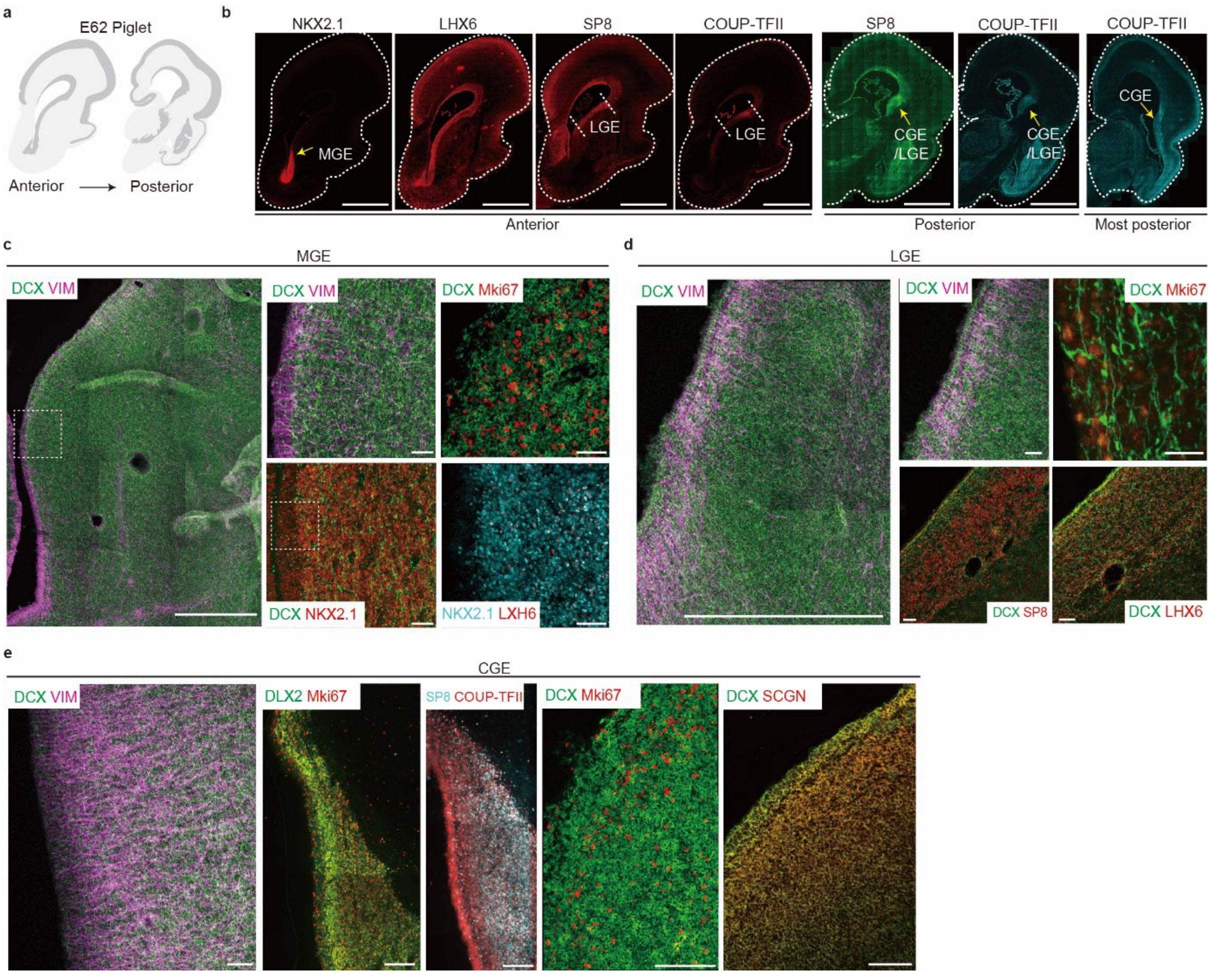

Supplementary Figure 11. GE-associated transcription factors in the E62 piglet brain.

a. Schematic illustrating anterior and posterior coronal sections of an E62 piglet brain at planes shown in (b).

b. Wide-field images of an E62 piglet brain at anterior (left) and posterior (right) planes. Coronal sections were immunostained with antibodies against region-enriched transcription factors. Yellow arrows indicate ganglionic eminence (GE) with increased expression of respective transcription factors. The medial GE (MGE) expresses exclusively NKX2.1, as shown in the anterior sections. The caudal GE (CGE) has a stronger expression of COUP-TFII, as demonstrated from most posterior sections. SP8- and LHX6-expressing cells are observed in the lateral GE (LGE), as shown for an anterior section; LHX6+ cells in the LGE are likely MGE-derived migratory neurons. Both SP and COUP-TFII are expressed in the GEs close to the dorsal lateral ventricle in the posterior section, which is expected to be the CGE/LGE region. Scale bar, 3mm.

c. Immunostaining of NKX2.1, LHX6, Vimentin (VIM), MKI67, and DCX in the E62 piglet MGE. Scale bars, 500 µm (left), 30 µm (higher magnification images).

d. Immunostaining of SP8, LHX6, Vimentin (VIM), MKI67, and DCX in the E62 piglet LGE. Scale bars, 500 µm (left), 30 µm (higher magnification images).

e. Immunostaining of COUP-TFII, SP8, SCGN, Vimentin (VIM), MKI67, and DCX in the E62 piglet CGE. Scale bars, 100 µm. This experiment has been repeated three times (b-e).

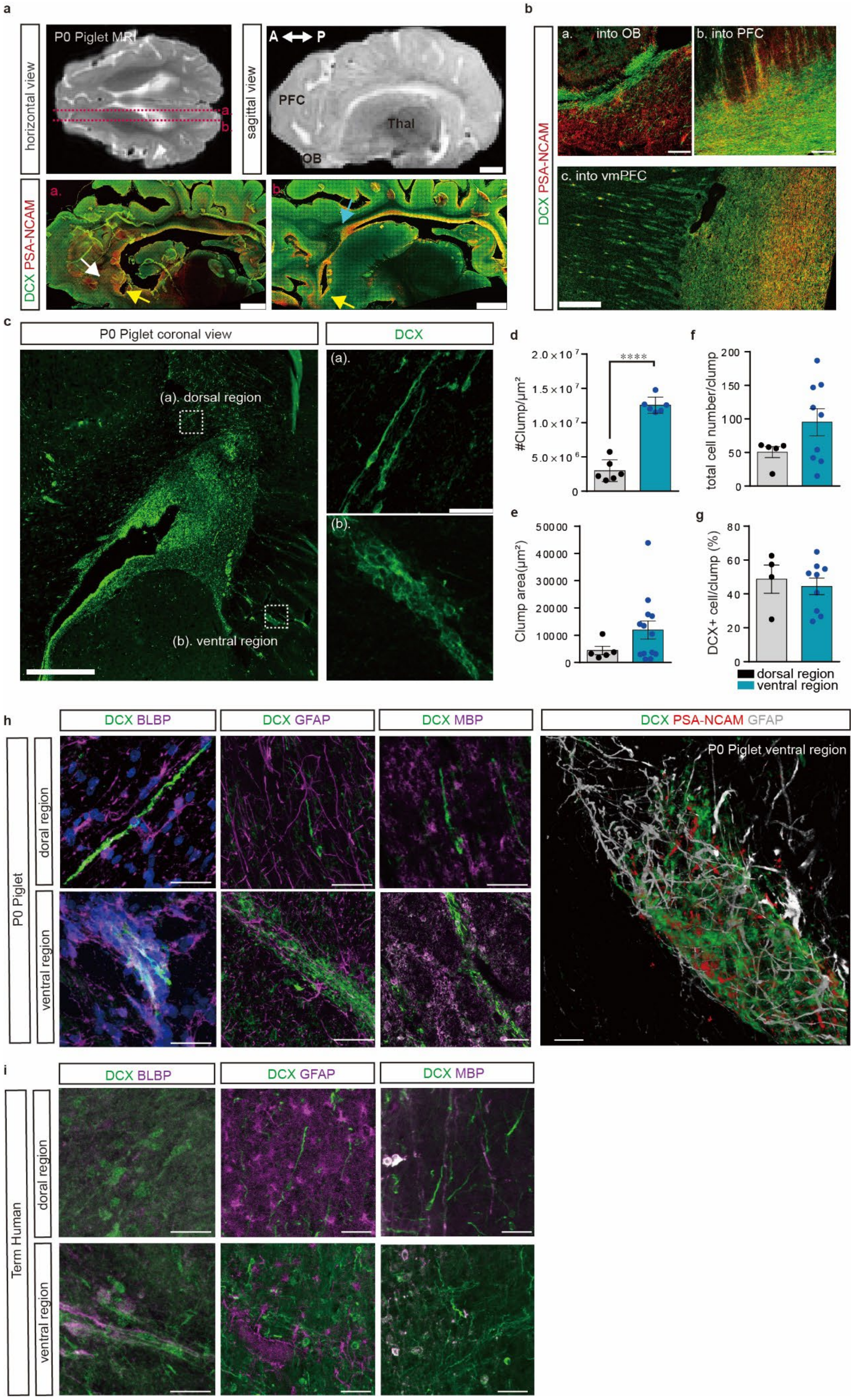
a
horizontal view
P0 Piglet MRI
sagittal view
A ↔ P
PFC
Thal
OB
DCX PSA-NCAM
b
DCX PSA-NCAM
a. into OB
b. into PFC
c. into vmPFC
c
P0 Piglet coronal view
(a). dorsal region
(b). ventral region
DCX
d
#Clump/μm²
****
f
total cell number/clump
e
Clump area(μm²)
g
DCX+ cell/clump (%)
dorsal region
ventral region
h
DCX BLBP
DCX GFAP
DCX MBP
DCX PSA-NCAM GFAP
P0 Piglet
doral region
ventral region
P0 Piglet ventral region
i
DCX BLBP
DCX GFAP
DCX MBP
Term Human
doral region
ventral region

Supplementary Figure 12. Multiple migratory streams emerge from the Arc.

a. Representative T2-weighted MRI images of a P0 Piglet brain (top, left horizontal view; top, right: sagittal view). The bottom panels show the corresponding sagittal sections immunostained with antibodies against DCX and PSA-NCAM. The medial section (a) shows extensive bands from the opened olfactory ventricle (OV, yellow arrow). The lateral section (b) shows the SVZ (Arc) extending into the prefrontal cortex (PFC; blue arrow) as well as OV (yellow arrow). Scale bar, 500µm. Ob; olfactory bulb; Thal: thalamus.

b. Confocal images of a P0 Piglet sagittal section. Migratory neurons expressing DCX (green) and PSA-NCAM (red) located in the RMS (a) targeting the OB, the streams targeting the vmPFC (b), and the streams from the Arc to the PFC (c). Scale bar, 100µm (a) and (c), and 200µm (b).

c. Confocal images of a P0 Piglet coronal section, immunostained with antibodies against DCX. Scale bar, 500µm, and 30µm (higher magnification images).

d to g. Quantification of the clump area (e), the total number of DAPI+ nuclei in a clump (f), and the proportion of DCX+ cells in a clump (g). Two-tailed unpaired t-test, ****p <0.0001 (d). Data are presented as mean ± SEM of counts performed on n=2 cases in three independent experiments. Source Data Supplementary Fig.12 shows the sample size.

h. Confocal images showing the association of DCX+ cells with BLBP+, GFAP+, and MBP+ cells in the dorsal and ventral regions from the P0 piglet Arc. Right: 2D image is a z-projection of the 3D image. Scale bars, 30 µm.

i. Confocal images showing the association of DCX+ cells with BLBP+, GFAP+, and MBP+ cells in the dorsal and ventral regions from the term human Arc. This experiment has been repeated three times (a-c, h, and i). Scale bars, 30 µm.

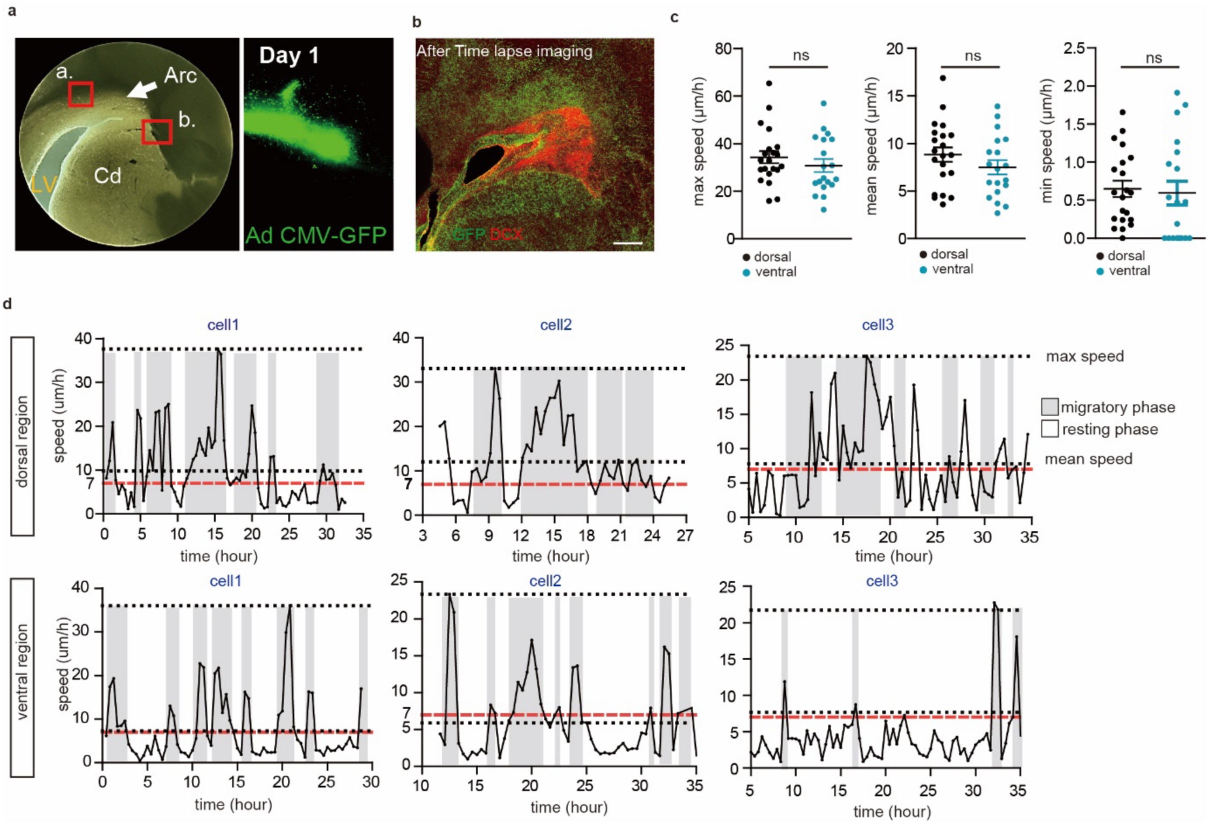

Supplementary Figure 13. Time-lapse imaging of P0 piglet organotypic slice cultures.

a. Left: bright-field images of a P0 piglet organotypic slice containing the Arc (arrow). Boxed areas indicate dorsal (a) and ventral (b) regions analyzed in c and d. Right: GFP+ cells are present in the Arc on day 1 after Adenovirus (Ad) CMV-GFP microinjection into the Arc. Lateral ventricle (LV); caudate nucleus (Cd).

b. Post hoc immunostaining after time-lapse imaging reveals that GFP+ cells within the Arc are mostly DCX+ and migrate from the Arc into dorsal and ventral areas. Scale bar, 100μm.

c. Quantification of the migratory speed of GFP+ cells from the dorsal and ventral regions. No significant difference in maximum, mean, and minimum speeds was observed. Two-tailed unpaired t-test. Data are presented as mean ± SEM of counts performed on n=2 cases in two independent experiments. Source Data Supplementary Fig.13 shows the sample size.

d. Speed profile of cells over time, with a maximum speed (black dashed line, top) that is much higher than the mean speed (black dashed line, bottom). Migratory phases (soma speed higher than 7μm/h; red dashed line) are indicated as a gray box, and resting phases (soma speed less than 7μm/h) as a white box. Three representative cells each from the dorsal and ventral regions are shown. Migrating cells in the ventral regions exhibited more heterogeneity in their migratory behaviors than those in the dorsal regions.

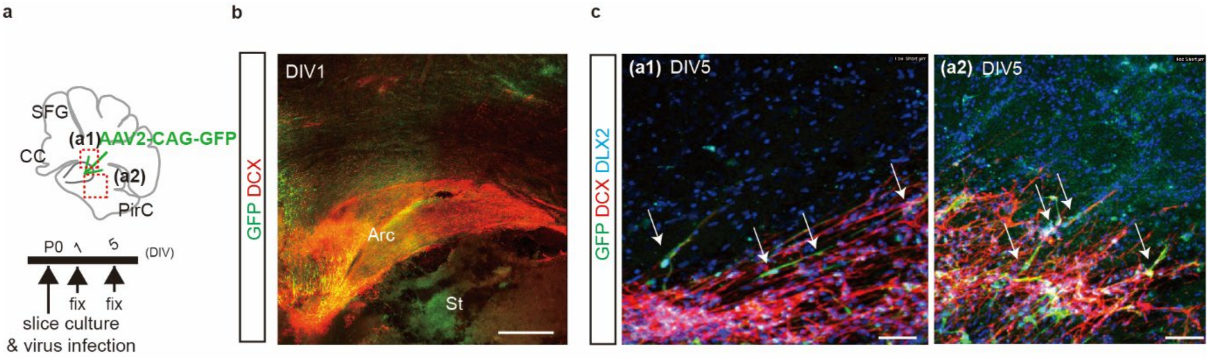

Supplementary Figure 14. P0 piglet organotypic slice cultures with the injection of AAV2-CAG-GFP.

a. Experimental design for extended time-lapse imaging of the P0 piglet brain. Boxed areas (a1) and (a2) adjacent to viral injection sites (AAV2-CAG-GFP) are further illustrated in (b and c).

b. Post hoc immunostaining at DIV1 reveals that GFP+ cells under the CAG promoter within the Arc are mostly DCX+ and migrate out from the Arc into the dorsal and ventral areas. Scale bar, 100µm.

c. Post hoc immunostaining at DIV5 reveals that GFP+DCX+DLX2+ cells migrate from the Arc into the dorsal (a1) and ventral (a2) areas. Scale bar, 30µm. This experiment has been repeated five times (a-c).

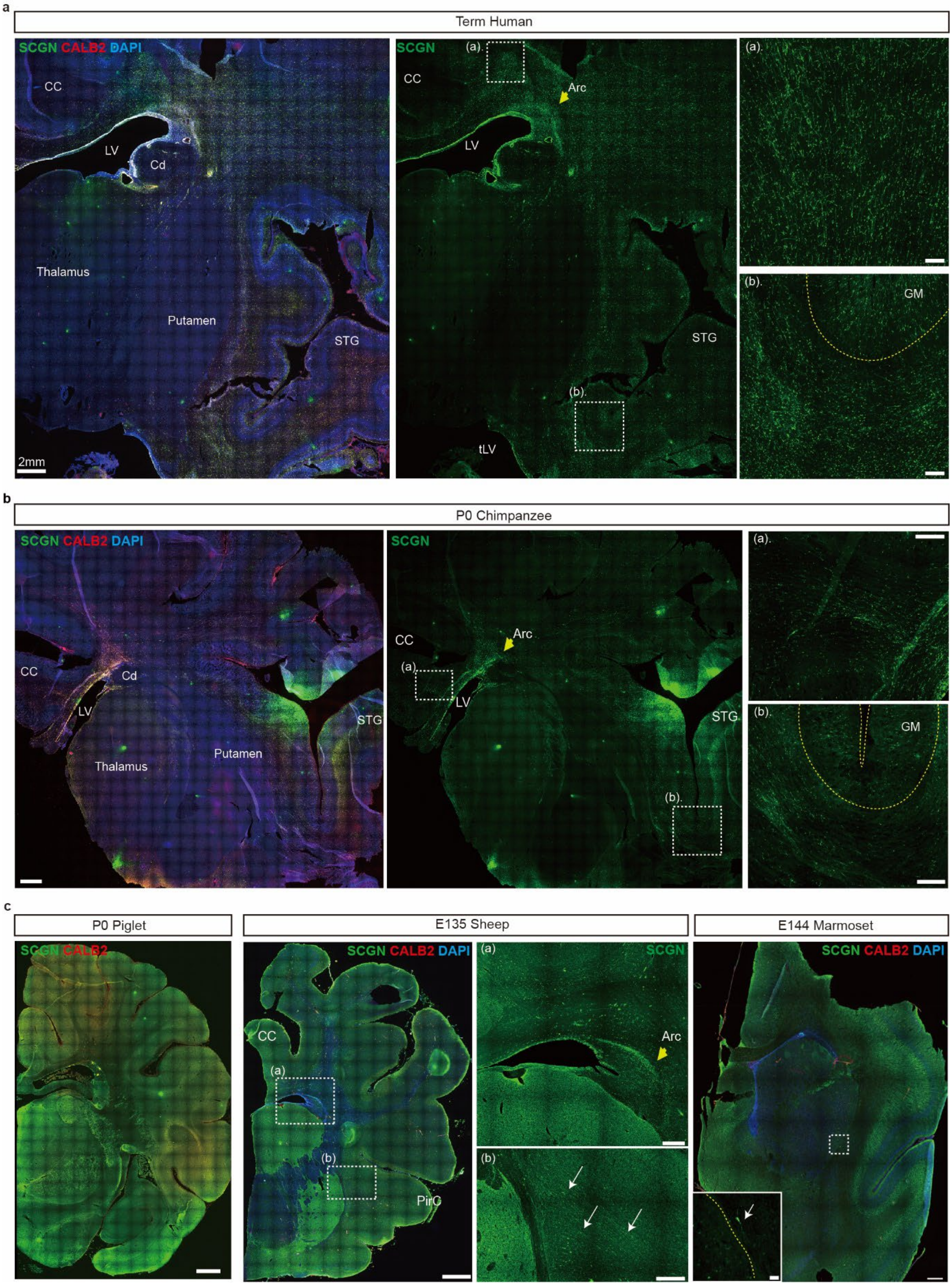
a
Term Human
SCGN CALB2 DAPI
CC
LV
Cd
Thalamus
Putamen
STG
2mm
SCGN
(a).
Arc
tLV
(b).
GM
b
P0 Chimpanzee
SCGN CALB2 DAPI
CC
Cd
LV
Thalamus
Putamen
STG
SCGN
Arc
(a).
(b).
GM
c
P0 Piglet
SCGN CALB2
E135 Sheep
SCGN CALB2 DAPI
CC
(a)
(b)
PirC
SCGN
Arc
E144 Marmoset
SCGN CALB2 DAPI

Supplementary Figure 15. Dorsal and ventral cortical migratory neurons identified by the expression of SCGN in gyrencephalic brains.

a. Wide-field images of the neonatal human brains, at the level of the posterior Arc (P-Arc; refer to Extended Data Fig.6d), immunostained for SCGN, CALB2, and DPAI. Higher magnifications of the boxed areas in (a, middle) show that SCGN+ cells migrate out from the Arc to the cingulate cortex (CC, (a)) and superior temporal gyrus (STG, (b)). Yellow arrow indicates Arc. Scale bar, 2mm (wide-field images), 500 µm ((a) and (b)). Lateral ventricle (LV); Caudate nucleus (Cd); temporal Lateral Ventricle (tLV); Gray matter (GM).

b. Wide-field images of the P0 chimpanzee brains, at the level of the posterior Arc (P-Arc; refer to Extended Data Fig.6d), immunostained for SCGN, CALB2, and DPAI. Higher magnifications of the boxed areas in (a, middle) show that SCGN+ cells migrate out from the Arc to the cingulate cortex (CC, (a)) and superior temporal gyrus (STG, (b)). Yellow arrow indicates Arc. Scale bar, 2mm (wide-field images), 500 µm ((a) and (b)). Lateral ventricle (LV); Caudate nucleus (Cd); temporal Lateral Ventricle (tLV); Gray matter (GM).

c. Wide-field images of the P0 Piglet, at the level of the posterior Arc (P-Arc), the E135 sheep and E144 marmoset brains, at the level of the middle Arc (M-Arc; refer to Extended Data Fig.6d), immunostained for SCGN, CALB2, and DPAI. Higher magnifications of the boxed areas in sheep images show that SCGN+ cells migrate out from the Arc (a) to the cingulate cortex (CC) and pirifrom cortex (PirC, (b)). The yellow arrow indicates Arc, and the white arrows indicate SCGN+ cells with migratory morphology. E144 marmoset brains show rare SCGN+ cells in white matter. Scale bar, 1 mm (wide-field images), 500 µm ((a) and (b)), 30 µm (high magnification images in marmoset). This experiment has been repeated three times (a-c).

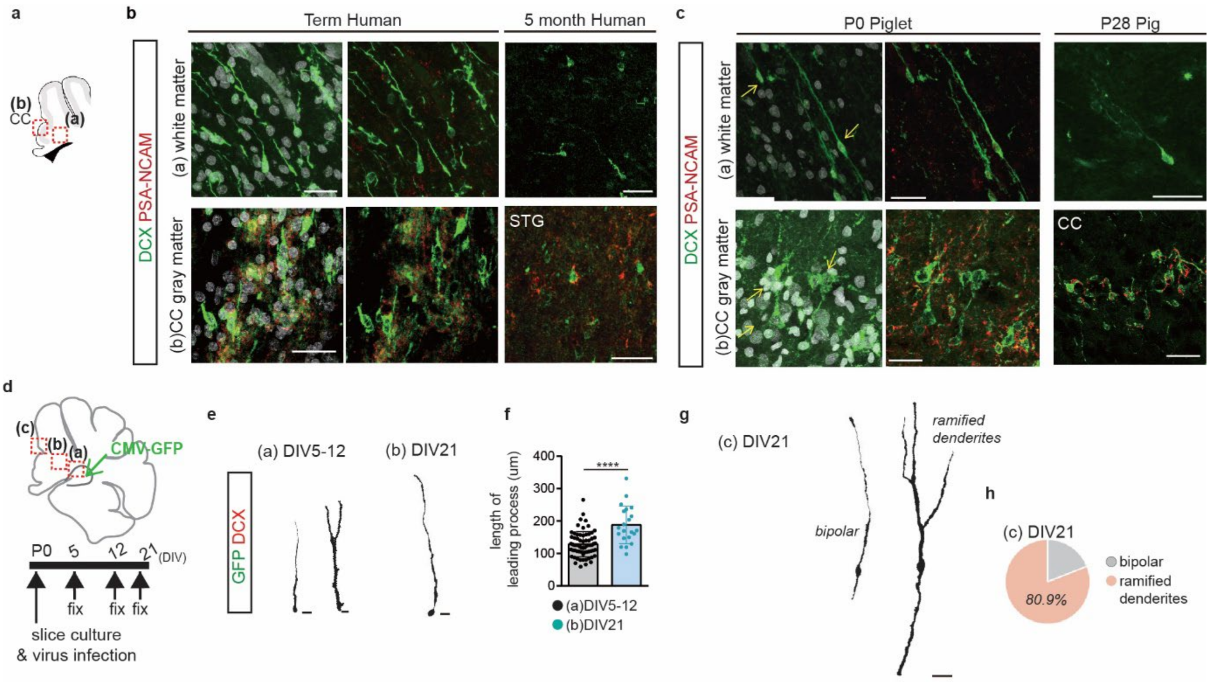

Supplementary Figure 16. Morphological changes of Arc-derived neurons during migration.

a. Schematic showing the white matter close to the Arc ((a)) and the cingulate cortex (CC, ((b)) analyzed in b and c.

b. Confocal images of DCX+PSA-NCAM+ cell population in ((a)) white matter and ((b)) CC gray matter in the term and 5-month humans. The bottom image of 5-months humans shows the DCX+PSA-NCAM+cell population in the gray matter of the superior temporal gyrus (STG). Scale bars, 30 µm.

c. Confocal images of DCX+PSA-NCAM+ cell population in ((a)) white matter and ((b)) CC gray matter in the P0 and P28 piglets. DCX+PSA-NCAM+ cell in the white matter exhibits an elongated nucleus and a long leading process. However, the DCX+PSA-NCAM+ cell in the gray matter in the cortex shows a round nucleus and multipolar morphology. Scale bars, 30 µm. This experiment has been repeated three times (b, c).

d. Experimental design for extended time-lapse imaging of the P0 piglet brain. At DIV0, the Ad-CMV-GFP virus is microinjected into the Arc. The morphology of GFP+DCX+ cells in boxed areas ((a)-(c)) is analyzed at DIV5-21.

e. The representative morphologies of GFP+DCX+ cells in ((a)) region at DIV 5-12 and ((b) regions at DIV 21. Scale bars, 10 µm.

f. Quantification of length of leading process of GFP+DCX+ cells in ((a) regions at DIV 5-12 and ((b)) regions at DIV 21. Two-tailed unpaired t-test (****, $p < 0.0001$). The Data are presented as mean ± SEM of counts performed on n=3 cases in three independent experiments. Source Data Supplementary Fig.16 shows the sample size.

g. Left: the representative morphologies of GFP+DCX+ cells in ((c)) region at DIV 21. Scale bars, 10 µm.

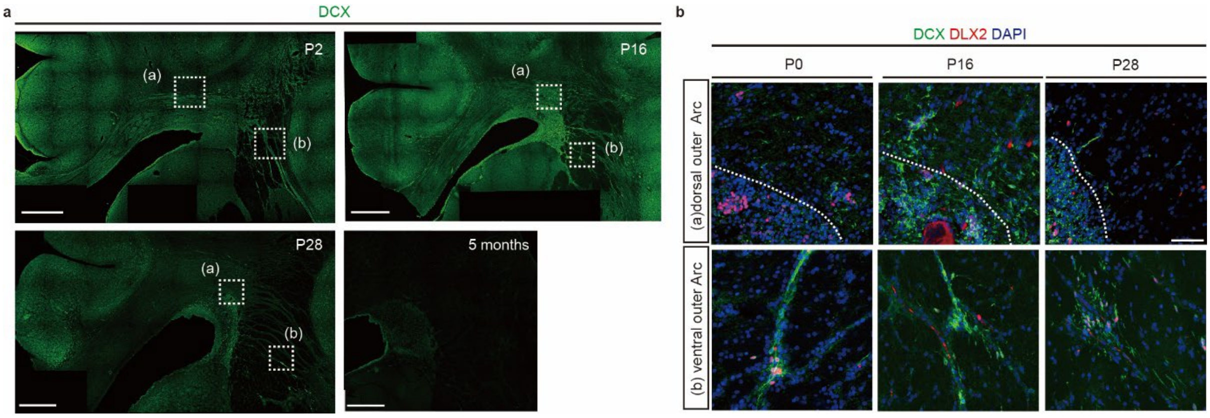

Supplementary Figure 17. Developmental timing of dorsal and ventral migratory streams from the Arc.

a. Wide-field images showing the distribution of DCX+ cells across postnatal stages of the pig. Dorsal streams are detectable until P16, and ventral streams are observed until P28. All streams disappear by 5 months after birth. Higher magnifications of the boxed areas were analyzed in (**b**). Dorsal outer Arc region (a) and ventral outer Arc region (b). Scale bar, 500µm.

b. Confocal images of DCX+DLX2+ cells in the dorsal and ventral outer Arc of P0, P16, and P28 piglet brains. Migration of DCX+ cells from the dorsal outer Arc peaks at P16 and decreases thereafter. The White dashed line indicates a boundary between the Arc and the dorsal outer Arc. Migration of DCX+ cells from the ventral outer Arc persists until P28. Scale bar, 50µm. This experiment has been repeated three times (a, b).

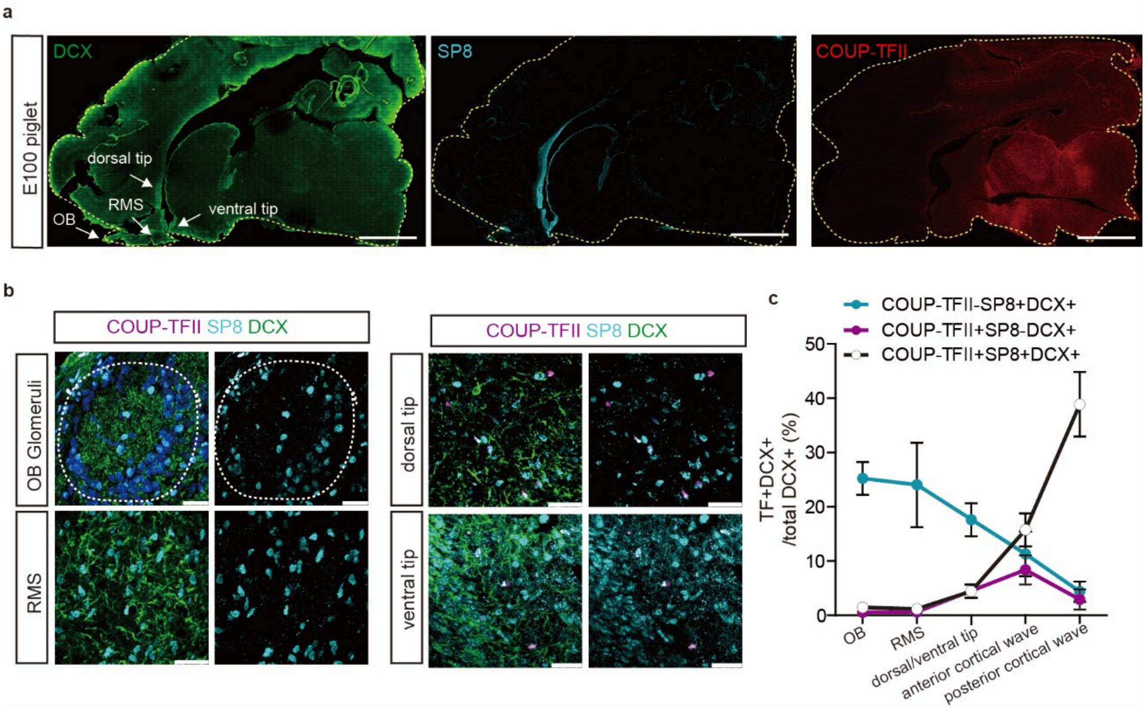

Supplementary Figure 18. COUP-TFII+SP8+ migratory neurons are abundant in the cortical streams, while COUP-TFII-SP8+ migratory neurons are in the rostral migratory stream in the piglet brain.

a. Sagittal sections of E100 piglet brain immunostained with antibodies against DCX, SP8, and COUP-TFII. SP8 abundantly expresses along the anterior ventricular wall connected to the rostral migratory stream (RMS), but COUP-TFII further expresses in the posterior ventricular wall and the ventral brain regions. Scale bars, 500μm. Olfactory bulb (OB). This experiment has been repeated three times.

b. Confocal images from the P0 piglet brain; coronal sections were immunostained for COUP-TFII, SP8, and DCX expression. The rostral migratory stream (RMS) and olfactory bulb (OB) glomeruli (delineated with a white dashed line) are populated by COUP-TFII-SP8+ DCX+ cells, as has been previously reported (*22*, *26*). COUP-TFII+SP8+ and COUP-TFII+SP8-DCX+ cells are rare in the dorsal and ventral tips extending from the olfactory ventricle. Scale bar, 50μm.

c. Quantification of subpopulations of DCX+ cells, differentiated by regional transcription factor (TF) expression, across anterior-posterior migratory streams in the P0 piglet brain. COUP-TFII-SP8+ DCX+ cells are more abundant in anterior migratory streams, like the RMS, whereas COUP-TFII+SP8+ DCX+ cells are found predominantly in posterior cortical streams. The data are presented as mean ± SEM of counts performed on n=3 cases in three independent experiments.

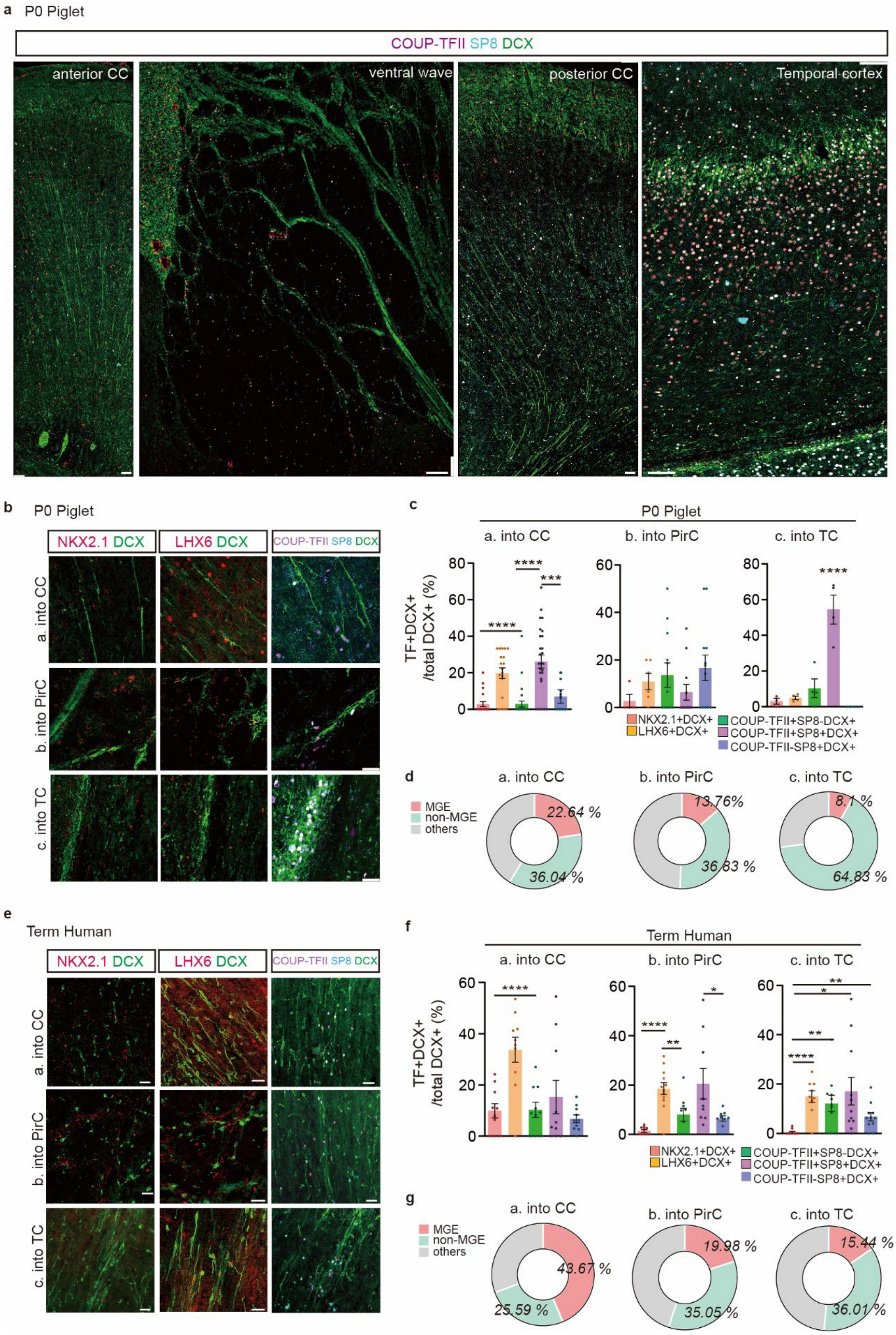
a P0 Piglet
COUP-TFII SP8 DCX
anterior CC
ventral wave
posterior CC
Temporal cortex
b P0 Piglet
NKX2.1 DCX
LHX6 DCX
COUP-TFII SP8 DCX
a. into CC
b. into PirC
c. into TC
c P0 Piglet
TF+DCX+ /total DCX+ (%)
NKX2.1+DCX+
LHX6+DCX+
COUP-TFII+SP8-DCX+
COUP-TFII+SP8+DCX+
COUP-TFII-SP8+DCX+
d
MGE
non-MGE
others
22.64 %
36.04 %
13.76%
36.83 %
8.1 %
64.83 %
e Term Human
f Term Human
g
43.67 %
25.59 %
19.98 %
35.05 %
15.44 %
36.01 %

Supplementary Figure 19. An abundant COUP-TFII+SP8+ population defined as non-MGE cells in the postnatal cortical streams in both human and piglet brains.

a. Wide-field images of DCX+ cells expressing COUP-TFII and SP8 in the anterior and posterior cingulated cortex (CC), ventral streams, and temporal cortex (TC) of the P0 piglet brain. Scale bar, 100µm.

b. DCX+ subpopulations in each migratory stream in P0 piglet brains express different transcription factors enriched in ventral telencephalic origins, including NKX2.1 and LHX6 (associated with the MGE) or COUP-TFII and SP8 (associated with the non-MGE, likely CGE identity). Scale bar, 50µm.

c. Quantification of DCX+ cells expressing selected TFs. Two-tailed unpaired t-test. The data are presented as mean ± SEM of counts performed on n=3 cases. Source Data Supplementary Fig.19 shows sample size and p-values.

d. Pie graph showing the proportion of DCX+ cells expressing NKX2.1 and LHX6 (light red; MGE-associated) and of DCX+ cells expressing COUP-TFII and/or SP8 (light green; non-MGE associated) in each migratory stream. The population represented as gray could be other CGE or LGE-associated cells, immature excitatory cells, or non-neuronal cells.

e. DCX+ subpopulations in each migratory stream in neonatal human brains express different transcription factors enriched in ventral telencephalic origins, including NKX2.1 and LHX6 or COUP-TFII and SP8. Scale bar, 50µm

f. Quantification of subpopulations of DCX+ cells, differentiated by selected transcription factor (TF) expression, across anterior-posterior migratory streams in the term human brain. Two-tailed unpaired t-test. The data are presented as mean ± SEM of counts performed on n=3 cases. Source Data Supplementary Fig.19 shows sample size and p-values.

g. Pie graph showing the proportion of DCX+ cells expressing NKX2.1 and LHX6 (light red; MGE-associated) and of DCX+ cells expressing COUP-TFII and/or SP8 (light green; non-MGE associated) in each migratory stream. The population represented as gray could be other CGE or LGE-associated cells, immature excitatory cells, or non-neuronal cells.

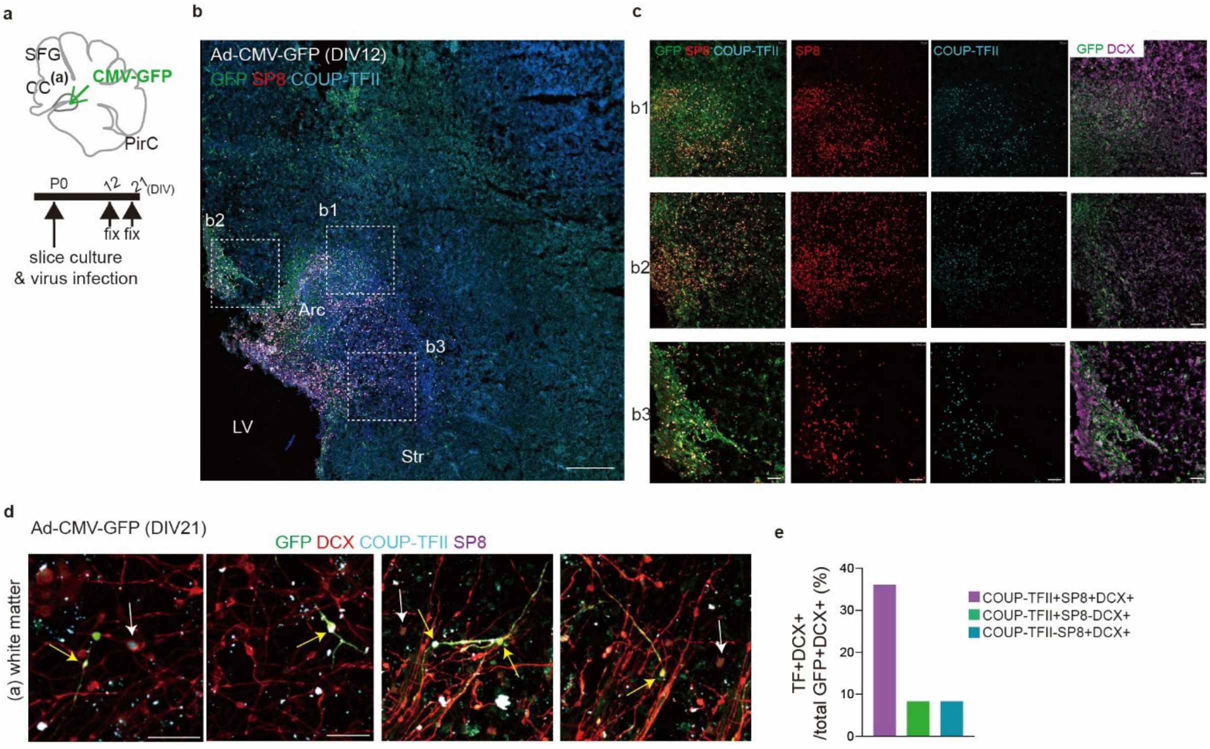

Supplementary Figure 20. Arc-derived cells expressing COUP-TFII and/or SP8.

a. Experimental design for extended time-lapse imaging of the P0 piglet brain. Ad-CMV-GFP virus is microinjected into the Arc. Slices are collected to perform post-hoc immunostaining at DIV 12 and 21.

b. Wide-field image of the immunostained slices collected at DIV12. The dashed white boxes ((b1-3)) are analyzed in c. Scale bar, 500 µm.

c. Confocal images immunostained with antibodies of GFP, DCX, COUP-TFII, and SP8. Arc-derived cells marked as GFP and DCX express COUP-TFII and/or SP8 in Arc ((b1)), the dorsal regions ((b2)), and the ventral region ((b3)). Scale bar, 30 µm. This experiment has been repeated three times (a-c).

d. Arc-derived cells marked as GFP and DCX express COUP-TFII and/or SP8 in the white matter closed to cingulate cortex (CC; (a) in a) at DIV21. Scale bar, 30 µm.

e. Quantification of the proportion of GFP+DCX+ cells expressing COUP-TFII and/or SP8 in the total GFP+DCX+ cells. A total of 42 GFP+DCX+ cells in the white matter and gray matter of the cortex at DIV21 were analyzed.

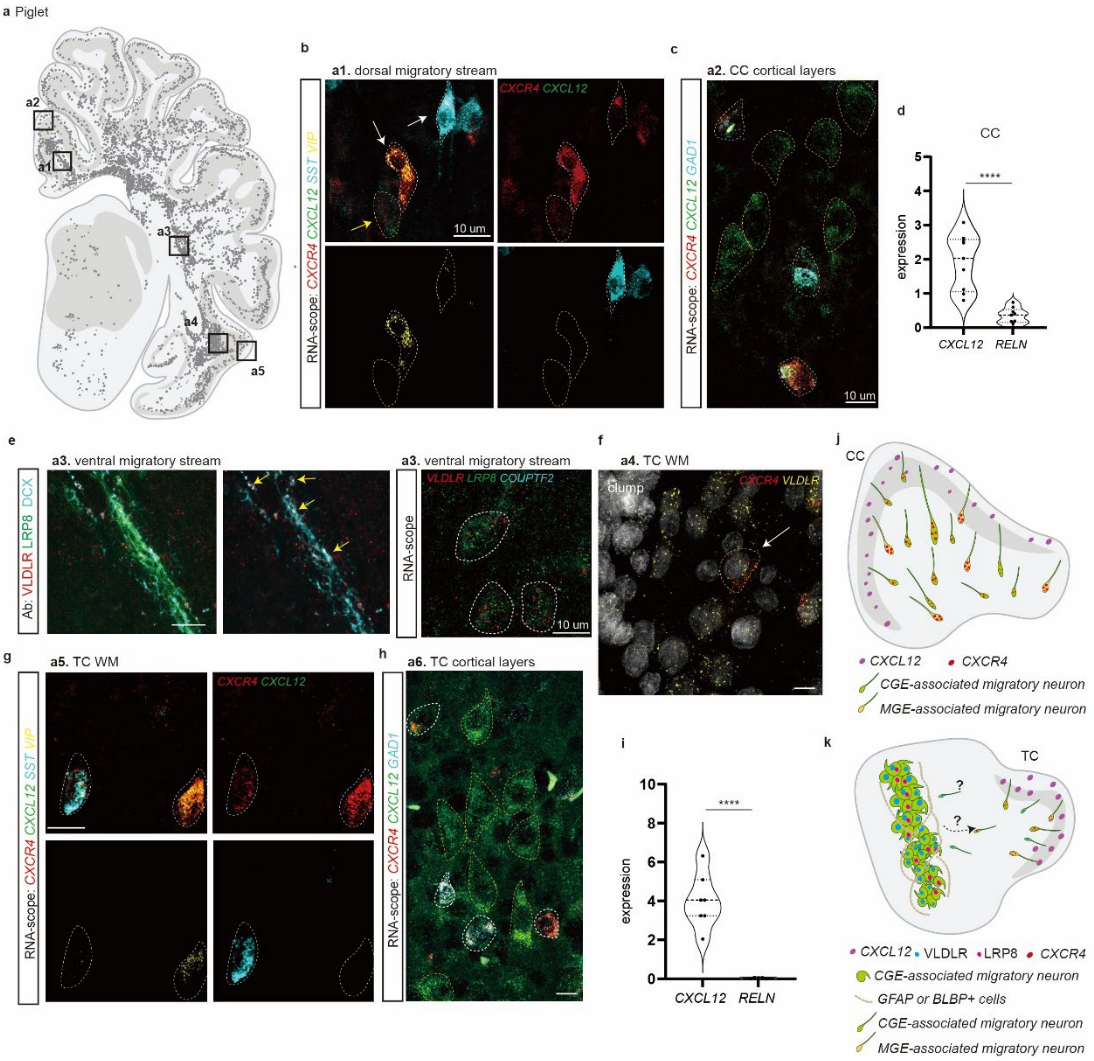

Supplementary Figure 21. Regionally distinct expression of migratory-related receptors in postnatal cortical migratory streams.

a. Mapping of DCX+ cells (gray) in the P0 piglet brain. The black-lined boxes were analyzed in (b-i).

b. RNA-scope of mRNA for *CXCR4*, *CXCR12*, *SST*, and *VIP* in **a1**, dorsal migratory stream. The individually migrating *SST*+ and *VIP*+ cells express CXCR4. Scale bar, 10 µm. This experiment has been repeated three times.

c-d, Left (c), a confocal image showing a higher expression of *CXCL12*, a ligand for CXCR4 in the cingulate cortex (CC) (**a2**) Scale bar, 10 µm. Right, (d), Quantification of expression area of CXCL12 puncta in the cingulate cortex (CC). Two-tailed unpaired t-test (****$p < 0.0001$). The data are presented as mean ± SEM of counts performed on n=3 cases in three independent experiments.

e. Left, immunostaining with VLDLR and LRP8 antibodies in **a3**, ventral migratory streams from the Arc. The yellow arrows indicate DCX+ cells expressing VLDLR. Scale bar, 30 µm. Right, RNA-scope showing a population with co-expression of *VLDLR*, *LRP8*, and *COUP-TFII* in ventral migratory streams. Scale bar, 10 µm. This experiment has been repeated three times.

f. *VLDLR*+ cell population and *CXCR4*+ cell population are observed together in the white matter (WM) of the temporal cortex (TC) (a4). Scale bar, 10 µm. This experiment has been repeated three times.
g. Individually migrating *SST*+ and *VIP*+ cells observed in the WM of TC (**a4**) express *CXCR4*. Scale bar, 10 µm.
h-i, Left (h), a confocal image showing a higher expression of *CXCL12*, a ligand for CXCR4 in the temporal cortex (TC) (**a5**) Scale bar, 10 µm. Right, (i), Quantification of expression area of CXCL12 puncta in the TC. Two-tailed unpaired t-test (****$p < 0.0001$). The data are presented as mean ± SEM of counts performed on n=3 cases in three independent experiments.
j-k, Schematics showing regionally distinct expression of migratory-related receptors in postnatal cortical migratory streams.

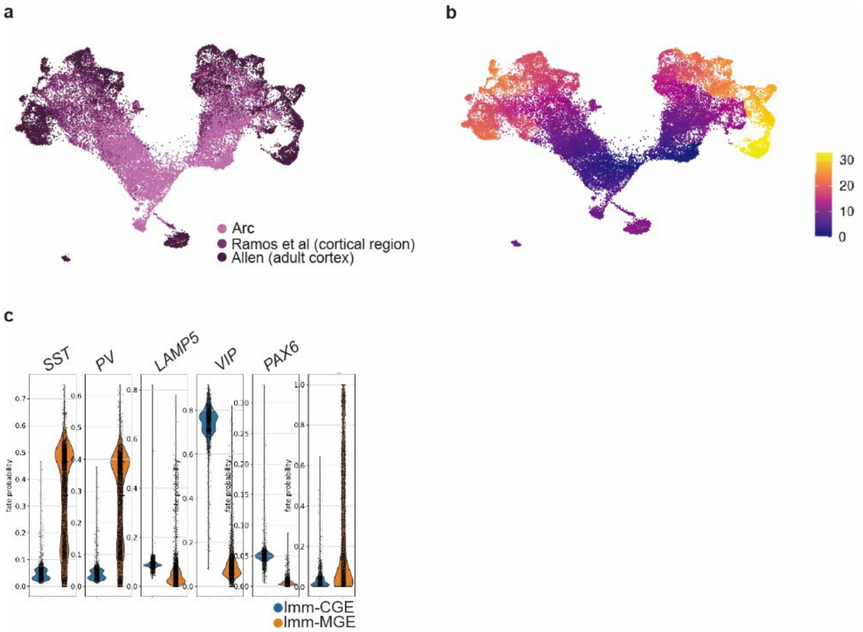

Supplementary Figure 22. Specification of Arc-derived interneuron into the cortical interneuron.

a. The adult cortical interneuron subtypes used in our study are derived from the open resources produced by the Allen Institute. It includes single-nucleus transcriptomes from the middle temporal gyrus, anterior cingulated cortex, primary visual cortex, primary motor cortex, primary somatosensory cortex, and primary auditory cortex. The perinatal datasets cover the single-nucleus RNA sequencing from the human Arc at GW 30-39 and from the human developing cortex at GW 17-41[25]. The different cortical interneuron subtypes in the integrated dataset are shown. Distribution of datasets in integrated UMAP space.

b. Monocle3 derived pseudotime of Arc-derived CGE and MGE interneurons visualized by UMAP.

c. Fate probabilities of immature-(Imm-) interneuron clusters from the Arc. Immature CGE neurons from the Arc are primarily committed to cortical fate, *VIP*+ and/or *CALB2*+ interneurons, rather than *LAMP5*+ and *PAX6*+ interneurons, suggesting that subtype-specific contribution of the Arc into the cortical interneuron population. Immature MGE neurons are committed to cortical *SST*+, *PV*+ interneurons, and striatal interneurons.

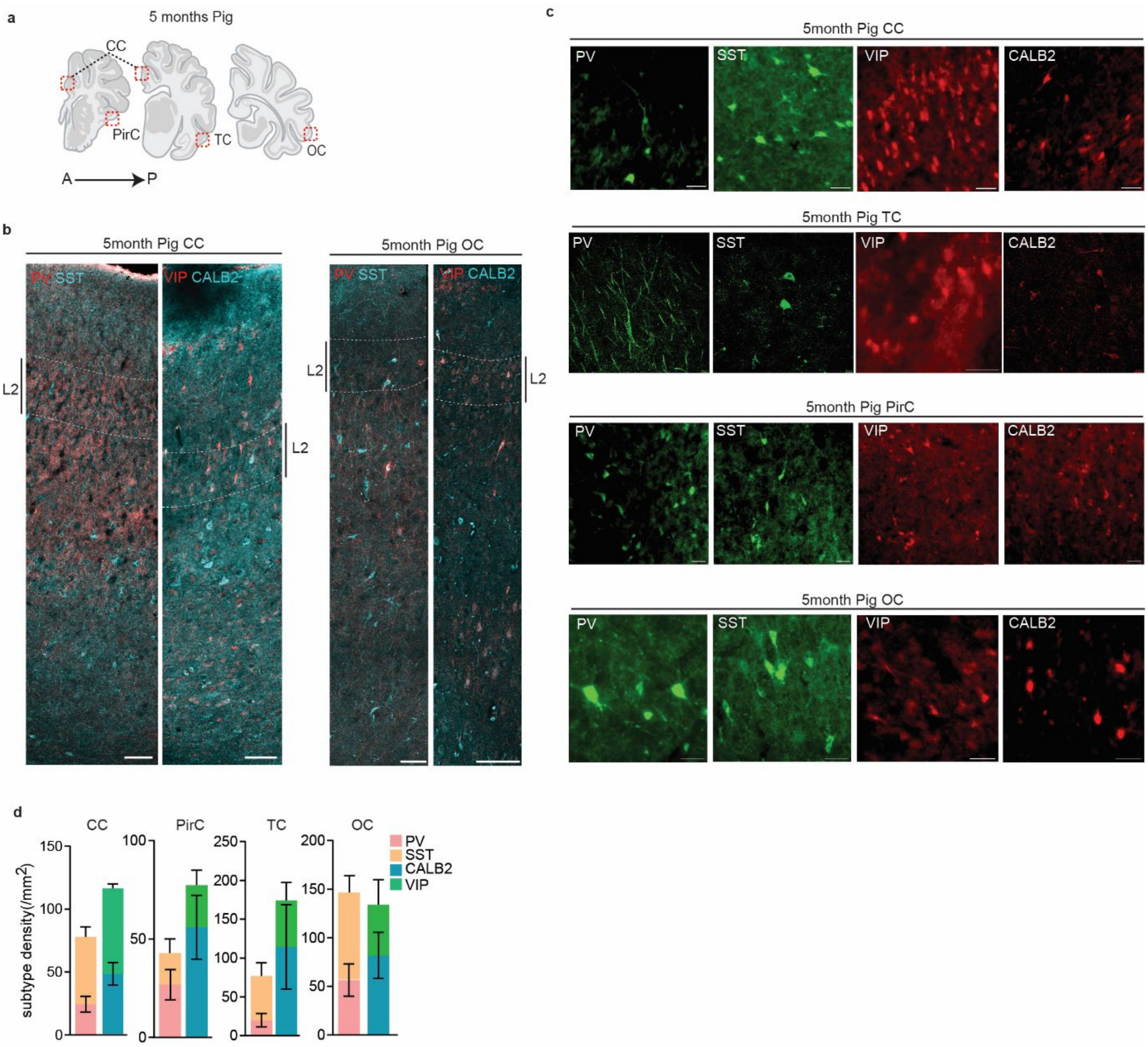

Supplementary Figure 23. Regional distribution of cortical interneuron subtypes in the 5-month pig brain.

a. Schematic illustrating anterior and posterior coronal sections of a 5-month pig brain. Red boxed areas indicate cingulate cortex (CC), piriform cortex (PirC), temporal cortex (TC), and occipital cortex (OC). CC, PirC, and TC are the main destinations of Arc-derived migratory neurons, while OC is less associated with Arc-derived migratory streams.

b. The cortical regions of the 5-month brain immunostained for interneuron markers. Left: PV (red)/SST (cyan) and VIP (red)/ CALB2 double-immunostained neocortical section from the cingulate cortex (CC). Right: PV (red)/SST (cyan) and VIP (red)/ CALB2 double-immunostained neocortical section from the occipital cortex (OC). The white dotted line delineates the border of cell-dense regions of the cortical layer 2. Some VIP+ neurons co-expressed CALB2. Scale bar, 100µm. This experiment has been repeated three times.

c. High magnification images of each cortical area in (A) show that VIP+ interneurons are abundant in the cingulate cortex (CC) and temporal cortex (TC). Scale bar, 50µm.

d. The density of each population is measured across cortical layers. CGE-subpopulation expressing VIP and/or CALB2 is abundant in the Arc-associated cortical regions, including

CC, PirC, TC, but not OC. The data are presented as mean ± SEM of counts performed on n=1 cases in three independent experiments. Source Data Supplementary Fig.23 shows the sample size.

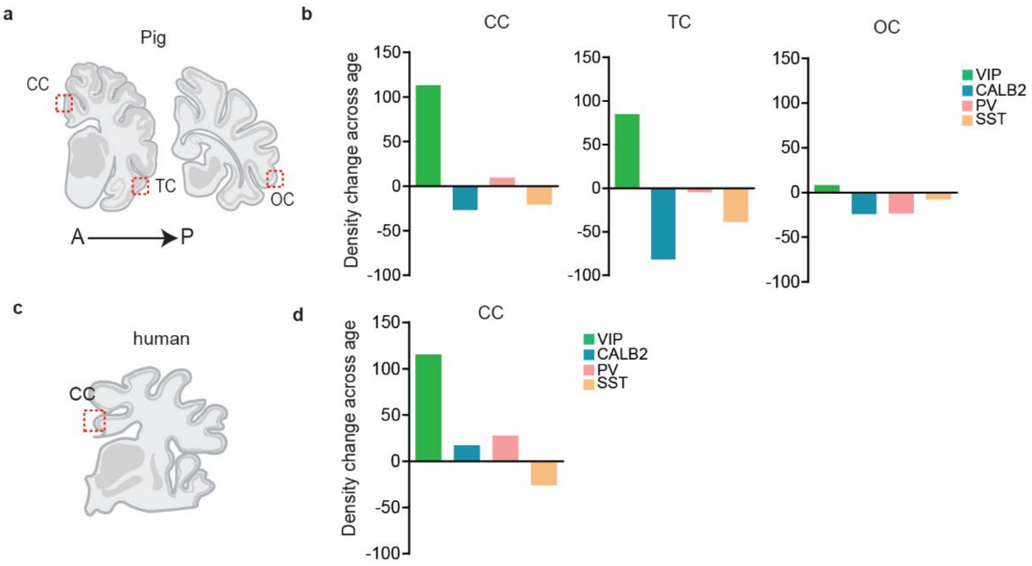

Supplementary Figure 24. Postnatal changes in interneuron composition in Arc-associated cortical regions.

a. Schematic illustrating coronal sections of the pig brain. Red boxed areas indicate cingulate cortex (CC), temporal cortex (TC), and occipital cortex (OC). CC, PirC, and TC are the main destinations of Arc-derived migratory neurons, while OC is less associated with Arc-derived migratory streams.

b. The density changes of each interneuron subclass in CC, TC, and OC between the 5 months (n=1 in three independent experiments) and 1 year of pigs (n=2 in three independent experiments). The values are the changes from the mean values at each time point. CC and TC exhibit dynamic postnatal changes in interneuron compositions, especially VIP+ neurons. OC shows less change in interneuron compositions**.**

c. Schematic illustrating coronal sections of the human brain. Red boxed areas indicate cingulate cortex (CC).

d. The density changes of each interneuron subclass in CC between the 7 months and 15 years of humans (each n=1 in three independent experiments). The values are the changes from the mean values at each time point. Like the pig CC, human CC exhibits dynamic postnatal changes of interneuron compositions, especially VIP+ neurons.

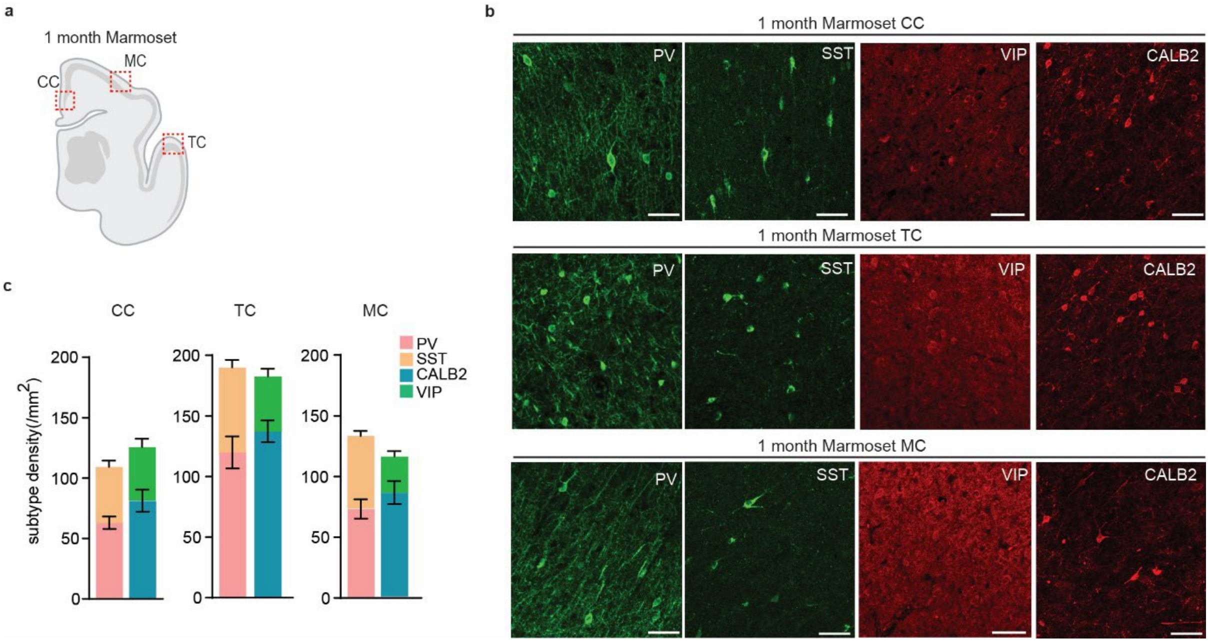

Supplementary Figure 25. Regional distribution of cortical interneuron subtypes in 1-month marmoset brain.

a. Schematic illustrating a coronal section of a 1-month marmoset brain. Red boxed areas indicate cingulate cortex (CC), motor cortex (MC), and temporal cortex (TC).

b. The cortical regions of the 1-month brain immunostained for interneuron markers. Scale bars, 30μm. VIP+ neurons are not abundant across cortical regions.

c. The density of each population is measured across cortical layers. The CGE subpopulation densities expressing VIP or CALB2 are similar to the densities of MGE subpopulations expressing PV or SST. Source Data Supplementary Fig.25 shows the sample size.

Supplementary Table1. Comparative structural features across species.

	brain weight (g)		gyrification index (GI)		gray matter thickness (mm)	
Age	newborn	adult	newborn	adult	newborn	adult
Home Sapiens	350-400	1300-1400	1.92±0.06	2.8	1.45±0.02	3.67±0.04
Pan troglodytes	136	384	1.84±0.03	2.1	1.41±0.02	2.5
Sus Scrofa	32.5	180	1.86±0.03	2.16	1.34±0.02	1.56±0.02
Macca Mulatta	54	90-97	1.76±0.04	1.79	1.81±0.03	2.37±0.19
Callithrix jacchus	3.4	8	1.08±0.05	1.17	1.45±0.02	1.6
Mus Musculus	0.2	0.4	1.01±0.01	1.03	0.36±0.01	0.8-0.9

Supplementary Table2. Comparative Arc features across species.

	Arc area ratio (%)	tier ratio in wedge area		BV ratio in wedge area	Two-way MANOVA analysis
		tier1	tier2-3		
Home Sapiens	0.63±0.04	0.13±0.01	0.87±0.01	0.025±0.007	NA
Sus Scrofa	0.44±0.12	0.22±0.02	0.77±0.02	0.011±0.001	Pillai's, F(3,4) = 3.98, p=0.1075
Callithrix jacchus	0.18±0.08	0.97±0.01	0.02±0.01	0.003±0.001	Pillai's, F(3,6) = 398.82, p = 6.21x10-7***
Mus Musculus	0.16±0.04	0.92±0.04	0.07±0.04	0.003±0.001	Pillai's, F (3,5) = 94.2, p = 8.07x10-5***

Supplementary Table3. Clinical and experimental demographics of collected human specimens

	Case No.	Age	Gender	Clinical History	Neuropathological diagnosis	Experimental Use
Human	1	GW22	M	spontaneous abortic	Control	immunostaining with DCX and a-SMA antibodies
	2	GW30	M	diaphragmatic herni	Control	immunostaining with DCX and a-SMA antibodies
	3	GW30-3/7weeks	F	autopsy	Control	single nucleus RNA sequencing
	4	GW39+2weeks	M	autopsy	Control	single nucleus RNA sequencing
	5	GW37+2 days	F	autopsy	Control	Nissl staining/TFs quantification/measurement of Arc area and BV ratio
	6	0 day	F	left renal hypoplasia and right renal agenesis	Control	Nissl staining/RNAscope/TFs quantification/measurement of Arc area and BV ratio
	7	GW37	F	Respiratory failure	Control	DCX mapping/TFs quantification/measurement of Arc area and BV ratio
	8	GW36+2 weeks	F	cardiac anomaly	Control	Nissl staining/RNAscope/TFs quantification/measurement of Arc area and BV ratio
	9	40 days	F	autopsy	Control	TF quantification
	10	7 months	M	wiskott aldrich	Control	DCX intensity quantification, Immunostaining with DCX,GFAP, MKI67 antibodies
	11	2 years	M	Leukemia	Control	Immunostaining with DCX and GFAP antibodies
	12	15 years	M	autopsy	Control	Immunostaining with DCX and GFAP antibodies, interneuron quantification
	13	25 years	M	autopsy	Control	interneuron quantification

Supplementary Table4. Experimental demographics of collected animal specimens

	Case No.	Age	Gender	Source	Neuropathological diagnosis	Experimental Use
Chimpanzee	1	0 yr	M	National Chimpanzee Brain Resource	Control	Nissl staining/measurement of Arc area/immunostaining
	2	0 yr	U		Control	Nissl staining/measurement of Arc area/immunostaining
Sheep	1	E135	U	Maastricht University Medical Center	Control	Nissl staining/measurement of Arc area/immunostaining
Marmoset	1	E144	U	University of Cambridge Marmoset Breeding Colony	Control	Nissl staining/immunostaining
	2	P0	U		Control	Nissl staining/measurement of Arc area/immunostaining
	3	P0	U		Control	Nissl staining/measurement of Arc area/immunostaining
	4	P0	U		Control	Nissl staining/measurement of Arc area/immunostaining
Pig	1	E62	U	Swine Teaching and Research Center at the University of California, Davis	Control	Nissl staining/TFs imaging
	2	E89	U		Control	Nissl staining/DCX Intensity quantification/immunostaining
	3	E89	U		Control	Nissl staining/DCX Intensity quantification/immunostaining
	4	E100	U		Control	Nissl staining/DCX Intensity quantification/immunostaining
	5	E100	U		Control	Nissl staining/DCX Intensity quantification/immunostaining
	6	P0	M		Control	Nissl staining/DCX Intensity quantification/immunostaining
	7	P0	M		Control	Nissl staining/DCX Intensity quantification/immunostaining
	8	P0	F		Control	Nissl staining/DCX intensity quantification/measurement of Arc features/Immunostaining
	9	P2	F		Control	Nissl staining/DCX intensity quantification/measurement of Arc features/Immunostaining
	10	P16	M		Control	Nissl staining/DCX intensity quantification/measurement of Arc features/Immunostaining
	11	P28	M		Control	Nissl staining/DCX intensity quantification/measurement of Arc features/Immunostaining
	12	P28	F		Control	Nissl staining/DCX intensity quantification/measurement of Arc features/Immunostaining
	13	5 month	F		Control	Nissl staining/DCX intensity quantification/ Immunostaining
	13	1 yr	F		Control	Immunostaining
	14	1 yr	F		Control	Immunostaining
Mouse	1	P0	M	UCSF	Control	Nissl staining/measurement of SVZ area/immunostaining
	2	P0	M		Control	Nissl staining/measurement of SVZ area/immunostaining
	3	P0	F		Control	Nissl staining/measurement of SVZ area/immunostaining

Movie S1.

Light sheet imaging of a clarified P0 piglet brain from anterior to posterior. The black signal is DCX immunoreactivity. The stars (*) indicate dorsal streams of DCX+ cells from the Arc to the cingulate cortex and ventral streams of DCX+ cells from the Arc to the piriform cortex.

Movie S2.

3D rendering of DCX+ cells (green) and BLBP+ cells (magenta) in dorsal streams from P0 piglet Arc.

Movie S3.

3D rendering of DCX+ cells (green) and BLBP+ cells (magenta) in ventral streams from P0 piglet Arc.

Movie S4.

Time-lapse imaging showing migrating neurons in P0 piglet organotypic slice culture. The area imaged is the dorsal side of the Arc. The cell-dense region is the Arc, and the white line delineates the boundary of the Arc. Note that labeled cells (•) are traveling in a dorsal direction, away from the Arc. The movie spans 34 hours.

Movie S5.

Time-lapse imaging showing migrating neurons in P0 piglet organotypic slice culture. The area imaged is the dorsal side of the Arc. The cell-dense region is the Arc, and the white line delineates the boundary of the Arc. Note that labeled cells (•) are traveling in a dorsal direction, away from the Arc. The movie spans 34 hours.
