# Supplementary material for: An expanded subventricular zone supports postnatal cortical interneuron migration in gyrencephalic brains
Source: Nat Neurosci. 2025 Jul 14;28(8):1598–609. doi: 10.1038/s41593-025-01987-2 (PMC12321571; doi:10.1038/s41593-025-01987-2)
Supplement: Supplementary file 2 — Reporting Summary [file 41593_2025_1987_MOESM2_ESM.pdf]

nature portfolio

Corresponding author(s): Mercedes Paredes

Last updated by author(s): Mar 5, 2025

Reporting Summary

Nature Portfolio wishes to improve the reproducibility of the work that we publish. This form provides structure for consistency and transparency in reporting. For further information on Nature Portfolio policies, see our Editorial Policies and the Editorial Policy Checklist.

Statistics

For all statistical analyses, confirm that the following items are present in the figure legend, table legend, main text, or Methods section.

n/a	Confirmed	
☐	☒	The exact sample size (*n*) for each experimental group/condition, given as a discrete number and unit of measurement
☐	☒	A statement on whether measurements were taken from distinct samples or whether the same sample was measured repeatedly
☐	☒	The statistical test(s) used AND whether they are one- or two-sided<br>*Only common tests should be described solely by name; describe more complex techniques in the Methods section.*
☒	☐	A description of all covariates tested
☐	☒	A description of any assumptions or corrections, such as tests of normality and adjustment for multiple comparisons
☐	☒	A full description of the statistical parameters including central tendency (e.g. means) or other basic estimates (e.g. regression coefficient) AND variation (e.g. standard deviation) or associated estimates of uncertainty (e.g. confidence intervals)
☐	☒	For null hypothesis testing, the test statistic (e.g. *F*, *t*, *r*) with confidence intervals, effect sizes, degrees of freedom and *P* value noted<br>*Give P values as exact values whenever suitable.*
☒	☐	For Bayesian analysis, information on the choice of priors and Markov chain Monte Carlo settings
☒	☐	For hierarchical and complex designs, identification of the appropriate level for tests and full reporting of outcomes
☐	☒	Estimates of effect sizes (e.g. Cohen's *d*, Pearson's *r*), indicating how they were calculated

Our web collection on statistics for biologists contains articles on many of the points above.

Software and code

Policy information about availability of computer code

Data collection	GE MR950 7T scanner was used for MRI images. LAS-X (Leica) software was used for acquiring the microscopy data. NovaSeq 6000 (Illumina) was used for sequencing reads.
Data analysis	MRI images were analyzed with ITK-SANP software and NEOCIVET v2.0 pipeline. Fiji (imageJ, v2.14.0/1.54 f), Neurolucida software (MBF Bioscience, 2018 version), and HiPlex image registration software v2 (ACD) were used for image quantification and processing. and The GraphPad Prism (v6) was used for statistic analyses. Cell Ranger v7.1.0, CellBender v0.2.2., R4.4.1, DoubletFinder v2.0.3, ggplot2 v3.3.5, Seurat v5, scanpy v.1.9.6., and hdWGCNA package v.0.3.01. was used to analyze sequencing data.

For manuscripts utilizing custom algorithms or software that are central to the research but not yet described in published literature, software must be made available to editors and reviewers. We strongly encourage code deposition in a community repository (e.g. GitHub). See the Nature Portfolio guidelines for submitting code & software for further information.

Data

Policy information about availability of data

All manuscripts must include a data availability statement. This statement should provide the following information, where applicable:

- Accession codes, unique identifiers, or web links for publicly available datasets
- A description of any restrictions on data availability
- For clinical datasets or third party data, please ensure that the statement adheres to our policy

The data of this study are available on request from the corresponding author. The data described in this study are available on Figshare at https://doi.org/10.6084/m9.figshare.25055588. The newly generated sequencing data have been deposited in the Gene Expression Omnibus under accession number GSE255968. Source data are provided with this paper. The following public transcriptomic datasets were used to support this study: Ramos et al.25 data were downloaded from the Gene Expression Omnibus with the accession number GSE217511. Shi et al.22 data were downloaded with the accession number GSE135827. Transcriptomic datasets of adult human cortical regions were downloaded in the Adult Human Cortical SMART-Seq data at https://portal.brain-map.org/atlases-and-data/rnaseq/human-multiple-cortical-areas-smart-seq. All genomic analyses were performed using the GRCh38 human genome assembly. The following public neuroimaging datasets were used to support this study: the Developing Human Brain Project at https://www.developingconnectome.org/project/, the Marmoset Brain Mapping v3 at https://marmosetbrainmapping.org/, the Neonatal Mouse Brain Atlas a thttps://www.loni.usc.edu/research/atlases, and the NIH Blueprint NHP Atlas at https://www.blueprintnhpatlas.org/.

Research involving human participants, their data, or biological material

Policy information about studies with human participants or human data. See also policy information about sex, gender (identity/presentation), and sexual orientation and race, ethnicity and racism.

Reporting on sex and gender	Sex and gender were not used in any scenario as criteria for sample collection. Sex information from all de-identified samples were provided by the relevant brain and tissue banks.
Reporting on race, ethnicity, or other socially relevant groupings	No race, ethnicity or socially relevant groupings were performed in this study.
Population characteristics	We collected human brain samples from gestational week 30 to adulthood. The detailed age information for each sample can be identified in Supplementary Table 3. Population characteristics of de-identified samples were provided by the relevant brain and tissue bank. No population characteristics other than age and sex were used in our analysis.
Recruitment	De-identified tissue samples were collected with previous patient consent in strict observance of the legal and institutional ethical regulations. No active recruitment was implemented. Postmortem human brain samples were collected from UCSF (see Supplementary Table 3 for details). There is no self-selection bias in these inclusion criteria, however, we cannot rule out possible bias from the patient populations or unidentified disease conditions. This was performed by the clinical and no recruitment criteria were used.
Ethics oversight	Tissue was collected with previous patient consent in strict observance of the legal and institutional ethical regulations of the University of California San Francisco Committee on Human Research. Protocols were approved by the Human Gamete, Embryo, and Stem Cell Research Committee and the Committee on Human Research (institutional review board) at the University of California, San Francisco.

Note that full information on the approval of the study protocol must also be provided in the manuscript.

Field-specific reporting

Please select the one below that is the best fit for your research. If you are not sure, read the appropriate sections before making your selection.

☒ Life sciences ☐ Behavioural & social sciences ☐ Ecological, evolutionary & environmental sciences

For a reference copy of the document with all sections, see nature.com/documents/nr-reporting-summary-flat.pdf

Life sciences study design

All studies must disclose on these points even when the disclosure is negative.

Sample size	No statistical methods were used to pre-determine sample sizes but our sample sizes are similar to those reported in previous publications (Paredes at al., Science 2016; paredes at al., Science 2022; Morton et al., Sci Transl Med 2017; Porter at al., Stem Cell Reports 2022).
Data exclusions	When analyzing the migratory behaviors of GFP+ cells (Figure 3), GFP+ cells densely packed and with large morphologies were excluded. In addition, Dcx+ cells with less than 3 dots of mRNA (Figure 4) were not included for analyses.
Replication	Data shown in representative experiments was repeated with similar results in at least 3 independent experiments. All attempts at replication were successful. Sample size for other experiments were indicated in Figure legends. Time-lapse live imaging and long-term slice culture experiments were repeated from in three different animals.

Randomization	For two experiments used piglet tissues, time-lapse live imaging and long-term slice culture, piglet organotypic slices from a same block were randomly grouped. For other experiments, randomization is not relevant since there are no experimental and control groups. And all samples from each animal were categorized according to their biological age.
Blinding	Blinding of different regions (dorsal and ventral regions from the Arc) was used for imaging quantification, time-lapse live imaging, and spatial transcriptomic. Blinding was not used or was not relevant for other experiments. It is not possible to completely blind investigators to age or region due to obvious structural and cellular differences between samples. There are a clear differences in brain size, cell size, and cell density across animals and developmental ages. Samples were not quantified in order of age, but were evaluated in a random order.

Reporting for specific materials, systems and methods

We require information from authors about some types of materials, experimental systems and methods used in many studies. Here, indicate whether each material, system or method listed is relevant to your study. If you are not sure if a list item applies to your research, read the appropriate section before selecting a response.

Materials & experimental systems

n/a	Involved in the study
☐	☒ Antibodies
☒	☐ Eukaryotic cell lines
☒	☐ Palaeontology and archaeology
☐	☒ Animals and other organisms
☒	☐ Clinical data
☒	☐ Dual use research of concern
☒	☐ Plants

Methods

n/a	Involved in the study
☒	☐ ChIP-seq
☒	☐ Flow cytometry
☐	☒ MRI-based neuroimaging

Antibodies

Antibodies used

anti-ALDH1L1 (mouse, N103/39 clone, Antibodies Inc, 75-140, 1:500 dilution)
anti-a-SMA (mouse, 1A4 clone, Sigma, a2547, 1:500 dilution)
anti-COUP-TFII (mouse, H7147 clone, R&D Systems, PP-H7147-00, 1:100 dilution)
anti-BLBP (rabbit, Abcam, ab32423, 1:500 dilution).
anti-doublecortin (rabbit, Cell Signaling, 4604, 1:200 dilution).
anti-doublecortin (guinea Pig, Millipore, ab2253, 1:200 dilution).
anti-DLX2 (mouse, 3B6 clone, Santa Cruz, sc-393879, 1:200 dilution).
anti-EMX1 (mouse, 3H2 clone, Santa Cruz, sc-398115, 1:200 dilution).
anti-FOXP2 (rabbit, Millipore, ABE74, 1:500 dilution).
anti-GFAP (chicken, Abcam, ab4674, 1;750 dilution).
anti-GFP (chicken IgY, Aves labs, GFP-1020, 1:1000 dilution).
anti-GSH2 (rabbit, Abcam, ab26255, 1:200 dilution).
anti-IBA1 (goat, Abcam, ab5076, 1:250 dilution).
anti-LHX6 (mouse, 6F3 clone, Santa Cruz, sc-271433, 1:200 dilution).
anti-MEIS2 (mouse, 2F4 clone, Sigma,WH0004212M1, 1:500 dilution).
anti-Mki67 (rabbit, Abcam, ab15580, 1:500 dilution).
anti-NeuN (chicken, Millipore, ABN91, 1:500 dilution).
anti-NKX2.1 (rabbit, Santa Cruz, sc-13040, 1:100 dilution).
anti-Olig2 (rabbit, Millipore, AB9610, 1:200 dilution).
anti-MBP (rat, 2D1 clone, Millipore, MAB386, 1:200 dilution).
anti-PAX6 (mouse, 1B5 clone, Abcam, ab78545, 1:200 dilution).
anti-PROX1 (goat, R&D, AF2727, 1:200 dilution).
anti-PSA-NCAM (mouse, 5A5 clone, Millipore, MAB5324, 1:500 dilution).
anti-SCGN (mouse, EPR23393-30 clone, Abcam, ab244219, 1:500 dilution)
anti-SP8 (goat, Santa Cruz, sc-10466, 1:100 dilution).
anti-TSHZ1 (rabbit, Abcam, ab140196, 1:200 dilution).
anti-CXCR4 (rabbit, Abcam, ab124824, 1:100 dilution).
anti-VLDLR (rabbit, Abcam, ab203271, 1:200 dilution).
anti-PV (rabbit, Swant, PV27, 1:500 dilution).
anti-PV (mouse, 1B2 clone, Swant, PV235, 1:200 dilution).
anti-SST (rat, Abcam, ab30788, 1:200 dilution).
anti-VIP (rabbit, Abcam, ab22736, 1:200 dilution).
anti-CALB2 (rabbit, Swant, 7697, 1:200 dilution).
anti-CALB2 (guinea Pig, Swant, CRgp7, 1:200 dilution).
Biotin-SP-AffiniPure Donkey Anti-Mouse IgG (H+L) (Jackson ImmunoResearch, 715065151, 1:250 dilution).
Biotin-SP-AffiniPure Donkey Anti-Rabbit IgG (H+L) (Jackson ImmunoResearch, 711065152, 1:250 dilution).
Biotin-SP-AffiniPure Donkey Anti-Goat IgG (H+L) (Jackson ImmunoResearch, NC9494985, 1:250 dilution).
Biotin-SP-AffiniPure Donkey Anti-Guinea Pig IgG (H+L) (Jackson ImmunoResearch, 706065148, 1:250 dilution).
Alexa Fluor® 488 AffiniPure Goat Anti-Mouse IgG (H+L) (Abcam, 150105, 1:500 dilution).
Alexa Fluor® 555 AffiniPure Goat Anti-Mouse IgG (H+L) (Invitrogen, A21424, 1:500 dilution).
Alexa Fluor® 647 AffiniPure donkey Anti-Mouse IgG (H+L) (Invitrogen, A31571, 1:500 dilution).

	Alexa Fluor® 488 AffiniPure Donkey Anti-Rabbit IgG (H+L) (Invitrogen, A21206, 1:500 dilution).<br>Alexa Fluor® 647 AffiniPure Goat Anti-Rabbit IgG (H+L) (Abcam, 150079, 1:500 dilution).<br>Alexa Fluor® 647 AffiniPure Donkey Anti-Chickne IgY (Abcam, 63507, 1:500 dilution).<br>Alexa Fluor® 594 AffiniPure donkey Anti-goat IgG (H+L) (Abcam, 150132, 1:500 dilution).<br>Alexa Fluor® 594 AffiniPure Goat Anti-Guinea Pig IgG (H+L) (Invitrogen, A11076, 1:500 dilution).<br>Tyramide-conjgated Fluorophore FITC (Akoya, NEL701A001KT, 1:100 dilution).<br>Tyramide-conjgated Fluorophore Cy3(Akoya, NEL704A001KT, 1:100 dilution).<br>Tyramide-conjgated Fluorophore Cy5 (Akoya, NEL705A001KT , 1:100 dilution).
Validation	anti-SMA, anti-COUP-TFII, anti-doublecortin, anti-DLX2, anti-EMX1, anti-GFAP, anti-GFP, anti-GSH2, anti-LHX6, anti-Mki67, anti-NeuN, anti-NKX2.1, anti-Olig2, anti-PAX6, anti-PROX1, anti-PSA-NCAM, anti-SCGN, anti-SP8, anti-CXCR4, anti-VLDLR, anti-PV, anti-SST, anti-VIP, anti-CALB2, and all secondary antibodies were used in our previous studies and validated in Human, Pig, and Mouse brains (Paredes et al, 2016; Paredes et al, 2022; Nascimento et al, 2024). anti-FOXP2 and anti-MEIS2 were validated in previous study and validated in Human, Macaque, Pig, and Mouse brains (Schmitz et al, 2022). Anti-ADH1L1 has been validated for WB, IHC, and IF on Human, Mouse, and Rat tissue by Manufacturer. Anti-IBA1 has been validated for use in IHC, IF, and WB on human, mouse, and rat tissues by manufacturer. anti-MBP has been validated for use in WB, IHC, IF, and FC applications. It recognizes the myelin basic protein (MBP) in human, rat, and mouse tissues, with specific staining in oligodendrocytes and myelin by manufacturer.<br>All antibodies used in IHC analysis have been validated by the manufacture to be suitable for this application. Each antibody is thus internally controlled by expected anatomical distribution and subcellular localization.

Animals and other research organisms

Policy information about studies involving animals; ARRIVE guidelines recommended for reporting animal research, and Sex and Gender in Research

Laboratory animals	Mice (male and female) were maintained on the C57BL/6J background. Both male and female at postnatal day 0 of age were used for analyses. All mice were maintained on temperature 20 ± 2C and a 14-h light, 10-h dark cycle, with access to food and water. Postmortem chimpanzee brains at birth were provided by the National Chimpanzee Brain Resource. Sheep brains from embryonic (E) 135 were collected in the certified sheep facility at Maastricht University Medical Center (n=1). Pig brains from E62, E89, E100, postnatal day (P) 0-2, P16, P28, 5 months, and 1 year of age (n=2-5) were collected at the Swine Teaching and Research Center at the University of California, Davis. Postmortem E144 and P0 marmoset brains (n=1-3) were collected immediately following euthanasia for welfare purposes at the University of Cambridge Marmoset Breeding Colony.
Wild animals	No wild animals were used in this study.
Reporting on sex	Sex was not considered in our study. But, all experiments was performed from equal numbers of male and female pigs. We did not observe any sex-based molecular/cellular differences in the experiments.
Field-collected samples	All pigs were housed and cared for at the UC Davis Swine Center. A typical prenatal care/gestation diet will be provided daily. Pregnant pigs were moved to the maternal crate and housed individually one to two weeks before the expected farrowing (birth) date. The pigs had free access to water and diet in maternal crates. Pigs born from natural or assisted farrowing were nursed by their sows until 3 weeks of age. The pigs were weaned at week 4 after birth, transferred to, and group-housed in a nursery pen. Pigs had free access to a solid diet and water in the nursery pen. The health of sows and piglets were checked daily by research and farm staff and undergo routine farm care: teeth clipping, tail docking, castration, when necessary, ear notching, and iron injection within 3 days after birth.
Ethics oversight	All animal procedures conformed to the requirements of the Animal Welfare Act and were carried out under the Association of Assessment and Accreditation of Laboratory Animal Care International (AAALAC) approved conditions with protocols approved before implementation by the Institutional Animal Care and Use Committee (IACUC) at the University of California, Davis (IACUC#), at the University of California, San Francisco (IACUC #AN192603-01), and by the local Animal Welfare and Ethical Review Board at the University of Cambridge Marmoset Breeding Colony. Protocols and samples were approved by UCSF GESCR (Gamete, Embryo, and Stem Cell Research) Committee.

Note that full information on the approval of the study protocol must also be provided in the manuscript.

Plants

Seed stocks	N/A
Novel plant genotypes	N/A
Authentication	N/A

Magnetic resonance imaging

Experimental design

Design type	3D structural imaging of the neonatal piglet brain.
Design specifications	Fixed embryonic day 100 and postnatal day 0-aged piglet brains were used for MRI scanning. Total three hemispheres from the different animals were scanned.
Behavioral performance measures	The study did not involve behavior performance.

Acquisition

Imaging type(s)	Structural
Field strength	*Specify in Tesla*
Sequence & imaging parameters	*Specify the pulse sequence type (gradient echo, spin echo, etc.), imaging type (EPI, spiral, etc.), field of view, matrix size, slice thickness, orientation and TE/TR/flip angle.*
Area of acquisition	*State whether a whole brain scan was used OR define the area of acquisition, describing how the region was determined.*

Diffusion MRI ☐ Used ☒ Not used

Preprocessing

Preprocessing software	*Provide detail on software version and revision number and on specific parameters (model/functions, brain extraction, segmentation, smoothing kernel size, etc.).*
Normalization	*If data were normalized/standardized, describe the approach(es): specify linear or non-linear and define image types used for transformation OR indicate that data were not normalized and explain rationale for lack of normalization.*
Normalization template	*Describe the template used for normalization/transformation, specifying subject space or group standardized space (e.g. original Talairach, MNI305, ICBM152) OR indicate that the data were not normalized.*
Noise and artifact removal	*Describe your procedure(s) for artifact and structured noise removal, specifying motion parameters, tissue signals and physiological signals (heart rate, respiration).*
Volume censoring	*Define your software and/or method and criteria for volume censoring, and state the extent of such censoring.*

Statistical modeling & inference

Model type and settings	*Specify type (mass univariate, multivariate, RSA, predictive, etc.) and describe essential details of the model at the first and second levels (e.g. fixed, random or mixed effects; drift or auto-correlation).*
Effect(s) tested	*Define precise effect in terms of the task or stimulus conditions instead of psychological concepts and indicate whether ANOVA or factorial designs were used.*

Specify type of analysis: ☐ Whole brain ☐ ROI-based ☐ Both

Statistic type for inference (See Eklund et al. 2016)	*Specify voxel-wise or cluster-wise and report all relevant parameters for cluster-wise methods.*
Correction	*Describe the type of correction and how it is obtained for multiple comparisons (e.g. FWE, FDR, permutation or Monte Carlo).*

Models & analysis

n/a	Involved in the study
☐	☐ Functional and/or effective connectivity
☐	☐ Graph analysis
☐	☐ Multivariate modeling or predictive analysis

Functional and/or effective connectivity	*Report the measures of dependence used and the model details (e.g. Pearson correlation, partial correlation, mutual information).*
Graph analysis	*Report the dependent variable and connectivity measure, specifying weighted graph or binarized graph, subject- or group-level, and the global and/or node summaries used (e.g. clustering coefficient, efficiency, etc.).*

Multivariate modeling and predictive analysis

Specify independent variables, features extraction and dimension reduction, model, training and evaluation metrics.
